# Estimating the longitudinal association between pain characteristics and clinical outcomes in young people with mental ill-health

**DOI:** 10.1017/S0033291725101104

**Published:** 2025-07-30

**Authors:** Valerie A. Oosterwijk, Caroline X. Gao, Jana Menssink, Josh Nguyen, Kate Filia, Amity E. Watson, Helen Herrman, Sarah E. Hetrick, Alex G. Parker, Ian B. Hickie, Debra Rickwood, Patrick D. McGorry, Susan M. Cotton, Lianne Schmaal, Scott D. Tagliaferri

**Affiliations:** 1 Faculty of Medicine, Vrije Universiteit, Amsterdam, The Netherlands; 2 Orygen, Parkville, VIC, Australia; 3 Centre for Youth Mental Health, University of Melbourne, Melbourne, VIC, Australia; 4 Department of Epidemiology and Preventative Medicine, School of Public Health and Preventive Medicine, Monash University, Melbourne, VIC, Australia; 5 Department of Psychological Medicine, University of Auckland, Auckland, New Zealand; 6 Institute for Health and Sport, Victoria University, Melbourne, VIC, Australia; 7 Brain and Mind Centre, University of Sydney, Sydney, NSW, Australia; 8 Faculty of Health, University of Canberra, Canberra, ACT, Australia; 9 Headspace National Youth Mental Health Foundation, Melbourne, VIC, Australia; 10 School of Psychological Sciences, Monash University, Melbourne, VIC, Australia; 11 Turner Institute for Brain and Mental Health, Monash University, Melbourne, VIC, Australia

**Keywords:** adolescence, chronic pain, psychiatry, psychology, young adult, youth

## Abstract

**Background:**

Mental ill-health has a major impact on young people, with pain often co-occurring. We estimated the prevalence and impact of pain in young people with mental ill-health.

**Methods:**

Longitudinal data (baseline and three-month follow-up) of 1,107 Australian young people (aged 12–25 years) attending one of five youth mental health services. Multi-level linear mixed models estimated associations between pain characteristics (frequency, intensity, and limitations) and outcomes with false discovery rate (FDR) adjustment. Pain characteristics were baseline-centered to estimate if the baseline score (*between-participant effect*) and/or change from baseline (*within-participant effect)* was associated with outcomes.

**Results:**

At baseline, 16% reported serious pain more than 3 days, 51% reported at least moderate pain, and 25% reported pain-related activity limitations in the last week. Between participants, higher serious pain frequency was associated with greater anxiety symptoms (*β*[95%CI]: 0.90 [0.45, 1.35], FDR-p=0.001), higher pain intensity was associated with greater symptoms of depression (1.50 [0.71, 2.28], FDR-p=0.001), anxiety (1.22 [0.56, 1.89], FDR-p=0.002), and suicidal ideation (3.47 [0.98, 5.96], FDR-p=0.020), and higher pain limitations were associated with greater depressive symptoms (1.13 [0.63, 1.63], FDR-p<0.001). Within participants, increases in pain intensity were associated with increases in tobacco use risk (1.09 [0.48, 1.70], FDR-p=0.002), and increases in pain limitations were associated with increases in depressive symptoms (0.99 [0.54, 1.43], FDR-p<0.001) and decreases in social and occupational functioning (−1.08 [−1.78, −0.38], FDR-p=0.009).

**Conclusions:**

One-in-two young people seeking support for mental ill-health report pain. Youth mental health services should consider integrating pain management.

## Introduction

Youth mental ill-health represents a global crisis (McGorry et al., 2024). For example, depression and anxiety disorders are the most common mental ill-health disorders (World Health Organization, 2022), with a peak onset occurring during adolescence and young adulthood (Kessler et al., 2005). This has a significant impact on young people, including lower social and occupational functioning and reduced quality of life (Bowman, McKinstry, & McGorry, 2017; Cotton et al., 2022; Gibb, Fergusson, & Horwood, 2010; Jaycox et al., 2009). While many young people recover from mental ill-health and healthcare services provide support to them and their families, treatment outcomes have remained modest (Patel et al., 2023). Further understanding of the etiology of these disorders and barriers and facilitators to recovery is needed to effectively treat them (McGorry et al., 2024). The co-occurrence of mental and physical conditions, such as pain (Cotton et al., 2022; Dudeney et al., 2024; Slater et al., 2025; Victor et al., 2010), is often overlooked and may hold the key to improved treatment outcomes (Kroenke et al., 2008; Liu et al., 2024; Thielke, Fan, Sullivan, & Unützer, 2007).

Pain is defined as an unpleasant sensory and emotional experience associated with, or resembling that associated with, actual or potential tissue damage (Raja et al., 2020). In young people, pain might be underdiagnosed or undertreated (Friedrichsdorf et al., 2015; Hassett et al., 2013), with headache, musculoskeletal, back, abdominal, pelvic, and multisite pain most common (Chambers et al., 2024; Hirsch et al., 2020). Pain can be classified into acute and chronic pain. Acute pain is considered an appropriate response to tissue trauma or inflammatory-related processes, with many recovering within expected tissue healing times (Cohen, Vase, & Hooten, 2021). However, some individuals with acute pain go on to develop chronic pain, defined as pain lasting or recurring for three or more months (Treede et al., 2019). In the context of acute pain, the presence of mental ill-health predicts the transition to chronic pain in young people (Fisher, Monsell, Clinch, & Eccleston, 2024; Holley, Wilson, & Palermo, 2017; Rabbitts et al., 2020). One-in-five young people are estimated to live with chronic pain at any one time (Chambers et al., 2024), which impairs daily functioning and quality of life (Huguet & Miró, 2008; Hunfeld et al., 2001) and is associated with an increased prevalence of mental ill-health (Dudeney et al., 2024).

In the context of youth mental health services, 45% of young people presenting for mental health treatment experience pain that negatively impacts overall quality of life (Cotton et al., 2022), which could be any type of pain (e.g. acute versus chronic or any location). This is an important issue considering pain is associated with a reduced response to mental health treatment in adults (Kroenke et al., 2008; Liu et al., 2024; Thielke et al., 2007). Both mental ill-health and pain have been associated with key clinical outcomes for young people including severity of depression and anxiety (Filia et al., 2021; Slater et al., 2025), suicidal thoughts and behaviors (Filia et al., 2021; Hinze et al., 2019, 2021; Hinze, Karl, Ford, & Gjelsvik, 2023; Moller et al., 2022), substance (mis)use (Filia et al., 2021; Lambarth et al., 2023; McLaren et al., 2017), and impaired social and occupational functioning (Filia et al., 2021; Iorfino et al., 2022; Murray, Groenewald, de la Vega, & Palermo, 2020). Yet knowledge on the association between the specific characteristics of pain and how these impact young people in youth mental health settings above mental ill-health alone is limited.

Our first aim was to describe the specific characteristics of pain (frequency, intensity, and limitations) in young people attending five primary care youth mental health (*headspace)* centers in Australia for their first presentation of mental ill-health. Our second aim was to estimate the associations between pain characteristics and symptoms of depression and anxiety, suicidal ideation, social and occupational functioning, and substance use from baseline to 3-month follow-up.

## Methods

Our study was a secondary analysis of a previously reported observational study with a 3-month follow-up (Filia et al., 2021). Ethical approval was granted by the University of Melbourne Human Research Ethics Committee (ID: 1645367.1). Written informed consent was obtained from every participant as well as from their parent/guardian if they were under 18 years old. Our study is reported in line with Strengthening the Reporting of Observational studies in Epidemiology guidelines (Supplementary Table 1) (von Elm et al., 2008).

### Setting

Participants were recruited at five *headspace* centers across Australia. *headspace* is the largest Australian non-profit youth mental health network (Rickwood et al., 2018). Recruitment occurred between September 2016 and April 2018, in three metropolitan and two regional centers in different states (Australian Capital Territory, Queensland, Victoria, Tasmania) to ensure representativeness.

### Participants

All young people aged 12–25 years who attended *headspace* for a first appointment for mental health or substance use-related problems were eligible to participate.

### Data sources and measurement

At baseline, an interview was conducted, and participants completed self-report questionnaires on a tablet device. Diagnoses were based on Diagnostic and Statistical Manual for Mental Health Disorders Fourth Edition (DSM-IV) criteria and were obtained from medical records (American Psychiatric Association, 2013). After 3 months, participants were contacted by the research team to complete the follow-up assessment on-site or through an online link. Pain characteristics and clinical outcomes were assessed at both baseline and 3-month follow-up.

### Pain characteristics

Pain characteristics over the prior week were obtained from the Assessment of Quality of Life-Six dimensions (AQoL-6D) questionnaire (Allen et al., 2013; Richardson et al., 2012), which includes three questions on pain characteristics:

*Serious pain frequency:* ‘How often do you experience serious pain?’ Responses included very rarely (1), less than once a week (2), three to four times a week (3), most of the time (4).

*Pain intensity:* ‘How much pain or discomfort do you experience?’ Responses included none at all (1), I have moderate pain (2), I suffer from severe pain (3), I suffer unbearable pain (4).

*Pain limitations:* ‘How often does pain interfere with your usual activities?’ Responses included never (1), rarely (2), sometimes (3), often (4), always (5).

### Outcomes

*Symptoms of depression:* Depressive symptoms were measured using the nine-item Patient Health Questionnaire (PHQ-9) (Kroenke, Spitzer, & Williams, 2001), with total scores ranging from 0 to 27 and higher scores indicating more severe depressive symptoms.

*Symptoms of anxiety:* Anxiety symptoms were assessed using the seven-item Generalized Anxiety Disorder (GAD-7) scale (Spitzer, Kroenke, Williams, & Löwe, 2006), resulting in total scores between 0 and 21, with a higher score indicating greater symptoms of anxiety.

*Suicidal ideation:* For suicidal ideation, the 15-item Suicidal Ideation Questionnaire-Junior (SIQ-JR) was used (Reynolds, 1987). Scores ranged between 0 and 90, with higher scores indicting greater suicidal ideation.

*Social and occupational functioning:* Social and occupational functioning was assessed using the Social and Occupational Functioning Assessment Scale (SOFAS) (Goldman, Skodol, & Lave, 1992). A total score ranges from 0 to 100, with higher scores indicating greater functioning. The SOFAS is regularly used in *headspace* services to capture social, occupational, and school functioning in 12–25-year olds (Rickwood et al., 2023). This is different from pain-related activity limitations, as it captures social and occupational broadly and not just specific to pain.

*Substance use:* Substance use risk scores (tobacco, alcohol, cannabis, cocaine, amphetamine, inhalants, sedatives, hallucinogens, and opioids) were obtained from the World Health Organization Alcohol, Smoking and Substance Involvement Screening Test (WHO-ASSIST v3.0) (Humeniuk et al., 2008, 2010). Scores range from 0 to 31 for tobacco and 0 to 39 for all other substances, with higher scores indicating greater substance use risk.

### Confounders

The following variables were considered as confounders: age, sex assigned at birth (male, female), diagnosis (depression, anxiety, depression and anxiety, other), timepoint (baseline, follow-up), and the study center.

### Bias

Based on previously published studies using this data (Cotton et al., 2022; Filia et al., 2021), attrition and missing data bias were anticipated. We explored differences in baseline demographics, pain characteristics, and outcomes between participants who completed both baseline and follow-up and those who only completed baseline. In addition, restricted maximum likelihood estimations (Brauer & Curtin, 2018) and multiple imputation (Mayer, 2024) were used during analysis (see statistical methods section).

### Study size

The sample size of the current analysis was based on the available sample of the original study (*n* = 1,107 at baseline, *n* = 665 at follow-up) (Filia et al., 2021).

### Quantitative variables

All outcomes and pain characteristics were treated as continuous in analyses. Age was also treated as continuous. Covariates of sex assigned at birth, diagnosis, time, and study center were treated as categorical.

Given there were only two timepoints, we baseline-centered pain characteristics prior to multi-level modelling to evaluate whether (1) the baseline pain score (level 2 exposure; *between-participant effect*) and/or (2) change from the baseline score (level 1 exposure; *within-*participant *effect)* was associated with the outcome over time. Interpretation of the baseline-centered coefficients represent (1) if participants with higher baseline pain values had worse clinical outcomes over time relative to participants with lower baseline pain values (level 2 exposure; *between-participant effect*) and/or (2) if change from baseline pain score was associated with change in clinical outcome within each participant (level 1 exposure; *within-*participant *effect).*

### Statistical methods

All statistical analyses were conducted in R (version 4.4.1) (The Comprehensive R Archive Network, 2024). Simple tests explored differences in baseline demographics, pain characteristics, and outcomes between participants who did and did not complete follow-up assessments (Wilcoxon rank sum test; Pearson’s Chi-squared test; Fisher’s exact test). Multicollinearity for pain characteristics was checked with correlation analyses.

Multiple imputation through chained random forests was conducted prior to analysis using ‘*missRanger v2.6.0*’ (Mayer, 2024). For imputation, we specified a wide data set including demographic variables (listed in Table 1), all items (including total and standardized scores) on the AQoL6D questionnaire (Allen et al., 2013; Richardson et al., 2012), and all outcomes relevant to our analyses across both timepoints. We wide-imputed 20 datasets with a predicted mean matching score of three and converted these back to long format before subsequent analyses.

**Table 1.** Baseline demographic, pain, and outcome characteristics of the sample

Characteristic	*N*	Overall *N* = 1,107^a^	Baseline and follow-up *N* = 665^a^	Baseline only *N* = 442^a^	p-value^b^
Demographics					
Age (years)	1,107	18.1 (3.3)	18.2 (3.3)	17.9 (3.3)	0.132
Gender	1,064				0.555
Male		365 (34%)	215 (33%)	150 (36%)	
Female		660 (62%)	410 (63%)	250 (60%)	
Non-binary		39 (3.7%)	25 (3.8%)	14 (3.4%)	
Sex at birth	1,106				0.220
Female		717 (65%)	440 (66%)	277 (63%)	
Male		389 (35%)	224 (34%)	165 (37%)	
Location of center attended	1,107				0.008
Regional		400 (36%)	261 (39%)	139 (31%)	
Metro		707 (64%)	404 (61%)	303 (69%)	
Sexual orientation	1,040				0.133
Straight		748 (72%)	449 (70%)	299 (75%)	
Other		292 (28%)	190 (30%)	102 (25%)	
Primary diagnosis	1,047				0.173
Depression		186 (18%)	118 (18%)	68 (17%)	
Anxiety		271 (26%)	172 (26%)	99 (25%)	
Depression and anxiety		344 (33%)	222 (34%)	122 (31%)	
Other		246 (23%)	138 (21%)	108 (27%)	
Country of birth	1,070				0.075
Australia		961 (90%)	577 (88%)	384 (92%)	
Other		109 (10%)	75 (12%)	34 (8.1%)	
Aboriginal or Torres Strait Islander	921	40 (4.3%)	20 (3.6%)	20 (5.6%)	0.140
Currently studying	1,059				0.049
Not in education		338 (32%)	195 (30%)	143 (35%)	
Part-time student		70 (6.6%)	37 (5.7%)	33 (8.0%)	
Full-time student		651 (61%)	417 (64%)	234 (57%)	
Employment status	1,059				0.577
Unemployed		578 (55%)	347 (54%)	231 (56%)	
Paid employment		467 (44%)	290 (45%)	177 (43%)	
Unpaid employment		14 (1.3%)	10 (1.5%)	4 (1.0%)	
Current living situation	1,072				0.114
Stable		1,035 (97%)	637 (97%)	398 (95%)	
Unstable		37 (3.5%)	18 (2.7%)	19 (4.6%)	
Pain characteristics					
Serious pain frequency (1–4)	1,071				0.223
Very rarely		605 (56%)	366 (55%)	239 (58%)	
Less than once a week		289 (27%)	191 (29%)	98 (24%)	
Three to four times a week		121 (11%)	73 (11%)	48 (12%)	
Most of the time		56 (5.2%)	30 (4.5%)	26 (6.3%)	
Pain intensity (1–4)	1,071				0.479
None at all		523 (49%)	314 (48%)	209 (51%)	
I have moderate pain		475 (44%)	301 (46%)	174 (42%)	
I suffer from severe pain		64 (6.0%)	41 (6.2%)	23 (5.6%)	
I suffer unbearable pain		9 (0.8%)	4 (0.6%)	5 (1.2%)	
Pain limitations (1–5)	1,071				0.394
Never		482 (45%)	284 (43%)	198 (48%)	
Rarely		332 (31%)	212 (32%)	120 (29%)	
Sometimes		177 (17%)	117 (18%)	60 (15%)	
Often		64 (6.0%)	37 (5.6%)	27 (6.6%)	
Always		16 (1.5%)	10 (1.5%)	6 (1.5%)	
Outcomes					
Depressive symptoms (PHQ–9; 0–27)	1,068	12.8 (6.6)	12.8 (6.5)	12.8 (6.8)	0.979
Anxiety symptoms (GAD–7; 0–21)	1,067	10.4 (5.7)	10.2 (5.6)	10.6 (5.7)	0.351
Suicidal ideation (SIQ-JR; 0–90)	1,067	19.8 (20.3)	19.3 (19.9)	20.7 (21.0)	0.358
Functioning (SOFAS; 100–0)	1,072	65.2 (9.5)	65.6 (9.2)	64.6 (10.0)	0.156
Tobacco risk score (WHO-ASSIST; 0–31)	1,042	4.7 (8.3)	4.2 (7.8)	5.5 (9.1)	0.102
Alcohol risk score (WHO-ASSIST; 0–39)	1,030	6.0 (8.0)	6.1 (8.0)	5.8 (8.1)	0.356
Cannabis risk score (WHO-ASSIST; 0–39)	1,040	3.6 (8.3)	3.4 (8.0)	4.0 (8.8)	0.535

Abbreviations: PHQ-9 = Nine item Patient Health Questionnaire, GAD-7 = Seven item Generalized Anxiety Disorder Scale, SIQ-JR = Suicidal Ideation Questionnaire-Junior, SOFAS = Social and Occupational Functioning Assessment Scale, ASSIST = World Health Organization Alcohol, Smoking, and Substance Involvement Screening Test.

a Mean (SD); n (%).

b Wilcoxon rank sum test; Pearson’s Chi-squared test; Fisher’s exact test. Indicating the differences between participants with baseline and follow-up data compared to those with baseline data only.

Linear mixed effects models with restricted maximum likelihood estimations were run across both unimputed and imputed data sets using the ‘*lme4 v1.1–35.5*’ package (Bates et al., 2024). Two sets of models were used for each outcome (depressive symptoms, anxiety symptoms, suicidal ideation, functioning, and substance use) and data set (unimputed and imputed), including:

*Model 1 (unadjusted for confounders): Pain characteristics (frequency, intensity, and limitations) were included as separate characteristics (single pain variable model). All pain characteristics were included together as exposures in a combined model (multi-pain variable model).*

*Model 2 (adjusted for confounders):* Analyses were repeated with fixed confounders of age, sex assigned at birth, primary diagnosis, and timepoint.

All models included a random intercept for participants clustered within the five study centers (*three-level model*). The alpha level for p-values was set at <0.05, with p-value adjustment for the false discovery rate (FDR) (Benjamini & Hochberg, 1995), given multiple outcomes and exposures. We reported the estimates of adjusted pain characteristics from imputed data sets that were significant across both unimputed and imputed analyses after FDR adjustment of the p-values as the most robust findings.

## Results

### Descriptive data

The flow of participant selection has been previously reported (Filia et al., 2021). Baseline demographic data are presented in Table 1. The average follow-up time was 13 weeks (*SD* = 1.8). Comparisons between participants who completed follow-up assessments and those who did not showed differences only in study center location and education status (Table 1). The mean (standard deviation) age of the sample was 18.1 (3.3), with 500 (45%) participants aged between 12 and 17 years and 607 (55%) aged between 18 and 25 years.

### Pain characteristics

Pain characteristics at baseline are reported in Table 1 and follow-up in Supplementary Table 2. At baseline, 177 (16%) participants experienced serious pain more than 3 days, 51% (548) reported at least moderate pain, and 257 (25%) experienced activity limitations due to pain in the last week. Of the 346 participants with baseline and follow-up data experiencing moderate or higher pain intensity at baseline, 231 (70%) reported moderate or higher pain intensity at follow-up.

### Outcome data

Outcome data at baseline are reported in Table 1 and follow-up in Supplementary Table 2. For substance use, only tobacco, alcohol, and cannabis risk scores were analyzed, given the low proportions of participants with non-zero risk scores for cocaine, amphetamine, inhalants, sedatives, hallucinogens, and opioids (Supplementary Table 3).

### Main results

**Figure 1.**
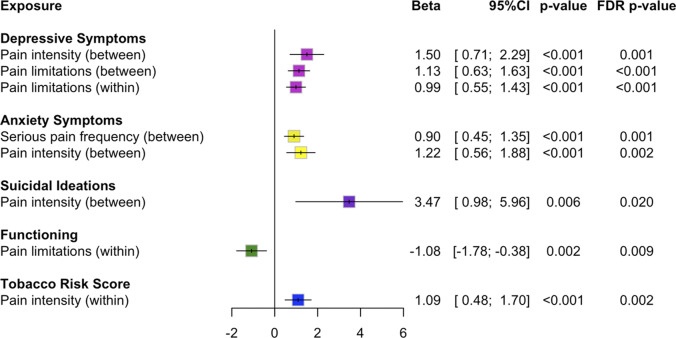
Forest plot of beta coefficients and 95% confidence intervals of pain characteristics from adjusted multi-pain variable linear mixed effects models with restricted maximum likelihood estimation. Here, we present the significant results following false discovery rate (FDR) adjustment in both datasets with and without imputation for ease of figure interpretation. Figures containing all estimates are available in Supplementary Figures 1–3. Between-participant estimates are the baseline score, indicating if baseline pain was associated with clinical outcomes across the three-month follow-up (level 2 exposure). Within‑participant estimates are baseline-centered indicating if a change from the baseline pain score was associated with a change in clinical outcome over time (level 1 exposure). Functioning refers to social and occupational functioning.

Results of correlation analyses for pain characteristics are reported in Supplementary Table 4. The correlation (r) was 0.68 between serious pain frequency and pain intensity, 0.73 between pain intensity and pain limitations, and 0.67 between serious pain frequency and pain limitations. Results of single- and multi-pain variable models are in Supplementary Tables 5 and 6, respectively, and in Figure 1 (key estimates) and Supplementary Figures 1–3 (all estimates). Full model outputs are reported in Supplementary Tables 7–34. All estimates are reported as beta coefficients and 95% confidence intervals (*β*[95%CI]).

### Depressive symptoms

*Between*-*participant effects:* Single pain variable models showed that higher baseline serious pain frequency (*β*[95%CI]: 2.21 [1.86, 2.57]; FDR-p < 0.001), pain intensity (*β*[95%CI]: 3.28 [2.79, 3.76]; FDR-p < 0.001), and pain limitations (*β*[95%CI]: 2.20 [1.88, 2.52]; FDR-p < 0.001) were associated with greater symptoms of depression over time. In multi-pain variable models, only higher pain intensity (*β*[95%CI]: 1.50 [0.71, 2.28]; FDR-p = 0.001) and pain limitations (*β*[95%CI]: 1.13 [0.63, 1.63]; FDR-p < 0.001) were associated with greater symptoms of depression.

*Within-participant effects:* Single pain variable models showed that increases in serious pain frequency (*β*[95%CI]: 1.30 [0.89, 2.57]; FDR-p < 0.001), pain intensity (*β*[95%CI]: 1.86 [1.33, 2.40]; FDR-p < 0.001), and pain limitations (*β*[95%CI]: 1.47 [1.10, 1.83]; FDR-p < 0.001) were associated with increases in symptoms of depression. Only increases in pain limitations (*β*[95%CI]: 0.99 [0.54, 1.43]; FDR-p < 0.001) were associated with increases in symptoms of depression in multi-pain variable models.

### Anxiety symptoms

*Between-participant effects:* Higher serious pain frequency (*β*[95%CI]: 1.91 [1.61, 2.21]; FDR-p < 0.001), pain intensity (*β*[95%CI]: 2.60 [2.19, 3.00]; FDR-p < 0.001), and pain limitations (*β*[95%CI]: 1.62 [1.35, 1.89]; FDR-p < 0.001) were associated with greater symptoms of anxiety over time in single pain variable models. Higher serious pain frequency (*β*[95%CI]: 0.90 [0.45, 1.35]; FDR-p = 0.001) and pain intensity (*β*[95%CI]: 1.22 [0.56, 1.89]; FDR-p = 0.002) were associated with greater anxiety symptoms in both multi-pain variable models.

*Within-participant effects:* Single pain variable models showed that increases in serious pain frequency (*β*[95%CI]: 1.25 [0.90, 1.61]; FDR-p < 0.001), pain intensity (*β*[95%CI]: 1.58 [1.12, 2.05]; FDR-p < 0.001), and pain limitations (*β*[95%CI]: 1.23 [0.91, 1.55]; FDR-p < 0.001) were associated with increases in symptoms of anxiety. No associations were observed in multi-pain variable models.

### Suicidal ideation

*Between-participant effects:* Single pain variable models indicated that greater serious pain frequency (*β*[95%CI]: 5.58 [4.45, 6.71]; FDR-p < 0.001), pain intensity (*β*[95%CI]: 8.05 [6.52, 9.58]; FDR-p < 0.001), and pain limitations (*β*[95%CI]: 5.39 [4.38, 6.39]; FDR-p < 0.001) were associated with suicidal ideation over time. Only higher pain intensity (*β*[95%CI]: 3.47 [0.98, 5.96]; FDR-p = 0.020) was associated with greater suicidal ideation in multi-pain variable models.

*Within-participant effects:* Increases in serious pain frequency (*β*[95%CI]: 2.71 [1.56, 3.85]; FDR-p < 0.001), pain intensity (*β*[95%CI]: 4.27 [2.76, 5.78]; FDR-p < 0.001), and pain limitations (*β*[95%CI]: 3.08 [2.04, 4.12]; FDR-p < 0.001) were associated with increases in suicidal ideation in single pain variable models. No within-participant effects were observed in multi-pain variable models.

### Social and occupational functioning

*Between-participant effects:* Single pain variable models showed that higher serious pain frequency (*β*[95%CI]: ‑1.60 [−2.17, −1.02]; FDR-p < 0.001), pain intensity (*β*[95%CI]: ‑2.64 [−3.42, −1.87]; FDR-p < 0.001), and pain limitations (*β*[95%CI]: −1.77 [−2.27, −1.26]; FDR-p < 0.001) were associated with lower social and occupational functioning over time. No associations between pain characteristics and social and occupational functioning were observed in multi‑pain variable models.

*Within-participant effects:* Results from single pain variable models showed that increases in serious pain frequency (*β*[95%CI]: ‑1.19 [−1.82, −0.55]; FDR-p < 0.001), pain intensity (*β*[95%CI]: ‑1.81 [−2.65, −0.97]; FDR-p < 0.001), and pain limitations (*β*[95%CI]: −1.49 [−2.06, −0.91]; FDR-p < 0.001) were associated with decreases in social and occupational functioning. In multi-pain variable models, only increases in pain limitations (*β*[95%CI]: −1.08 [−1.78, −0.38]; FDR-p = 0.009) were associated with decreases in social and occupational functioning.

### Tobacco use risk scores

*Between-participant effects:* Results from single pain variable models showed that greater serious pain frequency (*β*[95%CI]: 1.23 [0.75, 1.71]; FDR-p < 0.001), pain intensity (*β*[95%CI]: 1.51 [0.85, 2.16]; FDR-p < 0.001), and pain limitations (*β*[95%CI]: 1.06 [0.63, 1.48]; FDR-p < 0.001) were associated with higher tobacco use risk scores over time. No associations were observed with tobacco use risk scores in multi-pain variable models.

*Within-participant effects:* Only increases in pain intensity (*β*[95%CI]: 0.96 [0.45, 1.46]; FDR-p = 0.001) was associated with increases in tobacco use risk scores in single pain variable models. Increases in pain intensity (*β*[95%CI]: 1.09 [0.48, 1.70]; FDR-p = 0.002) were also associated with increases in tobacco use risk scores in multi-pain variable models.

### Alcohol use risk scores

No effects of pain characteristics in both single- and multi-pain variable models were observed for both between- and within-effects for alcohol use risk.

### Cannabis use risk scores

*Between-participant effects*: Only higher serious pain frequency (*β*[95%CI]: 1.26 [0.77, 1.75]; FDR-p < 0.001) was associated with greater cannabis use risk scores across single pain variable models over time. No effects of pain characteristics were observed on cannabis use risk scores in multi-pain variable models.

*Within-participant effects:* No effects of pain characteristics in both single- and multi-pain variable models were observed for cannabis use risk scores.

## Discussion

Mental ill-health and pain conditions can co-occur in young people (Cotton et al., 2022), yet the characteristics of this pain and the impact on clinical outcomes in the early phases of treatment have remained unexplored. Our findings showed that one-in-six young people accessing early intervention-focused mental healthcare experienced serious pain more than 3 days, one-in-two reported at least moderate pain, and one-in-four reported limitations to their usual activities due to pain in the last week. Furthermore, 70% of participants who reported baseline pain also reported pain at follow-up and may represent a group experiencing chronic pain (Treede et al., 2019). Findings showed that those with higher baseline pain values had worse clinical outcomes over time compared to those with lower baseline pain values. Specifically, higher serious pain frequency was associated with greater symptoms of anxiety, higher pain intensity was associated with greater symptoms of depression, anxiety, and suicidal ideation, and higher pain limitations were associated with greater depressive symptoms. Furthermore, an increase in pain intensity associated with an increase in tobacco use risk scores, while increases in pain limitations were associated with increases in depressive symptoms and decreases social and occupational functioning. Our findings showed the substantial negative impact that specific pain characteristics have on young people with mental ill-health and indicate that youth mental health services should screen for pain at intake. Improved and timely assessment must be complemented with collaborative models of pain care in mental health settings (Patel et al., 2023), to improve clinical outcomes for young people with mental ill-health.

Our results showed that 51% of young people care seeking for mental ill-health report pain. In contrast, one study showed a chronic pain prevalence of ~70% in adolescents with psychiatric disorders (Mangerud, Bjerkeset, Lydersen, & Indredavik, 2013); however, this was in a tertiary care sample. Participants may have had more severe presentations compared to our sample, which was recruited from primary care settings. Another difference is that our study did not examine chronic pain specifically. In adults with mental ill-health, studies have reported a prevalence of pain (combined acute and chronic) ranging from 42% to 65% (Bair, Robinson, Katon, & Kroenke, 2003; Kroenke et al., 2008). The reasons for this co-occurrence remain poorly understood; however, neurobiological (brain alterations [Bair et al., 2003; Hooten, 2016] and neuroimmune [Campos et al., 2020; Walker et al., 2014]), and behavioral, psychological, and environmental (Khan, Michelini, & Battaglia, 2020) mechanisms have been proposed. For example, pain conditions are associated with areas of brain processing related to emotion (Martucci & Mackey, 2018), which overlap with those linked to mental ill-health in young people (Soltani, Kopala-Sibley, & Noel, 2019). Furthermore, persistent neuroinflammation has been observed to influence the onset and persistence of both mental ill-health and pain (Campos et al., 2020). Finally, various behavioral (e.g. sleep quality), psychological (e.g. affect, rumination), and environmental factors (e.g. particularly parental and familial factors in young people) are known to influence both mental ill‑health and pain (Soltani et al., 2019). Our findings showed that the prevalence of pain in young people with mental ill‑health accentuates the urgent need to understand and treat this co-occurrence as early as possible to prevent it from continuing into adulthood, where there is the potential for continued functional and social limitations (De La Rosa et al., 2024). It emphasizes the need for researchers to better understand shared mechanisms to optimize treatment decisions.

Higher baseline pain intensity and limitations were found to be associated with greater depressive symptoms over time. These findings are similar to those of a population-based study on Australian young people (Kamper et al., 2019), which found that higher pain frequency was associated with poor mental health. Other studies in young people experiencing pain have shown comparable results (Hu et al., 2022; Zvolensky et al., 2020). Yet here we have demonstrated the importance of co-occurring pain in young people with mental ill-health. Depressive symptoms could be worsened by pain due to factors such as less sleep, stress, lower self-efficacy, hopelessness, lower functioning, or limited social participation and positive reinforcement; however, this relationship is likely bidirectional (Hazeldine-Baker, Salkovskis, Osborn, & Gauntlett-Gilbert, 2018; Thompson et al., 2019; Wise et al., 2008). Pain intensity and limitations contribute to these factors, potentially explaining the association between these specific characteristics and depressive symptoms in the current study. A recent systematic review (Liu et al., 2024) showed effect sizes for treatments of depressive symptoms in adults with depression are smaller in those experiencing pain compared to those without pain. Therefore, screening for the possible presence of pain and optimizing pain management in patients with depressive symptoms could improve treatment efficacy.

Greater pain intensity and serious pain frequency at baseline were associated with higher symptoms of anxiety. Pain has been linked to symptoms of anxiety in other studies (Blaauw et al., 2014; Hommer, Lateef, He, & Merikangas, 2022; Kamper et al., 2019), but our study is the first to examine this relationship in young people experiencing mental ill-health. The fear-avoidance model (Lethem, Slade, Troup, & Bentley, 1983) may be used to explain the relationship between pain and anxiety suggesting that these conditions not only often co-occur but also exacerbate each other (Hooten, 2016; Vlaeyen & Linton, 2000). When pain is perceived as catastrophic, it can lead to intense fear and avoidance of physical activity, ultimately resulting in more pain (Vlaeyen & Linton, 2000). Strategies to manage increased pain episodes should be used to support anxiety in young people with mental ill-health.

Higher baseline pain intensity and limitations were associated with higher levels of suicidal ideation in our study. These associations could be explained by disability, hopelessness, or a desire to escape from pain (Fishbain et al., 2012; Tang & Crane, 2006). This is in line with prior research in both mental ill-health and chronic pain populations (Hinze et al., 2019, 2023; Kowal, Wilson, Henderson, & McWilliams, 2014; Wildeboer, Chambers, Soltani, & Noel, 2023). For co-occurring mental ill-health and pain, one prior study showed that higher levels of depressive symptoms were associated with suicidality onset in adolescents with chronic pain (Wildeboer et al., 2023). A systematic review also showed that depression moderated suicidal ideation in young people experiencing pain; however, it did not fully explain the pain-suicidality relationship (Hinze et al., 2019). These together support the findings of our study and may indicate that, regardless of service (e.g. mental health or pain), clinicians should consider the impact of co-occurring mental ill-health and pain symptoms on suicidal ideation.

Increases in the experience of pain-related limitations were associated with decreases in social and occupational functioning. This could indicate that young people are able to develop coping mechanisms that mitigate the impact of their pain in social and occupational settings. However, if the young person deviates from their usual level of pain limitations, this has the potential to negatively influence their social and occupational functioning. Previous studies have shown that children and adolescents with pain experience more restrictions in daily functioning, such as in school, hobbies, and social activities. (Cohen, Vowles, & Eccleston, 2010; Kaczynski, Claar, & LeBel, 2013; Logan, Simons, Stein, & Chastain, 2008; Roth-Isigkeit et al., 2005). This is further supported by other studies in young people with chronic pain (Bateman et al., 2023; Serbic, Friedrich, & Murray, 2023; van Alboom et al., 2022) and mental ill-health (Filia et al., 2021; Iorfino et al., 2022). Our results show the distinct impact of pain on social and occupational functioning, over and above the burden of mental ill-health alone. These results suggest that clinicians should monitor increases in activity limitations due to pain, as this may negatively affect social and occupational functioning in young people with mental health conditions.

Our study showed that increases in pain intensity were associated with increases in tobacco use risk. This indicates that an increase in pain intensity may result in an increased risk of problematic tobacco use. Studies have found pain intensity to be associated with smoking in both adolescents (Kamper et al., 2019) and adults (Barry, Pilver, Hoff, & Potenza, 2013; Ditre, Brandon, Zale, & Meagher, 2011). This association could be explained by the analgesic effect of nicotine (Kishioka, Kiguchi, Kobayashi, & Saika, 2014). However, over the longer term, smoking and pain can exacerbate each other through a positive feedback loop (Ditre & Brandon, 2008). No significant associations were found for alcohol and cannabis use. This emphasizes the importance of screening for fluctuations in pain intensity and providing support to quit smoking to prevent longer-term negative changes, given the association between pain intensity and tobacco use.

The current study has multiple strengths. A large longitudinal dataset was used; participants represented a broad age range and were from diverse locations across Australia, including measures which are commonly used and well-validated (Allen et al., 2013; Goldman et al., 1992; Humeniuk et al., 2008, 2010; Kroenke et al., 2001; Reynolds, 1987; Richardson et al., 2012; Spitzer et al., 2006). Participants had a first presentation of mental ill-health, reflecting the prevalence and influence of pain characteristics on clinical outcomes in youth with mental ill-health during the early treatment stages (first 3 months). We explored both between- and within-participant effects in multi-level mixed models and the potential impact of attrition on effect estimates through comparison of demographic, pain characteristics, and outcome data, restricted maximum likelihood estimations, and multiple imputation. Furthermore, we adjusted p-values for the FDR to account for the testing of multiple exposures and outcomes to reduce the potential of false positive results (Benjamini & Hochberg, 1995).

In terms of limitations, only 62% of recruited participants completed both baseline and follow-up assessments. There was no available information on the location or type of pain, underlying medical conditions that could indicate secondary pain conditions (rather than primary), duration of pain (acute or chronic), or if pain was assessed in standard intake procedures. This could have been insightful, since multisite and/or chronic pain is likely to have a larger influence on clinical outcomes (Mangerud et al., 2013) and should be considered in future research. Given that our study only had two timepoints, future research could validate these findings in more intensive longitudinal designs to explore how pain and mental ill-health co-fluctuate and to further understand their individual and combined effects on treatment engagement and outcomes.

Our study emphasizes the need for clinicians and researchers to consider the co-occurrence between mental ill-health and pain. It would be prudent for clinicians in youth mental health settings to screen for pain at intake, work with the young person to determine real-world impacts of co-occurring pain on their mental health and functioning, and provide advice or referrals to integrated treatment as appropriate. For researchers, there is a knowledge gap regarding youth experiences of co-occurring mental ill-health and pain (Soltani et al., 2019); there have been no trials that have recruited young people experiencing both mental ill-health and pain and provided targeted, integrated, and accessible treatments to this population (Ma et al., 2024). The impact of co-occurring mental ill-health and pain can continue into adulthood in a reinforcing negative cycle that can then lead to occupational and relationship problems (De La Rosa et al., 2024). Similar to mental health conditions (Solmi et al., 2022), pain develops in early adolescence (Chambers et al., 2024) and can lead to mental ill-health (Bondesson, Bolmsjö, Pardo, & Jöud, 2024). There is an urgent need to recognize the emergence of pain in adolescence and promote efforts for early intervention and prevention to reduce the burden of pain and its mental health impacts, as well as evaluate this in longer-term follow-up studies. Overall, these results indicate there is an urgent need for researchers to answer key gaps in our understanding and treatments for young people with co-occurring mental ill-health and pain.

## Conclusion

We explored the prevalence and impact of pain in young people with mental ill-health accessing early intervention primary mental healthcare services. Results showed that one-in-two young people with mental ill-health also report pain at intake. Serious pain frequency, intensity, and limitations were found to have a negative impact on symptoms of depression, anxiety, suicidal ideation, social and occupational functioning, and tobacco use for young people in the early treatment stages of mental ill-health. These results highlight the need for early pain recognition in mental health settings to support the one-in-two young people with mental ill-health who report pain. Developing more integrated and collaborative care strategies is paramount to reduce the major burden of this co-occurrence among young people and mitigate potential negative longer-term outcomes into adulthood.

## Supporting information

Oosterwijk et al. supplementary material 1 Oosterwijk et al. supplementary material

Oosterwijk et al. supplementary material 2 Oosterwijk et al. supplementary material

## Data Availability

Due to ethical considerations for this study, the code and dataset used in the current study are available from the corresponding author on reasonable request.

## Supplementary material

The supplementary material for this article can be found at http://doi.org/10.1017/S0033291725101104.

## References

[r1] Allen, J., Inder, K. J., Lewin, T. J., Attia, J. R., & Kelly, B. J. (2013). Construct validity of the assessment of quality of life – 6D (AQoL-6D) in community samples. Health and Quality of Life Outcomes, 11(1), 61–75. 10.1186/1477-7525-11-61. 23590808 PMC3639231

[r2] American Psychiatric Association. (2013). Diagnostic and statistical manual of mental disorders (5th ed.). American Psychiatric Publishing. 10.1176/appi.books.9780890425596.

[r3] Bair, M. J., Robinson, R. L., Katon, W., & Kroenke, K. (2003). Depression and pain comorbidity: A literature review. Archives of Internal Medicine, 163(20), 2433. 10.1001/archinte.163.20.2433. 14609780

[r4] Barry, D. T., Pilver, C. E., Hoff, R. A., & Potenza, M. N. (2013). Pain interference and incident mood, anxiety, and substance-use disorders: Findings from a representative sample of men and women in the general population. Journal of Psychiatric Research, 47(11), 1658–1664. 10.1016/j.jpsychires.2013.08.004. 23992771 PMC3835154

[r5] Bateman, S., Caes, L., Eccleston, C., Noel, M., & Jordan, A. (2023). Co‐occurring chronic pain and primary psychological disorders in adolescents: A scoping review. Paediatric and Neonatal Pain, 5(3), 57–65. 10.1002/pne2.12107. 37744281 PMC10514777

[r6] Bates, D., Maechler, M., Bolker, B., Walker, S., Singmann, H., Dai, B., Scheipl, F., Grothendieck, G., Green, P., Fox, J., Bauer, A., Krivitsky, P. N., Tanaka, E., & Jagan, M. (2024). lme4: Linear mixed-effects models using “Eigen” and S4 [Computer software]. https://cran.r-project.org/web/packages/lme4/index.html

[r7] Benjamini, Y., & Hochberg, Y. (1995). Controlling the false discovery rate: A practical and powerful approach to multiple testing. Journal of the Royal Statistical Society: Series B (Methodological), 57(1), 289–300. 10.1111/j.2517-6161.1995.tb02031.x.

[r8] Blaauw, B. A., Dyb, G., Hagen, K., Holmen, T. L., Linde, M., Wentzel-Larsen, T., & Zwart, J.-A. (2014). Anxiety, depression and behavioral problems among adolescents with recurrent headache: The young-HUNT study. The Journal of Headache and Pain, 15(1), 38. 10.1186/1129-2377-15-38. 24925252 PMC4062897

[r9] Bondesson, E., Bolmsjö, B. B., Pardo, F. L., & Jöud, A. S. (2024). Temporal relationship between pain and mental health conditions among children and young people – A population-based register study in Sweden. The Journal of Pain, 25(12), 104662. 10.1016/j.jpain.2024.104662. 39209085

[r10] Bowman, S., McKinstry, C., & McGorry, P. (2017). Youth mental ill health and secondary school completion in Australia: Time to act. Early Intervention in Psychiatry, 11(4), 277–289. 10.1111/eip.12357. 27381567

[r11] Brauer, M., & Curtin, J. J. (2018). Linear mixed-effects models and the analysis of nonindependent data: A unified framework to analyze categorical and continuous independent variables that vary within-subjects and/or within-items. Psychological Methods, 23(3), 389–411. 10.1037/met0000159. 29172609

[r12] Campos, A. C. P., Antunes, G. F., Matsumoto, M., Pagano, R. L., & Martinez, R. C. R. (2020). Neuroinflammation, pain and depression: An overview of the Main findings. Frontiers in Psychology, 11. 10.3389/fpsyg.2020.01825. PMC7412934 32849076

[r13] Chambers, C. T., Dol, J., Tutelman, P. R., Langley, C. L., Parker, J. A., Cormier, B. T., Macfarlane, G. J., Jones, G. T., Chapman, D., Proudfoot, N., Grant, A., & Marianayagam, J. (2024). The prevalence of chronic pain in children and adolescents: A systematic review update and meta-analysis. Pain, 165(10), 2215–2234. 10.1097/j.pain.0000000000003267 38743558 PMC11404345

[r14] Cohen, L. L., Vowles, K. E., & Eccleston, C. (2010). The impact of adolescent chronic pain on functioning: Disentangling the complex role of anxiety. The Journal of Pain, 11(11), 1039–1046. 10.1016/j.jpain.2009.09.009. 20015706

[r15] Cohen, S. P., Vase, L., & Hooten, W. M. (2021). Chronic pain: An update on burden, best practices, and new advances. The Lancet, 397(10289), 2082–2097. 10.1016/S0140-6736(21)00393-7. 34062143

[r16] Cotton, S. M., Hamilton, M. P., Filia, K., Menssink, J. M., Engel, L., Mihalopoulos, C., Rickwood, D., Hetrick, S. E., Parker, A. G., Herrman, H., Telford, N., Hickie, I., McGorry, P. D., & Gao, C. X. (2022). Heterogeneity of quality of life in young people attending primary mental health services. Epidemiology and Psychiatric Sciences, 31, e55. 10.1017/S2045796022000427. 35856272 PMC9305730

[r17] De La Rosa, J. S., Brady, B. R., Ibrahim, M. M., Herder, K. E., Wallace, J. S., Padilla, A. R., & Vanderah, T. W. (2024). Co-occurrence of chronic pain and anxiety/depression symptoms in U.S. adults: Prevalence, functional impacts, and opportunities. Pain, 165(3), 666–673. 10.1097/j.pain.0000000000003056. 37733475 PMC10859853

[r18] Ditre, J. W., & Brandon, T. H. (2008). Pain as a motivator of smoking: Effects of pain induction on smoking urge and behavior. Journal of Abnormal Psychology, 117(2), 467–472. 10.1037/0021-843X.117.2.467. 18489224 PMC4391507

[r19] Ditre, J. W., Brandon, T. H., Zale, E. L., & Meagher, M. M. (2011). Pain, nicotine, and smoking: Research findings and mechanistic considerations. Psychological Bulletin, 137(6), 1065–1093. 10.1037/a0025544. 21967450 PMC3202023

[r20] Dudeney, J., Aaron, R. V., Hathway, T., Bhattiprolu, K., Bisby, M. A., McGill, L. S., Gandy, M., Harte, N., & Dear, B. F. (2024). Anxiety and depression in youth with chronic pain: A systematic review and meta-analysis. JAMA Pediatrics, 178(11), 1114–1123. 10.1001/jamapediatrics.2024.3039. 39250143 PMC11385330

[r21] Filia, K., Rickwood, D., Menssink, J., Gao, C. X., Hetrick, S., Parker, A., Hamilton, M., Hickie, I., Herrman, H., Telford, N., Sharmin, S., McGorry, P., & Cotton, S. (2021). Clinical and functional characteristics of a subsample of young people presenting for primary mental healthcare at headspace services across Australia | social psychiatry and psychiatric epidemiology. Social Psychiatry and Psychiatric Epidemiology, 56, 1311–1323. 33452888 10.1007/s00127-020-02020-6

[r22] Fishbain, D. A., Bruns, D., Meyer, L. J., Lewis, J. E., Gao, J., & Disorbio, J. M. (2012). Exploration of the relationship between disability perception, preference for death over disability, and suicidality in patients with acute and chronic pain. Pain Medicine, 13(4), 552–561. 10.1111/j.1526-4637.2012.01358.x. 22487542

[r23] Fisher, E., Monsell, F., Clinch, J., & Eccleston, C. (2024). Who develops chronic pain after an acute lower limb injury? A longitudinal study of children and adolescents. Pain, 165(11), 2507. 10.1097/j.pain.0000000000003274. 38842496 PMC7616524

[r24] Friedrichsdorf, S. J., Postier, A., Eull, D., Weidner, C., Foster, L., Gilbert, M., & Campbell, F. (2015). Pain outcomes in a US children’s hospital: A prospective Cross-sectional survey. Hospital Pediatrics, 5(1), 18–26. 10.1542/hpeds.2014-0084. 25554755

[r25] Gibb, S. J., Fergusson, D. M., & Horwood, L. J. (2010). Burden of psychiatric disorder in young adulthood and life outcomes at age 30. The British Journal of Psychiatry, 197(2), 122–127. 10.1192/bjp.bp.109.076570. 20679264

[r26] Goldman, H. H., Skodol, A. E., & Lave, T. R. (1992). Revising axis V for DSM-IV: A review of measures of social functioning. American Journal of Psychiatry, 149(9), 1148–1156. 10.1176/ajp.149.9.1148. 1386964

[r27] Hassett, A. L., Hilliard, P. E., Goesling, J., Clauw, D. J., Harte, S. E., & Brummett, C. M. (2013). Reports of chronic pain in childhood and adolescence among patients at a tertiary care pain clinic. The Journal of Pain, 14(11), 1390–1397. 10.1016/j.jpain.2013.06.010. 24021576

[r28] Hazeldine-Baker, C. E., Salkovskis, P. M., Osborn, M., & Gauntlett-Gilbert, J. (2018). Understanding the link between feelings of mental defeat, self-efficacy and the experience of chronic pain. British Journal of Pain, 12(2), 87–94. 10.1177/2049463718759131. 29796260 PMC5958515

[r29] Hinze, V., Crane, C., Ford, T., Buivydaite, R., Qiu, L., & Gjelsvik, B. (2019). The relationship between pain and suicidal vulnerability in adolescence: A systematic review. The Lancet Child & Adolescent Health, 3(12), 899–916. 10.1016/S2352-4642(19)30267-6. 31606322 PMC6842327

[r30] Hinze, V., Ford, T., Crane, C., Haslbeck, J. M. B., Hawton, K., Gjelsvik, B., Allwood, M., Aukland, L., Casey, T., De Wilde, K., Farley, E.-R., Fletcher, K., Kappelmann, N., Kuyken, W., Laws, S., Lord, L., Medlicott, E., Montero-Marin, J., Nuthall, E., … Wainman, B. (2021). Does depression moderate the relationship between pain and suicidality in adolescence? A moderated network analysis. Journal of Affective Disorders, 292, 667–677. 10.1016/j.jad.2021.05.100 34157662 PMC8323496

[r31] Hinze, V., Karl, A., Ford, T., & Gjelsvik, B. (2023). Pain and suicidality in children and adolescents: A longitudinal population-based study. European Child & Adolescent Psychiatry, 32(8), 1507–1517. 10.1007/s00787-022-01963-2. 35235043 PMC10326152

[r32] Hirsch, M., Dhillon-Smith, R., Cutner, A. S., Yap, M., & Creighton, S. M. (2020). The prevalence of endometriosis in adolescents with pelvic pain: A systematic review. Journal of Pediatric and Adolescent Gynecology, 33(6), 623–630. 10.1016/j.jpag.2020.07.011. 32736134

[r33] Holley, A. L., Wilson, A. C., & Palermo, T. M. (2017). Predictors of the transition from acute to persistent musculoskeletal pain in children and adolescents: A prospective study. Pain, 158(5), 794–801. 10.1097/j.pain.0000000000000817. 28151835 PMC5393939

[r34] Hommer, R., Lateef, T., He, J.-P., & Merikangas, K. (2022). Headache and mental disorders in a nationally representative sample of American youth. European Child & Adolescent Psychiatry, 31(1), 39–49. 10.1007/s00787-020-01599-0. 33721086 PMC8691207

[r35] Hooten, W. M. (2016). Chronic pain and mental health disorders. Mayo Clinic Proceedings, 91(7), 955–970. 10.1016/j.mayocp.2016.04.029. 27344405

[r36] Hu, L., Liu, Z.-Z., Wang, Z.-Y., Jia, C.-X., & Liu, X. (2022). Associations between pain and depressive symptoms: A longitudinal study of Chinese adolescents. Journal of Affective Disorders, 299, 675–681. 10.1016/j.jad.2021.12.095. 34953924

[r37] Huguet, A., & Miró, J. (2008). The severity of chronic pediatric pain: An epidemiological study. The Journal of Pain, 9(3), 226–236. 10.1016/j.jpain.2007.10.015. 18088558

[r38] Humeniuk, R., Ali, R., Babor, T. F., Farrell, M., Formigoni, M. L., Jittiwutikarn, J., De Lacerda, R. B., Ling, W., Marsden, J., Monteiro, M., Nhiwatiwa, S., Pal, H., Poznyak, V., & Simon, S. (2008). Validation of the alcohol, smoking and substance involvement screening test (ASSIST). Addiction, 103(6), 1039–1047. 10.1111/j.1360-0443.2007.02114.x. 18373724

[r39] Humeniuk, R.E., Henry-Edwards, S., Ali, R.L., Poznyak, V., and Monteiro, M. (2010). The Alcohol, Smoking and Substance Involvement Screening Test (ASSIST): manual for use in primary care. Geneva, World Health Organization.

[r40] Hunfeld, J. A. M., Perquin, C. W., Duivenvoorden, H. J., Hazebroek-Kampschreur, A. A. J. M., Passchier, J., Van Suijlekom-Smit, L. W. A., & Van der Wouden, J. C. (2001). Chronic pain and its impact on quality of life in adolescents and their families. Journal of Pediatric Psychology, 26(3), 145–153. 11259516 10.1093/jpepsy/26.3.145

[r41] Iorfino, F., Carpenter, J. S., Cross, S. P., Crouse, J., Davenport, T. A., Hermens, D. F., Yee, H., Nichles, A., Zmicerevska, N., Guastella, A., Scott, E. M., & Hickie, I. B. (2022). Social and occupational outcomes for young people who attend early intervention mental health services: A longitudinal study. Medical Journal of Australia, 216(2), 87–93. https://www.mja.com.au/journal/2022/216/2/social-and-occupational-outcomes-young-people-who-attend-early-intervention 34664282 10.5694/mja2.51308

[r42] Jaycox, L. H., Stein, B. D., Paddock, S., Miles, J. N. V., Chandra, A., Meredith, L. S., Tanielian, T., Hickey, S., & Burnam, M. A. (2009). Impact of teen depression on academic, social, and physical functioning. Pediatrics, 124(4), e596–e605. 10.1542/peds.2008-3348. 19736259

[r43] Kaczynski, K. J., Claar, R. L., & LeBel, A. A. (2013). Relations between pain characteristics, child and parent variables, and school functioning in adolescents with chronic headache: A comparison of tension-type headache and migraine. Journal of Pediatric Psychology, 38(4), 351–364. 10.1093/jpepsy/jss120. 23248346

[r44] Kamper, S. J., Michaleff, Z. A., Campbell, P., Dunn, K. M., Yamato, T. P., Hodder, R. K., Wiggers, J., & Williams, C. M. (2019). Back pain, mental health and substance use are associated in adolescents. Journal of Public Health, 41(3), 487–493. 10.1093/pubmed/fdy129. 30204888

[r45] Kessler, R. C., Berglund, P., Demler, O., Jin, R., Merikangas, K. R., & Walters, E. E. (2005). Lifetime prevalence and age-of-onset distributions of DSM-IV disorders in the national comorbidity survey replication. Archives of General Psychiatry, 62(6), 593–602. 10.1001/archpsyc.62.6.593. 15939837

[r46] Khan, W. U., Michelini, G., & Battaglia, M. (2020). Twin studies of the covariation of pain with depression and anxiety: A systematic review and re-evaluation of critical needs. Neuroscience & Biobehavioral Reviews, 111, 135–148. 10.1016/j.neubiorev.2020.01.015. 31954722

[r47] Kishioka, S., Kiguchi, N., Kobayashi, Y., & Saika, F. (2014). Nicotine effects and the endogenous opioid system. Journal of Pharmacological Sciences, 125(2), 117–124. 10.1254/jphs.14r03cp. 24882143

[r48] Kowal, J., Wilson, K. G., Henderson, P. R., & McWilliams, L. A. (2014). Change in suicidal ideation after interdisciplinary treatment of chronic pain. The Clinical Journal of Pain, 30(6), 463–471. 10.1097/AJP.0000000000000003. 24281291 PMC4014432

[r49] Kroenke, K., Shen, J., Oxman, T. E., Williams, J. W., & Dietrich, A. J. (2008). Impact of pain on the outcomes of depression treatment: Results from the RESPECT trial. Pain, 134(1), 209–215. 10.1016/j.pain.2007.09.021. 18022319

[r50] Kroenke, K., Spitzer, R. L., & Williams, J. B. W. (2001). The PHQ-9. Journal of General Internal Medicine, 16(9), 606–613. 10.1046/j.1525-1497.2001.016009606.x. 11556941 PMC1495268

[r51] Lambarth, A., Katsoulis, M., Ju, C., Warwick, A., Takhar, R., Dale, C., Prieto-Merino, D., Morris, A., Sen, D., Wei, L., & Sofat, R. (2023). Prevalence of chronic pain or analgesic use in children and young people and its long-term impact on substance misuse, mental illness, and prescription opioid use: A retrospective longitudinal cohort study. The Lancet Regional Health – Europe, 35. 10.1016/j.lanepe.2023.100763. PMC10730316 38115960

[r52] Lethem, J., Slade, P. D., Troup, J. D. G., & Bentley, G. (1983). Outline of a fear-avoidance model of exaggerated pain perception – I. Behaviour Research and Therapy, 21(4), 401–408. 10.1016/0005-7967(83)90009-8. 6626110

[r53] Liu, J. J., Huang, X., Bao, Y.-P., Lu, L., Dong, P., Wolkowitz, O. M., Kelsoe, J. R., Shi, J., & Wei, Y. B. (2024). Painful physical symptoms and antidepressant treatment outcome in depression: A systematic review and meta-analysis. Molecular Psychiatry, 29, 2560–2567. 10.1038/s41380-024-02496-7. 38480874

[r54] Logan, D. E., Simons, L. E., Stein, M. J., & Chastain, L. (2008). School impairment in adolescents with chronic pain. The Journal of Pain, 9(5), 407–416. 10.1016/j.jpain.2007.12.003. 18255341

[r55] Ma, R., Romano, E., Ashworth, M., Smith, T. O., Vancampfort, D., Scott, W., Gaughran, F., Stewart, R., & Stubbs, B. (2024). The effectiveness of interventions for improving chronic pain symptoms among people with mental illness: A systematic review. The Journal of Pain, 25(5), 104421. 10.1016/j.jpain.2023.11.004. 37952860

[r56] Mangerud, W. L., Bjerkeset, O., Lydersen, S., & Indredavik, M. S. (2013). Chronic pain and pain-related disability across psychiatric disorders in a clinical adolescent sample. BMC Psychiatry, 13(1), 272. 10.1186/1471-244X-13-272. 24139217 PMC3853574

[r57] Martucci, K. T., & Mackey, S. C. (2018). Neuroimaging of pain: Human evidence and clinical relevance of central nervous system processes and modulation. Anesthesiology, 128(6), 1241. 10.1097/ALN.0000000000002137. 29494401 PMC5953782

[r58] Mayer, M. (2024). *missRanger: Fast imputation of missing values* (version 2.6.0) [Computer software]. https://cran.r-project.org/web/packages/missRanger/index.html

[r59] McGorry, P. D., Mei, C., Dalal, N., Alvarez-Jimenez, M., Blakemore, S.-J., Browne, V., Dooley, B., Hickie, I. B., Jones, P. B., McDaid, D., Mihalopoulos, C., Wood, S. J., Azzouzi, F. A. E., Fazio, J., Gow, E., Hanjabam, S., Hayes, A., Morris, A., Pang, E., … Killackey, E. (2024). The lancet psychiatry commission on youth mental health. The Lancet Psychiatry, 11(9), 731–774. 10.1016/S2215-0366(24)00163-9. 39147461

[r60] McLaren, N., Kamper, S. J., Hodder, R., Wiggers, J., Wolfenden, L., Bowman, J., Campbell, E., Dray, J., & Williams, C. M. (2017). Increased substance use and poorer mental health in adolescents with problematic musculoskeletal pain. Journal of Orthopaedic & Sports Physical Therapy, 47(10), 705–711. 10.2519/jospt.2017.7441. 28967339

[r61] Moller, C., Davey, C., Badcock, P., Wrobel, A., Cao, A., Murrihy, S., Sharmin, S., & Cotton, S. (2022). Correlates of suicidality in young people with depressive disorders: A systematic review. The Australian and New Zealand Journal of Psychiatry, 56(8), 910–948. 10.1177/00048674221086498. 35362327

[r62] Murray, C. B., Groenewald, C. B., de la Vega, R., & Palermo, T. M. (2020). Long-term impact of adolescent chronic pain on young adult educational, vocational, and social outcomes. Pain, 161(2), 439–445. 10.1097/j.pain.0000000000001732. 31651579 PMC7001863

[r63] Patel, V., Saxena, S., Lund, C., Kohrt, B., Kieling, C., Sunkel, C., Kola, L., Chang, O., Charlson, F., O’Neill, K., & Herrman, H. (2023). Transforming mental health systems globally: Principles and policy recommendations. The Lancet, 402(10402), 656–666. 10.1016/S0140-6736(23)00918-2. 37597892

[r64] Rabbitts, J. A., Palermo, T. M., Zhou, C., Meyyappan, A., & Chen, L. (2020). Psychosocial predictors of acute and chronic pain in adolescents undergoing major musculoskeletal surgery. The Journal of Pain, 21(11), 1236–1246. 10.1016/j.jpain.2020.02.004. 32553622 PMC7721978

[r65] Raja, S. N., Carr, D. B., Cohen, M., Finnerup, N. B., Flor, H., Gibson, S., Keefe, F. J., Mogil, J. S., Ringkamp, M., Sluka, K. A., Song, X.-J., Stevens, B., Sullivan, M. D., Tutelman, P. R., Ushida, T., & Vader, K. (2020). The revised International Association for the Study of Pain definition of pain: Concepts, challenges, and compromises. Pain, 161(9), 1976–1982. 10.1097/j.pain.0000000000001939. 32694387 PMC7680716

[r66] Reynolds, W. M. (1987). Suicidal ideation questionnaire. Odessa, FL: Psychological Assessment Resources. https://scholar.google.com/scholar_lookup?&title=Suicidal%20ideation%20questionnaire%20%28SIQ%29&publication_year=1987&author=Reynolds%2CWM

[r67] Richardson, J. R., Peacock, S. J., Hawthorne, G., Iezzi, A., Elsworth, G., & Day, N. A. (2012). Construction of the descriptive system for the assessment of quality of life AQoL-6D utility instrument. Health and Quality of Life Outcomes, 10(38), 1–9. 10.1186/1477-7525-10-38. 22507254 PMC3349491

[r68] Rickwood, D., McEachran, J., Saw, A., Telford, N., Trethowan, J., & McGorry, P. (2023). Sixteen years of innovation in youth mental healthcare: Outcomes for young people attending Australia’s headspace Centre services. PLoS One, 18(6), e0282040. 10.1371/journal.pone.0282040. 37390108 PMC10313045

[r69] Rickwood, D., Paraskakis, M., Quin, D., Hobbs, N., Ryall, V., Trethowan, J., & McGorry, P. (2018). Australia’s innovation in youth mental health care: The headspace Centre model. Early Intervention in Psychiatry, 13(1), 159–166. 10.1111/eip.12740. 30311423 PMC6585724

[r70] Roth-Isigkeit, A., Thyen, U., Stöven, H., Schwarzenberger, J., & Schmucker, P. (2005). Pain among children and adolescents: Restrictions in daily living and triggering factors. Pediatrics, 115(2), e152–e162. 10.1542/peds.2004-0682. 15687423

[r71] Serbic, D., Friedrich, C., & Murray, R. (2023). Psychological, social and academic functioning in university students with chronic pain: A systematic review. Journal of American College Health, 71(9), 2894–2908. 10.1080/07448481.2021.2006199. 34871522

[r72] Slater, H., Waller, R., Briggs, A. M., Lord, S. M., & Smith, A. J. (2025). Characterizing phenotypes and clinical and health utilization associations of young people with chronic pain: Latent class analysis using the electronic persistent pain outcomes collaboration database. Pain, 166(1), 67–86. 10.1097/j.pain.0000000000003326. 39688968 PMC11647817

[r73] Solmi, M., Radua, J., Olivola, M., Croce, E., Soardo, L., de Pablo, G., Il Shin, J., Kirkbride, J. B., Jones, P., Kim, J. H., Kim, J. Y., Carvalho, A. F., Seeman, M. V., Correll, C. U., & Fusar-Poli, P. (2022). Age at onset of mental disorders worldwide: Large-scale meta-analysis of 192 epidemiological studies. Molecular Psychiatry, 27(1), 281–295. 10.1038/s41380-021-01161-7. 34079068 PMC8960395

[r74] Soltani, S., Kopala-Sibley, D. C., & Noel, M. (2019). The co-occurrence of Pediatric chronic pain and depression. The Clinical Journal of Pain, 35(7), 633–643. 10.1097/AJP.0000000000000723. 31094934

[r75] Spitzer, R. L., Kroenke, K., Williams, J. B. W., & Löwe, B. (2006). A brief measure for assessing generalized anxiety disorder: The GAD-7. Archives of Internal Medicine, 166(10), 1092–1097. 10.1001/archinte.166.10.1092. 16717171

[r76] Tang, N. K. Y., & Crane, C. (2006). Suicidality in chronic pain: A review of the prevalence, risk factors and psychological links. Psychological Medicine, 36(05), 575. 10.1017/S0033291705006859. 16420727

[r77] *The Comprehensive R Archive Network* . (2024, August 11). https://cran.r-project.org/

[r78] Thielke, S. M., Fan, M.-Y., Sullivan, M., & Unützer, J. (2007). Pain limits the effectiveness of collaborative Care for Depression. The American Journal of Geriatric Psychiatry, 15(8), 699–707. 10.1097/JGP.0b013e3180325a2d. 17670998

[r79] Thompson, E. L., Broadbent, J., Fuller-Tyszkiewicz, M., Bertino, M. D., & Staiger, P. K. (2019). A network analysis of the links between chronic pain symptoms and affective disorder symptoms. International Journal of Behavioral Medicine, 26(1), 59–68. 10.1007/s12529-018-9754-8. 30377989

[r80] Treede, R.-D., Rief, W., Barke, A., Aziz, Q., Bennett, M. I., Benoliel, R., Cohen, M., Evers, S., Finnerup, N. B., First, M. B., Giamberardino, M. A., Kaasa, S., Korwisi, B., Kosek, E., Lavand’homme, P., Nicholas, M., Perrot, S., Scholz, J., Schug, S., … Wang, S.-J. (2019). Chronic pain as a symptom or a disease: The IASP classification of chronic pain for the International Classification of Diseases (ICD-11). Pain, 160(1), 19. 10.1097/j.pain.0000000000001384. 30586067

[r81] van Alboom, M., Elmer, T., Boersma, K., Forgeron, P., Baert, F., Bracke, P., & Goubert, L. (2022). Social integration of adolescents with chronic pain: A social network analysis. Pain, 163(11), 2232–2244. 10.1097/j.pain.0000000000002623. 35439797

[r82] Victor, T. W., Hu, X., Campbell, J., White, R. E., Buse, D. C., & Lipton, R. B. (2010). Association between migraine, anxiety and depression. Cephalalgia: An International Journal of Headache, 30(5), 567–575. 10.1111/j.1468-2982.2009.01944.x. 19614684

[r83] Vlaeyen, J. W. S., & Linton, S. J. (2000). Fear-avoidance and its consequences in chronic musculoskeletal pain: A state of the art. Pain, 85(3), 317–332. 10.1016/S0304-3959(99)00242-0. 10781906

[r84] von Elm, E., Altman, D. G., Egger, M., Pocock, S. J., Gøtzsche, P. C., Vandenbroucke, J. P., & Initiative, S. T. R. O. B. E. (2008). The strengthening the reporting of observational studies in epidemiology (STROBE) statement: Guidelines for reporting observational studies. Journal of Clinical Epidemiology, 61(4), 344–349. 10.1016/j.jclinepi.2007.11.008. 18313558

[r85] Walker, A. K., Kavelaars, A., Heijnen, C. J., & Dantzer, R. (2014). Neuroinflammation and comorbidity of pain and depression. Pharmacological Reviews, 66(1), 80–101. 10.1124/pr.113.008144. 24335193 PMC3880465

[r86] Wildeboer, E. M., Chambers, C. T., Soltani, S., & Noel, M. (2023). The relationship between chronic pain, depression, psychosocial factors, and Suicidality in adolescents. The Clinical Journal of Pain, 39(5), 226–235. 10.1097/AJP.0000000000001108. 36917771

[r87] Wise, T. N., Meyers, A. L., Dessaiah, D., Mallinckrodt, C. H., Robinson, M. J., & Kajdasz, D. K. (2008). The significance of treating somatic symptoms on functional outcome improvement in patients with major depressive disorder: A post hoc analysis of 2 trials. The Primary Care Companion to the Journal of Clinical Psychiatry, 10(04), 270–275. 10.4088/PCC.v10n0401. PMC2528237 18787676

[r88] World Health Organization. (2022). Mental disorders. https://www.who.int/news-room/fact-sheets/detail/mental-disorders

[r89] Zvolensky, M. J., Kauffman, B. Y., Shepherd, J. M., Viana, A. G., Bogiaizian, D., Rogers, A. H., Bakhshaie, J., & Peraza, N. (2020). Pain-related anxiety among latinx college students: Relations to body vigilance, worry, anxious arousal, and general depression. Journal of Racial and Ethnic Health Disparities, 7(3), 498–507. 10.1007/s40615-019-00678-6. 31845285 PMC7231647

