# Supplementary material for: Estimating the longitudinal association between pain characteristics and clinical outcomes in young people with mental ill-health
Source: Psychol Med. 2025 Jul 30;55:e207. doi: 10.1017/S0033291725101104 (PMC12341028; doi:10.1017/S0033291725101104)
Supplement: Oosterwijk et al. supplementary material 1 — Oosterwijk et al. supplementary material [file S0033291725101104sup001.docx]

**
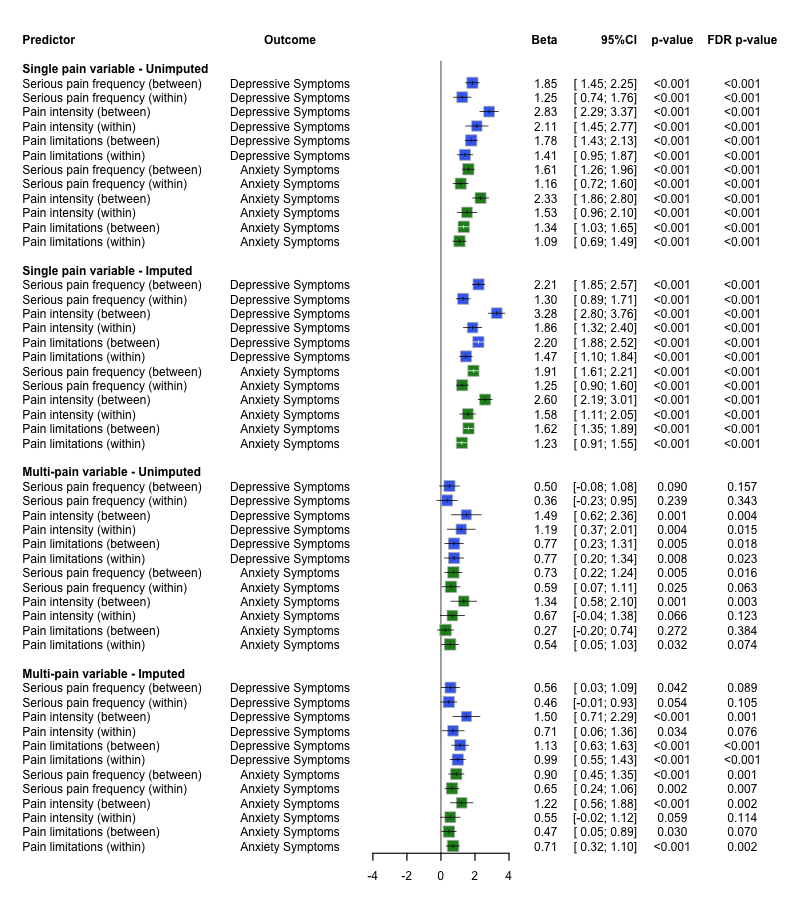
**

**Supplementary Figure 1.** Forest plot of beta coefficients and 95% confidence intervals of pain characteristics from confounder adjusted single and multi-pain variable linear mixed effects models with restricted maximum likelihood estimation for depressive and anxiety symptoms across data sets with and without multiple imputation. Between-participant estimates are the baseline score, indicating if baseline pain was associated with clinical outcomes across the three-month follow-up (level 2 exposure). Within‑participant estimates are baseline-centred indicating if a change from the baseline pain score was associated with a change in clinical outcome over time (level 1 exposure).

**
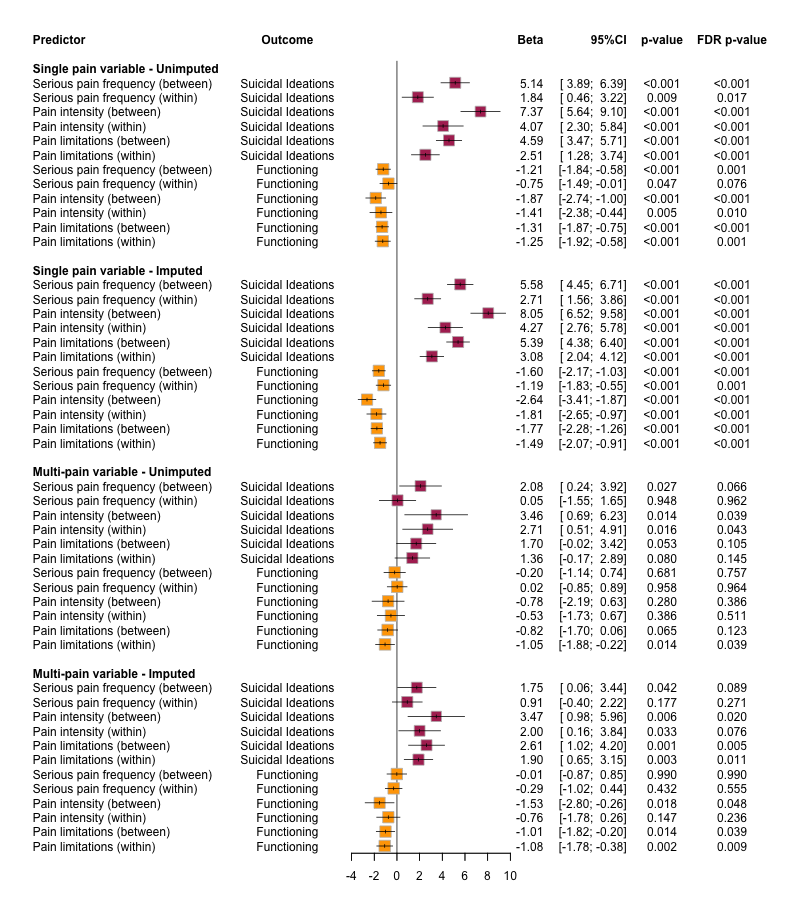
**

**Supplementary Figure 2.** Forest plot of beta coefficients and 95% confidence intervals of pain characteristics from confounder adjusted single and multi-pain variable linear mixed effects models with restricted maximum likelihood estimation for suicidal ideation and social and occupational functioning across data sets with and without multiple imputation. Between-participant estimates are the baseline score, indicating if baseline pain was associated with clinical outcomes across the three-month follow-up (level 2 exposure). Within‑participant estimates are baseline-centred indicating if a change from the baseline pain score was associated with a change in clinical outcome over time (level 1 exposure).


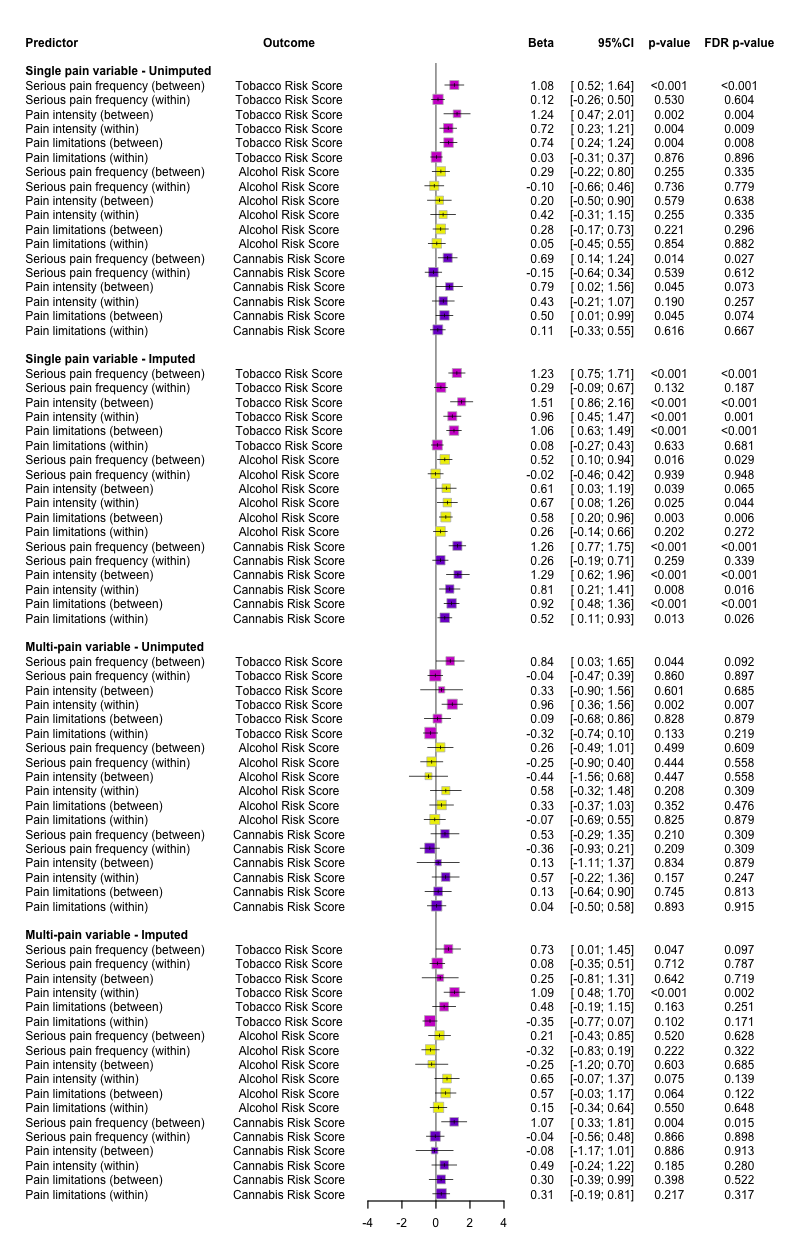


**Supplementary Figure 3.** Forest plot of beta coefficients and 95% confidence intervals of pain characteristics from confounder adjusted single and multi-pain variable linear mixed effects models with restricted maximum likelihood estimation for tobacco, alcohol, and cannabis risk scores across data sets with and without multiple imputation. Between-participant estimates are the baseline score, indicating if baseline pain was associated with clinical outcomes across the three-month follow-up (level 2 exposure). Within‑participant estimates are baseline-centred indicating if a change from the baseline pain score was associated with a change in clinical outcome over time (level 1 exposure).
