# Supplementary material for: Estimating the longitudinal association between pain characteristics and clinical outcomes in young people with mental ill-health
Source: Psychol Med. 2025 Jul 30;55:e207. doi: 10.1017/S0033291725101104 (PMC12341028; doi:10.1017/S0033291725101104)
Supplement: Oosterwijk et al. supplementary material 2 — Oosterwijk et al. supplementary material [file S0033291725101104sup002.docx]

**SUPPLEMENTARY TABLES**

[**Supplementary Table 1.** STROBE checklist. 4](#_Toc177374393)

[**Supplementary Table 2.** Baseline and follow-up data for predictors and outcomes. 6](#_Toc177374394)

[**Supplementary Table 3.** Number of participants with a non-zero risk score for substance use. 7](#_Toc177374395)

[**Supplementary Table 4.** Correlations between baseline centred pain predictors. 8](#_Toc177374396)

[**Supplementary Table 5.** Estimates across single pain variable models for both unadjusted and adjusted and unimputed and imputed data sets. 9](#_Toc177374397)

[**Supplementary Table 5.** Estimates across multi-pain variable models for both unadjusted and adjusted and unimputed and imputed data sets. 11](#_Toc177374398)

[**Supplementary Table 6.** Multi-level linear mixed effects regression of serious pain frequency on depressive symptoms. 13](#_Toc177374399)

[**Supplementary Table 7.** Multi-level linear mixed effects regression of pain intensity on depressive symptoms. 14](#_Toc177374400)

[**Supplementary Table 8.** Multi-level linear mixed effects regression of pain limitations on depressive symptoms. 15](#_Toc177374401)

[**Supplementary Table 9.** Multi-level linear mixed effects regression of multiple pain predictors on depressive symptoms. 16](#_Toc177374402)

[**Supplementary Table 10.** Multi-level linear mixed effects regression of serious pain frequency on anxiety symptoms. 17](#_Toc177374403)

[**Supplementary Table 11.** Multi-level linear mixed effects regression of pain intensity on anxiety symptoms. 18](#_Toc177374404)

[**Supplementary Table 12.** Multi-level linear mixed effects regression of pain limitations on anxiety symptoms. 19](#_Toc177374405)

[**Supplementary Table 13.** Multi-level linear mixed effects regression of multiple pain predictors on anxiety symptoms. 20](#_Toc177374406)

[**Supplementary Table 14.** Multi-level linear mixed effects regression of serious pain frequency on suicidal ideations. 21](#_Toc177374407)

[**Supplementary Table 15.** Multi-level linear mixed effects regression of pain intensity on suicidal ideations. 22](#_Toc177374408)

[**Supplementary Table 16.** Multi-level linear mixed effects regression of pain limitations on suicidal ideations. 23](#_Toc177374409)

[**Supplementary Table 17.** Multi-level linear mixed effects regression of multiple pain predictors on suicidal ideations. 24](#_Toc177374410)

[**Supplementary Table 18.** Multi-level linear mixed effects regression of serious pain frequency on social and occupational functioning. 25](#_Toc177374411)

[**Supplementary Table 19.** Multi-level linear mixed effects regression of pain intensity on social and occupational functioning. 26](#_Toc177374412)

[**Supplementary Table 20.** Multi-level linear mixed effects regression of pain limitations on social and occupational functioning. 27](#_Toc177374413)

[**Supplementary Table 21.** Multi-level linear mixed effects regression of multiple pain predictors on social and occupational functioning. 28](#_Toc177374414)

[**Supplementary Table 22.** Multi-level linear mixed effects regression of serious pain frequency on tobacco use risk scores. 29](#_Toc177374415)

[**Supplementary Table 23.** Multi-level linear mixed effects regression of pain intensity on tobacco use risk scores. 30](#_Toc177374416)

[**Supplementary Table 24.** Multi-level linear mixed effects regression of pain limitations on tobacco use risk scores. 31](#_Toc177374417)

[**Supplementary Table 25.** Multi-level linear mixed effects regression of multiple pain predictors on tobacco use risk scores. 32](#_Toc177374418)

[**Supplementary Table 26.** Multi-level linear mixed effects regression of serious pain frequency on alcohol use risk scores. 33](#_Toc177374419)

[**Supplementary Table 27.** Multi-level linear mixed effects regression of pain intensity on alcohol use risk scores. 34](#_Toc177374420)

[**Supplementary Table 28.** Multi-level linear mixed effects regression of pain limitations on alcohol use risk scores. 35](#_Toc177374421)

[**Supplementary Table 29.** Multi-level linear mixed effects regression of multiple pain predictors on alcohol use risk scores. 36](#_Toc177374422)

[**Supplementary Table 30.** Multi-level linear mixed effects regression of serious pain frequency on cannabis use risk scores. 37](#_Toc177374423)

[**Supplementary Table 31.** Multi-level linear mixed effects regression of pain intensity on cannabis use risk scores. 38](#_Toc177374424)

[**Supplementary Table 32.** Multi-level linear mixed effects regression of pain limitations on cannabis use risk scores. 39](#_Toc177374425)

[**Supplementary Table 33.** Multi-level linear mixed effects regression of multiple pain predictors on cannabis use risk scores. 40](#_Toc177374426)

| **Supplementary Table 1.** STROBE checklist. | | | | | |  |
| --- | --- | --- | --- | --- | --- | --- |
|  | | **Item No.** | **Recommendation** | **Relevant section in manuscript** | | |
| **Title and abstract** | | 1 | (*a*) Indicate the study’s design with a commonly used term in the title or the abstract | **Title page** | | |
|  |  |  | (*b*) Provide in the abstract an informative and balanced summary of what was done and what was found | **Abstract** | | |
|  | |  |  |  |  |  |
| Background/rationale | | 2 | Explain the scientific background and rationale for the investigation being reported | **Literature review** | | |
| Objectives | | 3 | State specific objectives, including any prespecified hypotheses | **Aims, hypotheses, and significance** | | |
| **Methods** | |  |  |  |  |  |
| Study design | | 4 | Present key elements of study design early in the paper | **Study design** | | |
| Setting | | 5 | Describe the setting, locations, and relevant dates, including periods of recruitment, exposure, follow-up, and data collection | **Setting** | | |
| Participants | | 6 | (*a*) *Cohort study*—Give the eligibility criteria, and the sources and methods of selection of participants. Describe methods of follow-up  *Case-control study*—Give the eligibility criteria, and the sources and methods of case ascertainment and control selection. Give the rationale for the choice of cases and controls  *Cross-sectional study*—Give the eligibility criteria, and the sources and methods of selection of participants | **Participants** | | |
|  |  |  | (*b*) *Cohort study*—For matched studies, give matching criteria and number of exposed and unexposed  *Case-control study*—For matched studies, give matching criteria and the number of controls per case | *NA* | | |
| Variables | | 7 | Clearly define all outcomes, exposures, predictors, potential confounders, and effect modifiers. Give diagnostic criteria, if applicable | **Variables** | | |
| Data sources/ measurement | | 8* | For each variable of interest, give sources of data and details of methods of assessment (measurement). Describe comparability of assessment methods if there is more than one group | **Data sources and measurement** | | |
| Bias | | 9 | Describe any efforts to address potential sources of bias | **Bias** | | |
| Study size | | 10 | Explain how the study size was arrived at | **Study size** | | |
| Quantitative variables | 11 | | Explain how quantitative variables were handled in the analyses. If applicable, describe which groupings were chosen and why | | **Quantitative variables** |  |
| Statistical methods | 12 | | (*a*) Describe all statistical methods, including those used to control for confounding | | **Statistical methods** |  |
|  |  |  | (*b*) Describe any methods used to examine subgroups and interactions | | **Statistical methods** |  |
|  |  |  | (*c*) Explain how missing data were addressed | | **Statistical methods** |  |
|  |  |  | (*d*) *Cohort study*—If applicable, explain how loss to follow-up was addressed  *Case-control study*—If applicable, explain how matching of cases and controls was addressed  *Cross-sectional study*—If applicable, describe analytical methods taking account of sampling strategy | | **Statistical methods** |  |
|  |  |  | (*e*) Describe any sensitivity analyses | | *NA* |  |
| **Results** | | | | | |  |
| Participants | 13* | | (a) Report numbers of individuals at each stage of study—eg numbers potentially eligible, examined for eligibility, confirmed eligible, included in the study, completing follow-up, and analysed | | **Participants** |  |
|  |  |  | (b) Give reasons for non-participation at each stage | | **Participants** |  |
|  |  |  | (c) Consider use of a flow diagram | | **Participants** |  |
| Descriptive data | 14* | | (a) Give characteristics of study participants (eg demographic, clinical, social) and information on exposures and potential confounders | | **Descriptive data** |  |
|  |  |  | (b) Indicate number of participants with missing data for each variable of interest | | **Descriptive data** |  |
|  |  |  | (c) *Cohort study*—Summarise follow-up time (eg, average and total amount) | | **Descriptive data** |  |
| Outcome data | 15* | | *Cohort study*—Report numbers of outcome events or summary measures over time | | **Outcome data** |  |
|  |  |  | *Case-control study—*Report numbers in each exposure category, or summary measures of exposure | | *NA* |  |
|  |  |  | *Cross-sectional study—*Report numbers of outcome events or summary measures | | *NA* |  |
| Main results | 16 | | (*a*) Give unadjusted estimates and, if applicable, confounder-adjusted estimates and their precision (eg, 95% confidence interval). Make clear which confounders were adjusted for and why they were included | | **Main results, Supplementary Tables** |  |
|  |  |  | (*b*) Report category boundaries when continuous variables were categorised | | *NA* |  |
|  |  |  | (*c*) If relevant, consider translating estimates of relative risk into absolute risk for a meaningful time period | | *NA* |  |
| Other analyses | 17 | | Report other analyses done—eg analyses of subgroups and interactions, and sensitivity analyses | | *NA* |  |
| **Discussion** | | | | | |  |
| Key results | 18 | | Summarise key results with reference to study objectives | | **Overview** |  |
| Limitations | 19 | | Discuss limitations of the study, taking into account sources of potential bias or imprecision. Discuss both direction and magnitude of any potential bias | | **Strengths and limitations** |  |
| Interpretation | 20 | | Give a cautious overall interpretation of results considering objectives, limitations, multiplicity of analyses, results from similar studies, and other relevant evidence | | **Discussion** |  |
| Generalisability | 21 | | Discuss the generalisability (external validity) of the study results | | **Discussion** |  |
| **Other information** | |  | | | |  |
| Funding | 22 | | Give the source of funding and the role of the funders for the present study and, if applicable, for the original study on which the present article is based | | **Funding** |  |

*Give information separately for cases and controls in case-control studies and, if applicable, for exposed and unexposed groups in cohort and cross-sectional studies.

**Note:** An Explanation and Elaboration article discusses each checklist item and gives methodological background and published examples of transparent reporting. The STROBE checklist is best used in conjunction with this article (freely available on the Web sites of PLoS Medicine at http://www.plosmedicine.org/, Annals of Internal Medicine at http://www.annals.org/, and Epidemiology at http://www.epidem.com/). Information on the STROBE Initiative is available at www.strobe-statement.org.

| **Supplementary Table 2.** Baseline and follow-up data for predictors and outcomes. | | |
| --- | --- | --- |
| **Characteristic** | **Baseline** N = 1,107^1^ | **Follow-up** N = 665^1^ |
| **Predictors** | | |
| **Serious pain frequency** |  |  |
| Very rarely | 605 (56%) | 402 (63%) |
| Less than once a week | 289 (27%) | 156 (24%) |
| Three to four times a week | 121 (11%) | 60 (9.3%) |
| Most of the time | 56 (5.2%) | 25 (3.9%) |
| Unknown | 36 | 22 |
| **Pain intensity** |  |  |
| None at all | 523 (49%) | 343 (53%) |
| I have moderate pain | 475 (44%) | 262 (41%) |
| I suffer from severe pain | 64 (6.0%) | 34 (5.3%) |
| I suffer unbearable pain | 9 (0.8%) | 4 (0.6%) |
| Unknown | 36 | 22 |
| **Pain limitations** |  |  |
| Never | 482 (45%) | 329 (51%) |
| Rarely | 332 (31%) | 185 (29%) |
| Sometimes | 177 (17%) | 99 (15%) |
| Often | 64 (6.0%) | 27 (4.2%) |
| Always | 16 (1.5%) | 3 (0.5%) |
| Unknown | 36 | 22 |
| **Outcomes** | | |
| **PHQ-9 Total score** | 12.8 (6.6) | 9.8 (6.5) |
| Unknown | 39 | 25 |
| **GAD-7 total score** | 10.4 (5.7) | 7.9 (5.5) |
| Unknown | 40 | 22 |
| **SIQ-JR total score** | 19.8 (20.3) | 15.5 (18.0) |
| Unknown | 40 | 25 |
| **SOFAS** | 65.2 (9.5) | 69.8 (10.2) |
| Unknown | 35 | 12 |
| **ASSIST total score for tobacco** | 4.7 (8.3) | 4.2 (8.0) |
| Unknown | 65 | 40 |
| **ASSIST total score for alcohol** | 6.0 (8.0) | 5.2 (7.2) |
| Unknown | 77 | 48 |
| **ASSIST total score for cannabis** | 3.6 (8.3) | 2.9 (7.6) |
| Unknown | 67 | 42 |
| PHQ-9 = Nine item Patient Health Questionnaire, GAD-7 = Seven item Generalised Anxiety Disorder Scale, SIQ-JR = Suicidal Ideation Questionnaire-Junior, SOFAS = Social and Occupational Functioning Assessment Scale, ASSIST = World Health Organisation Alcohol, Smoking, and Substance Involvement Screening Test.  ^1^n (%); Mean (SD) | | |

| Supplementary Table 3. Number of participants with a non-zero risk score for substance use. | |
| --- | --- |
| Substance | **Participants with a risk score >0** |
| Tobacco | 327 (32.9%) |
| Alcohol | 617 (62.8%) |
| Cannabis | 256 (25.8%) |
| Cocaine | 28 (2.8%) |
| Amphetamine | 80 (8.0%) |
| Inhalants | 11 (1.1%) |
| Sedatives | 102 (10.3%) |
| Hallucinogens | 52 (5.2%) |
| Opioids | 35 (3.5%) |

| **Supplementary Table 4.** Correlations between baseline centred pain predictors. | | |
| --- | --- | --- |
| **Variable 1** | **Variable 2** | ***r*** |
| Serious pain frequency (between) | Serious pain frequency (between) | 1.00 |
| Serious pain frequency (within) | Serious pain frequency (between) | -0.31 |
| Pain intensity (between) | Serious pain frequency (between) | 0.68 |
| Pain intensity (within) | Serious pain frequency (between) | -0.11 |
| Pain limitations (between) | Serious pain frequency (between) | 0.67 |
| Pain limitations (within) | Serious pain frequency (between) | -0.16 |
| Serious pain frequency (between) | Serious pain frequency (within) | -0.31 |
| Serious pain frequency (within) | Serious pain frequency (within) | 1.00 |
| Pain intensity (between) | Serious pain frequency (within) | -0.14 |
| Pain intensity (within) | Serious pain frequency (within) | 0.44 |
| Pain limitations (between) | Serious pain frequency (within) | -0.15 |
| Pain limitations (within) | Serious pain frequency (within) | 0.46 |
| Serious pain frequency (between) | Pain intensity (between) | 0.68 |
| Serious pain frequency (within) | Pain intensity (between) | -0.14 |
| Pain intensity (between) | Pain intensity (between) | 1.00 |
| Pain intensity (within) | Pain intensity (between) | -0.31 |
| Pain limitations (between) | Pain intensity (between) | 0.73 |
| Pain limitations (within) | Pain intensity (between) | -0.22 |
| Serious pain frequency (between) | Pain intensity (within) | -0.11 |
| Serious pain frequency (within) | Pain intensity (within) | 0.44 |
| Pain intensity (between) | Pain intensity (within) | -0.31 |
| Pain intensity (within) | Pain intensity (within) | 1.00 |
| Pain limitations (between) | Pain intensity (within) | -0.17 |
| Pain limitations (within) | Pain intensity (within) | 0.55 |
| Serious pain frequency (between) | Pain limitations (between) | 0.67 |
| Serious pain frequency (within) | Pain limitations (between) | -0.15 |
| Pain intensity (between) | Pain limitations (between) | 0.73 |
| Pain intensity (within) | Pain limitations (between) | -0.17 |
| Pain limitations (between) | Pain limitations (between) | 1.00 |
| Pain limitations (within) | Pain limitations (between) | -0.34 |
| Serious pain frequency (between) | Pain limitations (within) | -0.16 |
| Serious pain frequency (within) | Pain limitations (within) | 0.46 |
| Pain intensity (between) | Pain limitations (within) | -0.22 |
| Pain intensity (within) | Pain limitations (within) | 0.55 |
| Pain limitations (between) | Pain limitations (within) | -0.34 |
| Pain limitations (within) | Pain limitations (within) | 1.00 |

#

| **Supplementary Table 5.** Estimates across single pain variable models for both unadjusted and adjusted and unimputed and imputed data sets. | | | | | | | | | | | | | |
| --- | --- | --- | --- | --- | --- | --- | --- | --- | --- | --- | --- | --- | --- |
|  |  | **Unadjusted: Unimputed** | | | **Unadjusted: Imputed** | | | **Adjusted: Unimputed** | | | **Adjusted: Imputed** | | |
| **Outcome** | **Variable** | **Beta (95%CI)** | **p-value** | **FDR p-value** | **Beta (95%CI)** | **p-value** | **FDR p-value** | **Beta (95%CI)** | **p-value** | **FDR p-value** | **Beta (95%CI)** | **p-value** | **FDR p-value** |
| Depressive Symptoms | Serious pain frequency (between) | **1.98 (1.57, 2.40)** | **<0.001** | **<0.001** | **2.40 (2.01, 2.78)** | **<0.001** | **<0.001** | **1.85 (1.45, 2.25)** | **<0.001** | **<0.001** | **2.21 (1.86, 2.57)** | **<0.001** | **<0.001** |
| Depressive Symptoms | Serious pain frequency (within) | **1.71 (1.16, 2.26)** | **<0.001** | **<0.001** | **1.83 (1.39, 2.27)** | **<0.001** | **<0.001** | **1.25 (0.74, 1.77)** | **<0.001** | **<0.001** | **1.30 (0.89, 1.70)** | **<0.001** | **<0.001** |
| Depressive Symptoms | Pain intensity (between) | **3.13 (2.57,3.68)** | **<0.001** | **<0.001** | **3.67 (3.16, 4.19)** | **<0.001** | **<0.001** | **2.83 (2.29, 3.37)** | **<0.001** | **<0.001** | **3.28 (2.79, 3.76)** | **<0.001** | **<0.001** |
| Depressive Symptoms | Pain intensity (within) | **2.61 (1.90, 3.32)** | **<0.001** | **<0.001** | **2.57 (1.99,3.15)** | **<0.001** | **<0.001** | **2.11 (1.45, 2.78)** | **<0.001** | **<0.001** | **1.86 (1.33, 2.40)** | **<0.001** | **<0.001** |
| Depressive Symptoms | Pain limitations (between) | **2.02 (1.66, 2.39)** | **<0.001** | **<0.001** | **2.47 (2.13, 2.81)** | **<0.001** | **<0.001** | **1.78 (1.43, 2.13)** | **<0.001** | **<0.001** | **2.20 (1.88, 2.52)** | **<0.001** | **<0.001** |
| Depressive Symptoms | Pain limitations (within) | **1.99 (1.51, 2.47)** | **<0.001** | **<0.001** | **1.99 (1.60, 2.38)** | **<0.001** | **<0.001** | **1.41 (0.95, 1.86)** | **<0.001** | **<0.001** | **1.47 (1.10, 1.83)** | **<0.001** | **<0.001** |
| Anxiety Symptoms | Serious pain frequency (between) | **1.74 (1.39, 2.10)** | **<0.001** | **<0.001** | **2.03 (1.71, 2.34)** | **<0.001** | **<0.001** | **1.61 (1.27, 1.96)** | **<0.001** | **<0.001** | **1.91 (1.61, 2.21)** | **<0.001** | **<0.001** |
| Anxiety Symptoms | Serious pain frequency (within) | **1.49 (1.02, 1.95)** | **<0.001** | **<0.001** | **1.65 (1.28, 2.03)** | **<0.001** | **<0.001** | **1.16 (0.72, 1.60)** | **<0.001** | **<0.001** | **1.25 (0.90, 1.61)** | **<0.001** | **<0.001** |
| Anxiety Symptoms | Pain intensity (between) | **2.54 (2.06, 3.01)** | **<0.001** | **<0.001** | **2.83 (2.41, 3.26)** | **<0.001** | **<0.001** | **2.33 (1.85, 2.80)** | **<0.001** | **<0.001** | **2.60 (2.19, 3.00)** | **<0.001** | **<0.001** |
| Anxiety Symptoms | Pain intensity (within) | **1.90 (1.30, 2.51)** | **<0.001** | **<0.001** | **2.07 (1.58, 2.57)** | **<0.001** | **<0.001** | **1.53 (0.96, 2.11)** | **<0.001** | **<0.001** | **1.58 (1.12, 2.05)** | **<0.001** | **<0.001** |
| Anxiety Symptoms | Pain limitations (between) | **1.53 (1.22, 1.85)** | **<0.001** | **<0.001** | **1.80 (1.53, 2.08)** | **<0.001** | **<0.001** | **1.34 (1.03, 1.65)** | **<0.001** | **<0.001** | **1.62 (1.35, 1.89)** | **<0.001** | **<0.001** |
| Anxiety Symptoms | Pain limitations (within) | **1.51 (1.10, 1.92)** | **<0.001** | **<0.001** | **1.61 (1.27, 1.95)** | **<0.001** | **<0.001** | **1.09 (0.69, 1.48)** | **<0.001** | **<0.001** | **1.23 (0.91, 1.55)** | **<0.001** | **<0.001** |
| Suicidal Ideations | Serious pain frequency (between) | **5.15 (3.88, 6.41)** | **<0.001** | **<0.001** | **5.90 (4.74, 7.05)** | **<0.001** | **<0.001** | **5.14 (3.88, 6.39)** | **<0.001** | **<0.001** | **5.58 (4.45, 6.71)** | **<0.001** | **<0.001** |
| Suicidal Ideations | Serious pain frequency (within) | **2.42 (1.05, 3.79)** | **0.001** | **0.001** | **3.28 (2.13, 4.44)** | **<0.001** | **<0.001** | **1.84 (0.46, 3.22)** | **0.009** | **0.017** | **2.71 (1.56, 3.85)** | **<0.001** | **<0.001** |
| Suicidal Ideations | Pain intensity (between) | **7.59 (5.87, 9.31)** | **<0.001** | **<0.001** | **8.65 (7.08, 10.21)** | **<0.001** | **<0.001** | **7.37 (5.64, 9.10)** | **<0.001** | **<0.001** | **8.05 (6.52, 9.58)** | **<0.001** | **<0.001** |
| Suicidal Ideations | Pain intensity (within) | **4.61 (2.84, 6.37)** | **<0.001** | **<0.001** | **5.17 (3.65, 6.70)** | **<0.001** | **<0.001** | **4.07 (2.30, 5.85)** | **<0.001** | **<0.001** | **4.27 (2.76, 5.78)** | **<0.001** | **<0.001** |
| Suicidal Ideations | Pain limitations (between) | **4.72 (3.60, 5.84)** | **<0.001** | **<0.001** | **5.78 (4.75, 6.81)** | **<0.001** | **<0.001** | **4.59 (3.48, 5.71)** | **<0.001** | **<0.001** | **5.39 (4.38, 6.39)** | **<0.001** | **<0.001** |
| Suicidal Ideations | Pain limitations (within) | **3.26 (2.04, 4.47)** | **<0.001** | **<0.001** | **3.77 (2.73, 4.81)** | **<0.001** | **<0.001** | **2.51 (1.28, 3.74)** | **<0.001** | **<0.001** | **3.08 (2.04, 4.12)** | **<0.001** | **<0.001** |
| Functioning | Serious pain frequency (between) | **-1.46 (-2.10, -0.81)** | **<0.001** | **<0.001** | **-1.83 (-2.42, -1.25)** | **<0.001** | **<0.001** | **-1.21 (-1.85, -0.58)** | **<0.001** | **0.001** | **-1.60 (-2.17, -1.02)** | **<0.001** | **<0.001** |
| Functioning | Serious pain frequency (within) | **-1.47 (-2.28, -0.66)** | **<0.001** | **0.001** | **-1.91 (-2.59, -1.23)** | **<0.001** | **<0.001** | **-0.75 (-1.50, -0.01)** | **0.047** | **0.076** | **-1.19 (-1.82, -0.55)** | **<0.001** | **0.001** |
| Functioning | Pain intensity (between) | **-2.34 (-3.21, -1.46)** | **<0.001** | **<0.001** | **-3.03 (-3.82, -2.24)** | **<0.001** | **<0.001** | **-1.87 (-2.75, -1.00)** | **<0.001** | **<0.001** | **-2.64 (-3.42, -1.87)** | **<0.001** | **<0.001** |
| Functioning | Pain intensity (within) | **-2.13 (-3.17, -1.08)** | **<0.001** | **<0.001** | **-2.64 (-3.54, -1.74)** | **<0.001** | **<0.001** | **-1.41 (-2.38, -0.44)** | **0.005** | **0.010** | **-1.81 (-2.65, -0.97)** | **<0.001** | **<0.001** |
| Functioning | Pain limitations (between) | **-1.53 (-2.10, -0.96)** | **<0.001** | **<0.001** | **-1.98 (-2.50, -1.46)** | **<0.001** | **<0.001** | **-1.31 (-1.88, -0.75)** | **<0.001** | **<0.001** | **-1.77 (-2.27, -1.26)** | **<0.001** | **<0.001** |
| Functioning | Pain limitations (within) | **-2.06 (-2.77, -1.35)** | **<0.001** | **<0.001** | **-2.15 (-2.76, -1.53)** | **<0.001** | **<0.001** | **-1.25 (-1.92, -0.59)** | **<0.001** | **0.001** | **-1.49 (-2.06, -0.91)** | **<0.001** | **<0.001** |
| Tobacco Risk Score | Serious pain frequency (between) | **1.03 (0.47, 1.59)** | **<0.001** | **<0.001** | **1.23 (0.73,1.72)** | **<0.001** | **<0.001** | **1.08 (0.52, 1.64)** | **<0.001** | **<0.001** | **1.23 (0.75, 1.71)** | **<0.001** | **<0.001** |
| Tobacco Risk Score | Serious pain frequency (within) | 0.13 (-0.23, 0.50) | 0.482 | 0.519 | 0.30 (-0.08, 0.68) | 0.117 | 0.139 | 0.12 (-0.26, 0.50) | 0.530 | 0.604 | 0.29 (-0.09, 0.67) | 0.132 | 0.187 |
| Tobacco Risk Score | Pain intensity (between) | **1.40 (0.63, 2.17)** | **<0.001** | **0.001** | **1.61 (0.94, 2.28)** | **<0.001** | **<0.001** | **1.24 (0.47, 2.01)** | **0.002** | **0.004** | **1.51 (0.85, 2.16)** | **<0.001** | **<0.001** |
| Tobacco Risk Score | Pain intensity (within) | **0.71 (0.23, 1.19)** | **0.004** | **0.005** | **0.99 (0.49, 1.49)** | **<0.001** | **<0.001** | **0.72 (0.23,1.21)** | **0.004** | **0.009** | **0.96 (0.45, 1.46)** | **<0.001** | **0.001** |
| Tobacco Risk Score | Pain limitations (between) | **0.83 (0.33, 1.33)** | **0.001** | **0.002** | **1.08 (0.64, 1.52)** | **<0.001** | **<0.001** | **0.74 (0.24, 1.24)** | **0.004** | **0.008** | **1.06 (0.63, 1.48)** | **<0.001** | **<0.001** |
| Tobacco Risk Score | Pain limitations (within) | 0.06 (-0.27, 0.39) | 0.713 | 0.726 | 0.12 (-0.23, 0.46) | 0.507 | 0.536 | 0.03 (-0.31, 0.37) | 0.876 | 0.896 | 0.08 (-0.26, 0.43) | 0.633 | 0.681 |
| Alcohol Risk Score | Serious pain frequency (between) | 0.21 (-0.31, 0.73) | 0.433 | 0.470 | **0.51 (0.06, 0.96)** | **0.025** | **0.032** | 0.29 (-0.21, 0.80) | 0.255 | 0.335 | **0.52 (0.10, 0.95)** | **0.016** | **0.029** |
| Alcohol Risk Score | Serious pain frequency (within) | -0.06 (-0.62, 0.49) | 0.822 | 0.829 | 0.04 (-0.40, 0.49) | 0.852 | 0.852 | -0.10 (-0.65, 0.46) | 0.736 | 0.779 | -0.02 (-0.46, 0.43) | 0.939 | 0.948 |
| Alcohol Risk Score | Pain intensity (between) | 0.48 (-0.24, 1.19) | 0.191 | 0.214 | **0.80 (0.19, 1.40)** | **0.010** | **0.014** | 0.20 (-0.50, 0.90) | 0.579 | 0.638 | 0.61 (0.03, 1.19) | 0.039 | 0.065 |
| Alcohol Risk Score | Pain intensity (within) | 0.42 (-0.30, 1.13) | 0.256 | 0.284 | **0.78 (0.19, 1.37)** | **0.009** | **0.013** | 0.42 (-0.31, 1.15) | 0.255 | 0.335 | **0.67 (0.09, 1.26)** | **0.025** | **0.044** |
| Alcohol Risk Score | Pain limitations (between) | 0.40 (-0.06, 0.87) | 0.085 | 0.104 | **0.65 (0.25, 1.05)** | **0.001** | **0.002** | 0.28 (-0.17, 0.73) | 0.221 | 0.296 | **0.58 (0.21, 0.96)** | **0.003** | **0.006** |
| Alcohol Risk Score | Pain limitations (within) | 0.17 (-0.32, 0.66) | 0.490 | 0.522 | 0.33 (-0.07, 0.74) | 0.106 | 0.128 | 0.05 (-0.45, 0.55) | 0.854 | 0.882 | 0.26 (-0.14, 0.67) | 0.202 | 0.272 |
| Cannabis Risk Score | Serious pain frequency (between) | **0.62 (0.08, 1.17)** | **0.025** | **0.032** | **1.29 (0.79,1.78)** | **<0.001** | **<0.001** | **0.69 (0.14, 1.25)** | **0.014** | **0.027** | **1.26 (0.77, 1.75)** | **<0.001** | **<0.001** |
| Cannabis Risk Score | Serious pain frequency (within) | -0.10 (-0.57, 0.37) | 0.675 | 0.693 | 0.31 (-0.14, 0.76) | 0.177 | 0.203 | -0.15 (-0.64, 0.33) | 0.539 | 0.612 | 0.26 (-0.19, 0.71) | 0.259 | 0.339 |
| Cannabis Risk Score | Pain intensity (between) | **0.80 (0.04, 1.55)** | **0.038** | **0.048** | **1.40 (0.73, 2.08)** | **<0.001** | **<0.001** | 0.79 (0.02, 1.55) | 0.045 | 0.073 | **1.29 (0.62, 1.96)** | **<0.001** | **<0.001** |
| Cannabis Risk Score | Pain intensity (within) | 0.42 (-0.20, 1.05) | 0.186 | 0.210 | **0.89 (0.29, 1.48)** | **0.003** | **0.005** | 0.43 (-0.21, 1.07) | 0.190 | 0.257 | **0.81 (0.21, 1.41)** | **0.008** | **0.016** |
| Cannabis Risk Score | Pain limitations (between) | 0.50 (0.02, 0.99) | 0.042 | 0.051 | **0.96 (0.52, 1.40)** | **<0.001** | **<0.001** | 0.50 (0.01, 1.00) | 0.045 | 0.074 | **0.92 (0.48, 1.36)** | **<0.001** | **<0.001** |
| Cannabis Risk Score | Pain limitations (within) | 0.17 (-0.25, 0.60) | 0.422 | 0.463 | **0.57 (0.17, 0.98)** | **0.006** | **0.008** | 0.11 (-0.33, 0.55) | 0.616 | 0.667 | **0.52 (0.11, 0.93)** | **0.013** | **0.026** |
| FDR = False Discovery Rate. Bold indicates significance after FDR p-value adjustment.  *Between-subject estimates are the baseline score, indicating if baseline pain was associated with clinical outcomes across the three-month follow-up (level 2 predictor). Within-subject estimates are baseline-centred indicating if a fluctuation from the baseline pain score was associated with the clinical outcome (level 1 predictor). | | | | | | | | | | | | | |

| **Supplementary Table 6.** Estimates across multi-pain variable models for both unadjusted and adjusted and unimputed and imputed data sets. | | | | | | | | | | | | | |
| --- | --- | --- | --- | --- | --- | --- | --- | --- | --- | --- | --- | --- | --- |
|  |  | **Unadjusted: Unimputed** | | | **Unadjusted: Imputed** | | | **Adjusted: Unimputed** | | | **Adjusted: Imputed** | | |
| **Outcome** | **Variable*** | **β (95%CI)** | **p-value** | **FDR p-value** | **β (95%CI)** | **p-value** | **FDR p-value** | **β (95%CI)** | **p-value** | **FDR p-value** | **β (95%CI)** | **p-value** | **FDR p-value** |
| Depressive Symptoms | Serious pain frequency (between) | 0.34 (-0.26, 0.94) | 0.264 | 0.376 | 0.39 (-0.18,0.96) | 0.184 | 0.298 | 0.50 (-0.08, 1.09) | 0.090 | 0.157 | 0.56 (0.02, 1.09) | 0.042 | 0.089 |
| Depressive Symptoms | Serious pain frequency (within) | 0.51 (-0.12, 1.15) | 0.114 | 0.205 | 0.68 (0.17, 1.19) | 0.009 | 0.027 | 0.36 (-0.24, 0.95) | 0.239 | 0.343 | 0.46 (-0.01, 0.93) | 0.054 | 0.105 |
| Depressive Symptoms | Pain intensity (between) | **1.73 (0.84, 2.62)** | **<0.001** | **0.002** | **1.82 (0.98, 2.66)** | **<0.001** | **<0.001** | **1.49 (0.62, 2.37)** | **0.001** | **0.004** | **1.50 (0.71, 2.28)** | **<0.001** | **0.001** |
| Depressive Symptoms | Pain intensity (within) | **1.16 (0.28, 2.04)** | **0.010** | **0.029** | **1.02 (0.31, 1.73)** | **0.005** | **0.019** | **1.19 (0.37, 2.01)** | **0.004** | **0.015** | 0.71 (0.05, 1.36) | 0.034 | 0.076 |
| Depressive Symptoms | Pain limitations (between) | **0.99 (0.43, 1.55)** | **0.001** | **0.003** | **1.33 (0.80, 1.87)** | **<0.001** | **<0.001** | **0.77 (0.23, 1.31)** | **0.005** | **0.018** | **1.13 (0.63, 1.63)** | **<0.001** | **<0.001** |
| Depressive Symptoms | Pain limitations (within) | **1.30 (0.69, 1.90)** | **<0.001** | **<0.001** | **1.32 (0.84, 1.80)** | **<0.001** | **<0.001** | **0.77 (0.20, 1.34)** | **0.008** | **0.023** | **0.99 (0.54, 1.43)** | **<0.001** | **<0.001** |
| Anxiety Symptoms | Serious pain frequency (between) | **0.69 (0.17, 1.21)** | **0.009** | **0.027** | **0.80 (0.32,1.27)** | **0.001** | **0.005** | **0.73 (0.22, 1.24)** | **0.005** | **0.016** | **0.90 (0.45, 1.35)** | **<0.001** | **0.001** |
| Anxiety Symptoms | Serious pain frequency (within) | **0.71 (0.16, 1.25)** | **0.011** | **0.031** | **0.84 (0.40, 1.27)** | **<0.001** | **0.002** | 0.59 (0.08, 1.11) | 0.025 | 0.063 | **0.65 (0.24, 1.06)** | **0.002** | **0.007** |
| Anxiety Symptoms | Pain intensity (between) | **1.38 (0.61, 2.15)** | **<0.001** | **0.003** | **1.38 (0.68, 2.07)** | **<0.001** | **0.001** | **1.34 (0.58, 2.10)** | **0.001** | **0.003** | **1.22 (0.56, 1.89)** | **<0.001** | **0.002** |
| Anxiety Symptoms | Pain intensity (within) | 0.64 (-0.12,1.39) | 0.097 | 0.185 | **0.74 (0.13, 1.34)** | **0.018** | **0.047** | 0.67 (-0.04, 1.38) | 0.066 | 0.123 | 0.55 (-0.02, 1.12) | 0.059 | 0.114 |
| Anxiety Symptoms | Pain limitations (between) | 0.46 (-0.02, 0.94) | 0.059 | 0.132 | **0.63 (0.19, 1.07)** | **0.005** | **0.019** | 0.27 (-0.21, 0.74) | 0.272 | 0.384 | **0.47 (0.05, 0.89)** | **0.030** | **0.070** |
| Anxiety Symptoms | Pain limitations (within) | **0.92 (0.40, 1.44)** | **0.001** | **0.003** | **0.95 (0.53, 1.36)** | **<0.001** | **<0.001** | 0.54 (0.05, 1.04) | 0.032 | 0.074 | **0.71 (0.32, 1.10)** | **<0.001** | **0.002** |
| Suicidal Ideations | Serious pain frequency (between) | 1.80 (-0.05, 3.65) | 0.056 | 0.130 | 1.61 (-0.12, 3.34) | 0.068 | 0.146 | 2.08 (0.24, 3.93) | 0.027 | 0.066 | 1.75 (0.07, 3.44) | 0.042 | 0.089 |
| Suicidal Ideations | Serious pain frequency (within) | 0.31 (-1.29, 1.90) | 0.707 | 0.781 | 1.07 (-0.26, 2.40) | 0.114 | 0.205 | 0.05 (-1.55, 1.66) | 0.948 | 0.962 | 0.91 (-0.41, 2.22) | 0.177 | 0.271 |
| Suicidal Ideations | Pain intensity (between) | **3.94 (1.18, 6.71)** | **0.005** | **0.019** | **3.92 (1.37, 6.47)** | **0.003** | **0.011** | **3.46 (0.69, 6.24)** | **0.014** | **0.039** | **3.47 (0.98, 5.96)** | **0.006** | **0.020** |
| Suicidal Ideations | Pain intensity (within) | 2.56 (0.36, 4.75) | 0.023 | 0.058 | **2.45 (0.59, 4.31)** | **0.010** | **0.029** | **2.71 (0.51, 4.92)** | **0.016** | **0.043** | 2.00 (0.16, 3.84) | 0.033 | 0.076 |
| Suicidal Ideations | Pain limitations (between) | 1.75 (0.03, 3.48) | 0.046 | 0.111 | **2.86 (1.23, 4.48)** | **0.001** | **0.003** | 1.70 (-0.02, 3.43) | 0.053 | 0.105 | **2.61 (1.02, 4.19)** | **0.001** | **0.005** |
| Suicidal Ideations | Pain limitations (within) | **2.06 (0.54, 3.58)** | **0.008** | **0.027** | **2.36 (1.09, 3.62)** | **<0.001** | **0.002** | 1.36 (-0.16, 2.89) | 0.080 | 0.145 | **1.90 (0.64, 3.15)** | **0.003** | **0.011** |
| Functioning | Serious pain frequency (between) | -0.19 (-1.15, 0.76) | 0.691 | 0.774 | -0.01 (-0.90, 0.88) | 0.986 | 0.986 | -0.20 (-1.14, 0.74) | 0.681 | 0.757 | -0.01 (-0.87, 0.86) | 0.990 | 0.990 |
| Functioning | Serious pain frequency (within) | -0.29 (-1.23, 0.66) | 0.549 | 0.678 | -0.68 (-1.47, 0.11) | 0.093 | 0.183 | 0.02 (-0.85, 0.89) | 0.958 | 0.964 | -0.29 (-1.03, 0.44) | 0.432 | 0.555 |
| Functioning | Pain intensity (between) | -1.32 (-2.74, 0.10) | 0.069 | 0.146 | **-1.88 (-3.18, -0.57)** | **0.005** | **0.019** | -0.78 (-2.19, 0.63) | 0.280 | 0.386 | **-1.53 (-2.80, -0.26)** | **0.018** | **0.048** |
| Functioning | Pain intensity (within) | -0.52 (-1.81, 0.78) | 0.436 | 0.555 | -1.06 (-2.16, 0.04) | 0.060 | 0.132 | -0.53 (-1.73, 0.67) | 0.386 | 0.511 | -0.76 (-1.78, 0.27) | 0.147 | 0.236 |
| Functioning | Pain limitations (between) | -0.78 (-1.67, 0.11) | 0.086 | 0.172 | **-1.05 (-1.88, -0.21)** | **0.014** | **0.037** | -0.82 (-1.70, 0.05) | 0.065 | 0.123 | **-1.01 (-1.82, -0.20)** | **0.014** | **0.039** |
| Functioning | Pain limitations (within) | **-1.72 (-2.61, -0.82)** | **<0.001** | **0.002** | **-1.47 (-2.22, -0.73)** | **<0.001** | **0.001** | **-1.05 (-1.88, -0.21)** | **0.014** | **0.039** | **-1.08 (-1.78, -0.38)** | **0.002** | **0.009** |
| Tobacco Risk Score | Serious pain frequency (between) | 0.55 (-0.27, 1.37) | 0.188 | 0.298 | 0.62 (-0.12,1.35) | 0.101 | 0.189 | 0.84 (0.02, 1.66) | 0.044 | 0.092 | 0.73 (0.01, 1.44) | 0.047 | 0.097 |
| Tobacco Risk Score | Serious pain frequency (within) | -0.04 (-0.46, 0.38) | 0.841 | 0.872 | 0.06 (-0.37,0.50) | 0.771 | 0.820 | -0.04 (-0.47, 0.40) | 0.860 | 0.897 | 0.08 (-0.35, 0.52) | 0.712 | 0.787 |
| Tobacco Risk Score | Pain intensity (between) | 0.63 (-0.60, 1.86) | 0.314 | 0.432 | 0.51 (-0.58,1.60) | 0.359 | 0.479 | 0.33 (-0.91, 1.56) | 0.601 | 0.685 | 0.25 (-0.81, 1.31) | 0.642 | 0.719 |
| Tobacco Risk Score | Pain intensity (within) | **0.93 (0.34, 1.52)** | **0.002** | **0.009** | **1.13 (0.52,1.74)** | **<0.001** | **0.002** | **0.96 (0.36, 1.56)** | **0.002** | **0.007** | **1.09 (0.48, 1.70)** | **<0.001** | **0.002** |
| Tobacco Risk Score | Pain limitations (between) | 0.20 (-0.57, 0.97) | 0.604 | 0.724 | 0.44 (-0.25,1.14) | 0.210 | 0.318 | 0.09 (-0.68, 0.86) | 0.828 | 0.879 | 0.48 (-0.19, 1.15) | 0.163 | 0.251 |
| Tobacco Risk Score | Pain limitations (within) | -0.27 (-0.68, 0.13) | 0.187 | 0.298 | -0.33 (-0.74,0.09) | 0.122 | 0.214 | -0.32 (-0.73, 0.10) | 0.133 | 0.219 | -0.35 (-0.76, 0.07) | 0.102 | 0.171 |
| Alcohol Risk Score | Serious pain frequency (between) | -0.23 (-0.99, 0.54) | 0.557 | 0.679 | -0.03 (-0.70, 0.65) | 0.942 | 0.953 | 0.26 (-0.49, 1.00) | 0.499 | 0.609 | 0.21 (-0.43, 0.85) | 0.520 | 0.628 |
| Alcohol Risk Score | Serious pain frequency (within) | -0.32 (-0.97, 0.33) | 0.335 | 0.453 | -0.34 (-0.85, 0.18) | 0.200 | 0.311 | -0.25 (-0.91, 0.40) | 0.444 | 0.558 | -0.32 (-0.83, 0.19) | 0.222 | 0.322 |
| Alcohol Risk Score | Pain intensity (between) | 0.19 (-0.96,1.33) | 0.750 | 0.818 | 0.15 (-0.85, 1.15) | 0.765 | 0.820 | -0.44 (-1.56, 0.69) | 0.447 | 0.558 | -0.25 (-1.20, 0.70) | 0.603 | 0.685 |
| Alcohol Risk Score | Pain intensity (within) | 0.50 (-0.40, 1.40) | 0.275 | 0.385 | 0.77 (0.05, 1.49) | 0.037 | 0.091 | 0.58 (-0.32, 1.49) | 0.208 | 0.309 | 0.65 (-0.07, 1.37) | 0.075 | 0.139 |
| Alcohol Risk Score | Pain limitations (between) | 0.45 (-0.26, 1.17) | 0.216 | 0.318 | 0.58 (-0.05, 1.22) | 0.073 | 0.149 | 0.33 (-0.37, 1.03) | 0.352 | 0.476 | 0.57 (-0.03, 1.17) | 0.064 | 0.122 |
| Alcohol Risk Score | Pain limitations (within) | 0.12 (-0.49, 0.74) | 0.691 | 0.774 | 0.19 (-0.30, 0.68) | 0.446 | 0.559 | -0.07 (-0.69, 0.55) | 0.825 | 0.879 | 0.15 (-0.34, 0.64) | 0.550 | 0.648 |
| Cannabis Risk Score | Serious pain frequency (between) | 0.37 (-0.44, 1.18) | 0.375 | 0.492 | **1.00 (0.25, 1.75)** | **0.009** | **0.027** | 0.53 (-0.30, 1.35) | 0.210 | 0.309 | **1.07 (0.33, 1.81)** | **0.004** | **0.015** |
| Cannabis Risk Score | Serious pain frequency (within) | -0.33 (-0.88, 0.22) | 0.240 | 0.348 | -0.04 (-0.56, 0.47) | 0.869 | 0.891 | -0.36 (-0.93, 0.20) | 0.209 | 0.309 | -0.04 (-0.56,0.47) | 0.866 | 0.898 |
| Cannabis Risk Score | Pain intensity (between) | 0.27 (-0.95, 1.48) | 0.668 | 0.774 | 0.13 (-0.98, 1.23) | 0.821 | 0.862 | 0.13 (-1.11, 1.37) | 0.834 | 0.879 | -0.08 (-1.17, 1.01) | 0.886 | 0.913 |
| Cannabis Risk Score | Pain intensity (within) | 0.49 (-0.28, 1.26) | 0.214 | 0.318 | 0.56 (-0.17, 1.28) | 0.132 | 0.225 | 0.57 (-0.22, 1.36) | 0.157 | 0.247 | 0.49 (-0.24, 1.22) | 0.185 | 0.280 |
| Cannabis Risk Score | Pain limitations (between) | 0.16 (-0.60, 0.92) | 0.679 | 0.774 | 0.28 (-0.42, 0.98) | 0.436 | 0.555 | 0.13 (-0.64, 0.90) | 0.745 | 0.813 | 0.30 (-0.39, 0.99) | 0.398 | 0.522 |
| Cannabis Risk Score | Pain limitations (within) | 0.12 (-0.41, 0.65) | 0.665 | 0.774 | 0.34 (-0.15, 0.84) | 0.174 | 0.292 | 0.04 (-0.51, 0.58) | 0.893 | 0.915 | 0.31 (-0.18, 0.81) | 0.217 | 0.317 |
| FDR = False Discovery Rate. Bold indicates significance after FDR p-value adjustment.  *Between-subject estimates are the baseline score, indicating if baseline pain was associated with clinical outcomes across the three-month follow-up (level 2 predictor). Within-subject estimates are baseline-centred indicating if a fluctuation from the baseline pain score was associated with the clinical outcome (level 1 predictor). | | | | | | | | | | | | | |

| **Supplementary Table 7.** Multi-level linear mixed effects regression of serious pain frequency on depressive symptoms. | | | | | | | | |
| --- | --- | --- | --- | --- | --- | --- | --- | --- |
|  | **Unadjusted: Unimputed** | | **Unadjusted: Imputed** | | **Adjusted: Unimputed** | | **Adjusted: Imputed** | |
| **Variable** | **Estimate (95%CI)** | **p-value** | **Estimate (95%CI)** | **p-value** | **Estimate (95%CI)** | **p-value** | **Estimate (95%CI)** | **p-value** |
| Serious pain frequency (between) | 1.98 (1.57, 2.40) | <0.001 | 2.40 (2.01, 2.78) | <0.001 | 1.85 (1.45, 2.25) | <0.001 | 2.21 (1.86, 2.57) | <0.001 |
| Serious pain frequency (within) | 1.71 (1.16, 2.26) | <0.001 | 1.83 (1.39, 2.27) | <0.001 | 1.25 (0.74, 1.77) | <0.001 | 1.30 (0.89, 1.70) | <0.001 |
| Sex - Male | - | - | - | - | -2.41 (-3.12, -1.70) | <0.001 | -2.36 (-3.00, -1.72) | <0.001 |
| Age (years) | - | - | - | - | 0.16 (0.05, 0.26) | 0.003 | 0.17 (0.07, 0.26) | <0.001 |
| Anxiety only | - | - | - | - | -2.09 (-3.00, -1.19) | <0.001 | -2.37 (-3.18, -1.55) | <0.001 |
| Depression and anxiety | - | - | - | - | 0.18 (-0.68, 1.04) | 0.678 | 0.75 (-0.04, 1.54) | 0.064 |
| Other diagnosis | - | - | - | - | -2.62 (-3.56, -1.69) | <0.001 | -2.43 (-3.31, -1.56) | <0.001 |
| Time | - | - | - | - | -2.80 (-3.26, -2.34) | <0.001 | -2.65 (-3.03, -2.27) | <0.001 |
| **Random effects** | **Variance** | **SD** | **Variance** | **SD** | **Variance** | **SD** | **Variance** | **SD** |
| Centres | 0.39 | 0.63 | 0.63 | 0.80 | 0.17 | 0.41 | 0.29 | 0.54 |
| Participants within centres | 19.14 | 4.38 | 21.40 | 4.63 | 17.77 | 4.22 | 19.02 | 4.36 |
| Residual | 22.53 | 4.75 | 22.34 | 4.73 | 18.32 | 4.28 | 18.51 | 4.30 |
| For adjusted models, the coefficient for male and in reference to females. For diagnosis, the reference is depression only. | | | | | | | | |

| **Supplementary Table 8.** Multi-level linear mixed effects regression of pain intensity on depressive symptoms. | | | | | | | | |
| --- | --- | --- | --- | --- | --- | --- | --- | --- |
|  | **Unadjusted: Unimputed** | | **Unadjusted: Imputed** | | **Adjusted: Unimputed** | | **Adjusted: Imputed** | |
| **Variable** | **Estimate (95%CI)** | **p-value** | **Estimate (95%CI)** | **p-value** | **Estimate (95%CI)** | **p-value** | **Estimate (95%CI)** | **p-value** |
| Pain intensity (between) | 3.13 (2.57, 3.68) | <0.001 | 3.67 (3.16,4.19) | <0.001 | 2.83 (2.29, 3.37) | <0.001 | 3.28 (2.79, 3.76) | <0.001 |
| Pain intensity (within) | 2.61 (1.90, 3.32) | <0.001 | 2.57 (1.99,3.15) | <0.001 | 2.11 (1.45, 2.78) | <0.001 | 1.86 (1.33, 2.40) | <0.001 |
| Sex - Male | - | - | - | - | -2.31 (-3.02, -1.61) | <0.001 | -2.23 (-2.86, -1.59) | <0.001 |
| Age (years) | - | - | - | - | 0.12 (0.02, 0.23) | 0.019 | 0.13 (0.04, 0.23) | 0.004 |
| Anxiety only | - | - | - | - | -1.85 (-2.75, -0.95) | <0.001 | -2.18 (-2.99, -1.36) | <0.001 |
| Depression and anxiety | - | - | - | - | 0.24 (-0.61, 1.09) | 0.573 | 0.78 (0.00, 1.57) | 0.051 |
| Other diagnosis | - | - | - | - | -2.44 (-3.36, -1.51) | <0.001 | -2.27 (-3.14, -1.40) | <0.001 |
| Time | - | - | - | - | -2.82 (-3.28, -2.37) | <0.001 | -2.69 (-3.06, -2.31) | <0.001 |
| **Random effects** | **Variance** | **SD** | **Variance** | **SD** | **Variance** | **SD** | **Variance** | **SD** |
| Centres | 0.38 | 0.62 | 0.58 | 0.76 | 0.18 | 0.43 | 0.28 | 0.53 |
| Participants within centres | 18.42 | 4.29 | 20.62 | 4.54 | 17.46 | 4.18 | 18.62 | 4.32 |
| Residual | 22.23 | 4.71 | 22.12 | 4.70 | 18.01 | 4.24 | 18.34 | 4.28 |
| For adjusted models, the coefficient for male and in reference to females. For diagnosis, the reference is depression only. | | | | | | | | |

| **Supplementary Table 9.** Multi-level linear mixed effects regression of pain limitations on depressive symptoms. | | | | | | | | |
| --- | --- | --- | --- | --- | --- | --- | --- | --- |
|  | **Unadjusted: Unimputed** | | **Unadjusted: Imputed** | | **Adjusted: Unimputed** | | **Adjusted: Imputed** | |
| **Variable** | **Estimate (95%CI)** | **p-value** | **Estimate (95%CI)** | **p-value** | **Estimate (95%CI)** | **p-value** | **Estimate (95%CI)** | **p-value** |
| Pain limitations (between) | 2.02 (1.66, 2.39) | <0.001 | 2.47 (2.13, 2.81) | <0.001 | 1.78 (1.43, 2.13) | <0.001 | 2.20 (1.88, 2.52) | <0.001 |
| Pain limitations (within) | 1.99 (1.51, 2.47) | <0.001 | 1.99 (1.60, 2.38) | <0.001 | 1.41 (0.95, 1.86) | <0.001 | 1.47 (1.10, 1.83) | <0.001 |
| Sex - Male | - | - | - | - | -2.23 (-2.95, -1.52) | <0.001 | -2.12 (-2.76, -1.49) | <0.001 |
| Age (years) | - | - | - | - | 0.15 (0.04, 0.25) | 0.006 | 0.16 (0.06, 0.25) | 0.001 |
| Anxiety only | - | - | - | - | -1.93 (-2.83, -1.03) | <0.001 | -2.20 (-3.01, -1.39) | <0.001 |
| Depression and anxiety | - | - | - | - | 0.25 (-0.60, 1.10) | 0.558 | 0.77 (-0.01, 1.55) | 0.054 |
| Other diagnosis | - | - | - | - | -2.42 (-3.34, -1.49) | <0.001 | -2.25 (-3.11, -1.38) | <0.001 |
| Time | - | - | - | - | -2.73 (-3.19, -2.27) | <0.001 | -2.61 (-2.99, -2.24) | <0.001 |
| **Random effects** | **Variance** | **SD** | **Variance** | **SD** | **Variance** | **SD** | **Variance** | **SD** |
| Centres | 0.34 | 0.58 | 0.55 | 0.74 | 0.17 | 0.42 | 0.28 | 0.53 |
| Participants within centres | 19.35 | 4.40 | 21.19 | 4.60 | 18.10 | 4.25 | 19.03 | 4.36 |
| Residual | 21.53 | 4.64 | 21.44 | 4.63 | 17.75 | 4.21 | 17.91 | 4.23 |
| For adjusted models, the coefficient for male and in reference to females. For diagnosis, the reference is depression only. | | | | | | | | |

| **Supplementary Table 10.** Multi-level linear mixed effects regression of multiple pain predictors on depressive symptoms. | | | | | | | | | | | |
| --- | --- | --- | --- | --- | --- | --- | --- | --- | --- | --- | --- |
|  | | **Unadjusted: Unimputed** | | | | **Unadjusted: Imputed** | | **Adjusted: Unimputed** | | **Adjusted: Imputed** | |
| **Variable** | | **Estimate (95%CI)** | | **p-value** | | **Estimate (95%CI)** | **p-value** | **Estimate (95%CI)** | **p-value** | **Estimate (95%CI)** | **p-value** |
| Serious pain frequency (between) | | 0.34 (-0.26,0.94) | | 0.264 | | 0.39 (-0.18,0.96) | 0.184 | 0.50 (-0.08, 1.09) | 0.090 | 0.56 (0.02, 1.09) | 0.042 |
| Serious pain frequency (within) | | 0.51 (-0.12,1.15) | | 0.114 | | 0.68 (0.17,1.19) | 0.009 | 0.36 (-0.24, 0.95) | 0.239 | 0.46 (-0.01, 0.93) | 0.054 |
| Pain intensity (between) | | 1.73 (0.84,2.62) | | <0.001 | | 1.82 (0.98,2.66) | <0.001 | 1.49 (0.62, 2.37) | 0.001 | 1.50 (0.71, 2.28) | <0.001 |
| Pain intensity (within) | | 1.16 (0.28,2.04) | | 0.010 | | 1.02 (0.31,1.73) | 0.005 | 1.19 (0.37, 2.01) | 0.004 | 0.71 (0.05, 1.36) | 0.034 |
| Pain limitations (between) | | 0.99 (0.43,1.55) | | 0.001 | | 1.33 (0.80,1.87) | <0.001 | 0.77 (0.23, 1.31) | 0.005 | 1.13 (0.63, 1.63) | <0.001 |
| Pain limitations (within) | | 1.30 (0.69,1.90) | | <0.001 | | 1.32 (0.84,1.80) | <0.001 | 0.77 (0.20, 1.34) | 0.008 | 0.99 (0.54, 1.43) | <0.001 |
| Sex - Male | | - | | - | | - | - | -2.23 (-2.94, -1.53) | <0.001 | -2.11 (-2.74, -1.49) | <0.001 |
| Age (years) | | - | | - | | - | - | 0.14 (0.04, 0.24) | 0.009 | 0.15 (0.06, 0.24) | 0.001 |
| Anxiety only | | - | | - | | - | - | -1.86 (-2.75, -0.96) | <0.001 | -2.15 (-2.96, -1.34) | <0.001 |
| Depression and anxiety | | - | | - | | - | - | 0.26 (-0.58, 1.11) | 0.540 | 0.77 (0.00, 1.55) | 0.052 |
| Other diagnosis | | - | | - | | - | - | -2.44 (-3.37, -1.52) | <0.001 | -2.31 (-3.17, -1.44) | <0.001 |
| Time | | - | | - | | - | - | -2.71 (-3.17, -2.26) | <0.001 | -2.58 (-2.96, -2.21) | <0.001 |
| **Random effects** | | **Variance** | | **SD** | | **Variance** | **SD** | **Variance** | **SD** | **Variance** | **SD** |
| Centres | | 0.38 | | 0.62 | | 0.58 | 0.76 | 0.19 | 0.44 | 0.31 | 0.56 |
| Participants within centres | | 18.28 | | 4.28 | | 19.84 | 4.45 | 17.19 | 4.15 | 17.95 | 4.24 |
| Residual | | 21.71 | | 4.66 | | 21.70 | 4.66 | 17.86 | 4.23 | 18.15 | 4.26 |
| For adjusted models, the coefficient for male and in reference to females. For diagnosis, the reference is depression only. | | | | | | | | | | | |
| **Supplementary Table 11.** Multi-level linear mixed effects regression of serious pain frequency on anxiety symptoms. | | | | | | | | | | | |
|  | **Unadjusted: Unimputed** | | | | **Unadjusted: Imputed** | | | **Adjusted: Unimputed** | | **Adjusted: Imputed** | |
| **Variable** | **Estimate (95%CI)** | | **p-value** | | **Estimate (95%CI)** | | **p-value** | **Estimate (95%CI)** | **p-value** | **Estimate (95%CI)** | **p-value** |
| Serious pain frequency (between) | 1.74 (1.39, 2.10) | | <0.001 | | 2.03 (1.71, 2.34) | | <0.001 | 1.61 (1.27, 1.96) | <0.001 | 1.91 (1.61, 2.21) | <0.001 |
| Serious pain frequency (within) | 1.49 (1.02, 1.95) | | <0.001 | | 1.65 (1.28, 2.03) | | <0.001 | 1.16 (0.72, 1.60) | <0.001 | 1.25 (0.90, 1.61) | <0.001 |
| Sex - Male | - | | - | | - | | - | -2.14 (-2.76, -1.52) | <0.001 | -2.07 (-2.61, -1.54) | <0.001 |
| Age (years) | - | | - | | - | | - | 0.08 (-0.01, 0.17) | 0.073 | 0.13 (0.05, 0.20) | 0.001 |
| Anxiety only | - | | - | | - | | - | 0.51 (-0.27, 1.30) | 0.198 | 0.10 (-0.60, 0.80) | 0.782 |
| Depression and anxiety | - | | - | | - | | - | 1.27 (0.53, 2.01) | 0.001 | 1.65 (0.98, 2.33) | <0.001 |
| Other diagnosis | - | | - | | - | | - | -0.82 (-1.63, -0.01) | 0.048 | -0.53 (-1.28, 0.21) | 0.161 |
| Time | - | | - | | - | | - | -2.17 (-2.56, -1.77) | <0.001 | -2.24 (-2.57, -1.90) | <0.001 |
| **Random effects** | **Variance** | | **SD** | | **Variance** | | **SD** | **Variance** | **SD** | **Variance** | **SD** |
| Centres | 0.36 | | 0.60 | | 0.54 | | 0.73 | 0.18 | 0.42 | 0.21 | 0.45 |
| Participants within centres | 13.70 | | 3.70 | | 12.95 | | 3.60 | 13.48 | 3.67 | 12.16 | 3.49 |
| Residual | 16.28 | | 4.04 | | 16.75 | | 4.09 | 13.67 | 3.70 | 14.33 | 3.79 |
| For adjusted models, the coefficient for male and in reference to females. For diagnosis, the reference is depression only. | | | | | | | | | | | |

| **Supplementary Table 12.** Multi-level linear mixed effects regression of pain intensity on anxiety symptoms. | | | | | | | | |
| --- | --- | --- | --- | --- | --- | --- | --- | --- |
|  | **Unadjusted: Unimputed** | | **Unadjusted: Imputed** | | **Adjusted: Unimputed** | | **Adjusted: Imputed** | |
| **Variable** | **Estimate (95%CI)** | **p-value** | **Estimate (95%CI)** | **p-value** | **Estimate (95%CI)** | **p-value** | **Estimate (95%CI)** | **p-value** |
| Pain intensity (between) | 2.54 (2.06, 3.01) | <0.001 | 2.83 (2.41, 3.26) | <0.001 | 2.33 (1.85, 2.80) | <0.001 | 2.60 (2.19, 3.00) | <0.001 |
| Pain intensity (within) | 1.90 (1.30, 2.51) | <0.001 | 2.07 (1.58, 2.57) | <0.001 | 1.53 (0.96, 2.11) | <0.001 | 1.58 (1.12, 2.05) | <0.001 |
| Sex - Male | - | - | - | - | -2.07 (-2.69, -1.46) | <0.001 | -1.99 (-2.53, -1.46) | <0.001 |
| Age (years) | - | - | - | - | 0.05 (-0.04, 0.15) | 0.237 | 0.10 (0.02, 0.18) | 0.011 |
| Anxiety only | - | - | - | - | 0.70 (-0.08, 1.49) | 0.079 | 0.24 (-0.46, 0.94) | 0.499 |
| Depression and anxiety | - | - | - | - | 1.31 (0.57, 2.05) | 0.001 | 1.68 (1.01, 2.36) | <0.001 |
| Other diagnosis | - | - | - | - | -0.67 (-1.48, 0.14) | 0.103 | -0.39 (-1.14, 0.35) | 0.300 |
| Time | - | - | - | - | -2.21 (-2.61, -1.82) | <0.001 | -2.29 (-2.62, -1.96) | <0.001 |
| **Random effects** | **Variance** | **SD** | **Variance** | **SD** | **Variance** | **SD** | **Variance** | **SD** |
| Centres | 0.36 | 0.60 | 0.49 | 0.70 | 0.18 | 0.43 | 0.19 | 0.44 |
| Participants within centres | 13.50 | 3.67 | 12.92 | 3.60 | 13.33 | 3.65 | 12.25 | 3.50 |
| Residual | 16.27 | 4.03 | 16.70 | 4.09 | 13.67 | 3.70 | 14.29 | 3.78 |
| For adjusted models, the coefficient for male and in reference to females. For diagnosis, the reference is depression only. | | | | | | | | |

| **Supplementary Table 13.** Multi-level linear mixed effects regression of pain limitations on anxiety symptoms. | | | | | | | | |
| --- | --- | --- | --- | --- | --- | --- | --- | --- |
|  | **Unadjusted: Unimputed** | | **Unadjusted: Imputed** | | **Adjusted: Unimputed** | | **Adjusted: Imputed** | |
| **Variable** | **Estimate (95%CI)** | **p-value** | **Estimate (95%CI)** | **p-value** | **Estimate (95%CI)** | **p-value** | **Estimate (95%CI)** | **p-value** |
| Pain limitations (between) | 1.53 (1.22, 1.85) | <0.001 | 1.80 (1.53, 2.08) | <0.001 | 1.34 (1.03, 1.65) | <0.001 | 1.62 (1.35, 1.89) | <0.001 |
| Pain limitations (within) | 1.51 (1.10, 1.92) | <0.001 | 1.61 (1.27, 1.95) | <0.001 | 1.09 (0.69, 1.48) | <0.001 | 1.23 (0.91, 1.55) | <0.001 |
| Sex - Male | - | - | - | - | -2.03 (-2.66, -1.41) | <0.001 | -1.94 (-2.47, -1.40) | <0.001 |
| Age (years) | - | - | - | - | 0.07 (-0.02, 0.17) | 0.116 | 0.12 (0.04, 0.20) | 0.003 |
| Anxiety only | - | - | - | - | 0.60 (-0.19, 1.39) | 0.136 | 0.19 (-0.51, 0.89) | 0.595 |
| Depression and anxiety | - | - | - | - | 1.31 (0.57, 2.05) | 0.001 | 1.66 (0.99, 2.34) | <0.001 |
| Other diagnosis | - | - | - | - | -0.68 (-1.49, 0.14) | 0.103 | -0.38 (-1.13, 0.36) | 0.316 |
| Time | - | - | - | - | -2.13 (-2.53, -1.74) | <0.001 | -2.22 (-2.56, -1.89) | <0.001 |
| **Random effects** | **Variance** | **SD** | **Variance** | **SD** | **Variance** | **SD** | **Variance** | **SD** |
| Centres | 0.32 | 0.57 | 0.48 | 0.70 | 0.17 | 0.41 | 0.19 | 0.43 |
| Participants within centres | 14.16 | 3.76 | 13.37 | 3.66 | 13.91 | 3.73 | 12.66 | 3.56 |
| Residual | 15.92 | 3.99 | 16.39 | 4.05 | 13.54 | 3.68 | 14.10 | 3.76 |
| For adjusted models, the coefficient for male and in reference to females. For diagnosis, the reference is depression only. | | | | | | | | |

| **Supplementary Table 14.** Multi-level linear mixed effects regression of multiple pain predictors on anxiety symptoms. | | | | | | | | | | | |
| --- | --- | --- | --- | --- | --- | --- | --- | --- | --- | --- | --- |
|  | | **Unadjusted: Unimputed** | | | | **Unadjusted: Imputed** | | **Adjusted: Unimputed** | | **Adjusted: Imputed** | |
| **Variable** | | **Estimate (95%CI)** | | **p-value** | | **Estimate (95%CI)** | **p-value** | **Estimate (95%CI)** | **p-value** | **Estimate (95%CI)** | **p-value** |
| Serious pain frequency (between) | | 0.69 (0.17, 1.21) | | 0.009 | | 0.80 (0.32, 1.27) | 0.001 | 0.73 (0.22, 1.24) | 0.005 | 0.90 (0.45, 1.35) | <0.001 |
| Serious pain frequency (within) | | 0.71 (0.16, 1.25) | | 0.011 | | 0.84 (0.40, 1.27) | <0.001 | 0.59 (0.08, 1.11) | 0.025 | 0.65 (0.24, 1.06) | 0.002 |
| Pain intensity (between) | | 1.38 (0.61, 2.15) | | <0.001 | | 1.38 (0.68, 2.07) | <0.001 | 1.34 (0.58, 2.10) | 0.001 | 1.22 (0.56, 1.89) | <0.001 |
| Pain intensity (within) | | 0.64 (-0.12, 1.39) | | 0.097 | | 0.74 (0.13, 1.34) | 0.018 | 0.67 (-0.04, 1.38) | 0.066 | 0.55 (-0.02, 1.12) | 0.059 |
| Pain limitations (between) | | 0.46 (-0.02, 0.94) | | 0.059 | | 0.63 (0.19, 1.07) | 0.005 | 0.27 (-0.21, 0.74) | 0.272 | 0.47 (0.05, 0.89) | 0.030 |
| Pain limitations (within) | | 0.92 (0.40, 1.44) | | 0.001 | | 0.95 (0.53, 1.36) | <0.001 | 0.54 (0.05, 1.04) | 0.032 | 0.71 (0.32, 1.10) | <0.001 |
| Sex - Male | | - | | - | | - | - | -2.04 (-2.66, -1.43) | <0.001 | -1.94 (-2.46, -1.41) | <0.001 |
| Age (years) | | - | | - | | - | - | 0.07 (-0.02, 0.16) | 0.140 | 0.12 (0.04, 0.19) | 0.003 |
| Anxiety only | | - | | - | | - | - | 0.70 (-0.08, 1.48) | 0.079 | 0.27 (-0.43, 0.96) | 0.449 |
| Depression and anxiety | | - | | - | | - | - | 1.33 (0.59, 2.07) | <0.001 | 1.68 (1.01, 2.35) | <0.001 |
| Other diagnosis | | - | | - | | - | - | -0.70 (-1.50, 0.11) | 0.089 | -0.44 (-1.18, 0.30) | 0.244 |
| Time | | - | | - | | - | - | -2.11 (-2.51, -1.71) | <0.001 | -2.19 (-2.52, -1.86) | <0.001 |
| **Random effects** | | **Variance** | | **SD** | | **Variance** | **SD** | **Variance** | **SD** | **Variance** | **SD** |
| Centres | | 0.37 | | 0.61 | | 0.51 | 0.72 | 0.20 | 0.44 | 0.22 | 0.46 |
| Participants within centres | | 13.38 | | 3.66 | | 12.40 | 3.52 | 13.11 | 3.62 | 11.71 | 3.42 |
| Residual | | 15.92 | | 3.99 | | 16.43 | 4.05 | 13.57 | 3.68 | 14.20 | 3.77 |
| For adjusted models, the coefficient for male and in reference to females. For diagnosis, the reference is depression only. | | | | | | | | | | | |
| **Supplementary Table 15.** Multi-level linear mixed effects regression of serious pain frequency on suicidal ideations. | | | | | | | | | | | |
|  | **Unadjusted: Unimputed** | | | | **Unadjusted: Imputed** | | | **Adjusted: Unimputed** | | **Adjusted: Imputed** | |
| **Variable** | **Estimate (95%CI)** | | **p-value** | | **Estimate (95%CI)** | | **p-value** | **Estimate (95%CI)** | **p-value** | **Estimate (95%CI)** | **p-value** |
| Serious pain frequency (between) | 5.15 (3.88, 6.41) | | <0.001 | | 5.90 (4.74, 7.05) | | <0.001 | 5.14 (3.88, 6.39) | <0.001 | 5.58 (4.45, 6.71) | <0.001 |
| Serious pain frequency (within) | 2.42 (1.05, 3.79) | | 0.001 | | 3.28 (2.13, 4.44) | | <0.001 | 1.84 (0.46, 3.22) | 0.009 | 2.71 (1.56, 3.85) | <0.001 |
| Sex - Male | - | | - | | - | | - | -1.10 (-3.36, 1.17) | 0.343 | -2.11 (-4.14, -0.08) | 0.042 |
| Age (years) | - | | - | | - | | - | -0.08 (-0.41, 0.26) | 0.652 | -0.03 (-0.32, 0.26) | 0.826 |
| Anxiety only | - | | - | | - | | - | -5.61 (-8.25, -2.96) | <0.001 | -5.06 (-7.42, -2.69) | <0.001 |
| Depression and anxiety | - | | - | | - | | - | 1.07 (-1.40, 3.54) | 0.396 | 2.44 (0.15, 4.73) | 0.037 |
| Other diagnosis | - | | - | | - | | - | -3.08 (-5.81, -0.34) | 0.027 | -3.14 (-5.71, -0.56) | 0.017 |
| Time | - | | - | | - | | - | -3.57 (-4.78, -2.36) | <0.001 | -3.09 (-4.13, -2.05) | <0.001 |
| **Random effects** | **Variance** | | **SD** | | **Variance** | | **SD** | **Variance** | **SD** | **Variance** | **SD** |
| Centres | 3.90 | | 1.98 | | 3.11 | | 1.76 | 3.27 | 1.81 | 2.15 | 1.47 |
| Participants within centres | 239.42 | | 15.47 | | 239.05 | | 15.46 | 222.35 | 14.91 | 221.04 | 14.87 |
| Residual | 126.26 | | 11.24 | | 140.04 | | 11.83 | 121.63 | 11.03 | 136.71 | 11.69 |
| For adjusted models, the coefficient for male and in reference to females. For diagnosis, the reference is depression only. | | | | | | | | | | | |

| **Supplementary Table 16.** Multi-level linear mixed effects regression of pain intensity on suicidal ideations. | | | | | | | | |
| --- | --- | --- | --- | --- | --- | --- | --- | --- |
|  | **Unadjusted: Unimputed** | | **Unadjusted: Imputed** | | **Adjusted: Unimputed** | | **Adjusted: Imputed** | |
| **Variable** | **Estimate (95%CI)** | **p-value** | **Estimate (95%CI)** | **p-value** | **Estimate (95%CI)** | **p-value** | **Estimate (95%CI)** | **p-value** |
| Pain intensity (between) | 7.59 (5.87, 9.31) | <0.001 | 8.65 (7.08, 10.21) | <0.001 | 7.37 (5.64, 9.10) | <0.001 | 8.05 (6.52, 9.58) | <0.001 |
| Pain intensity (within) | 4.61 (2.84, 6.37) | <0.001 | 5.17 (3.65, 6.70) | <0.001 | 4.07 (2.30, 5.85) | <0.001 | 4.27 (2.76, 5.78) | <0.001 |
| Sex - Male | - | - | - | - | -0.88 (-3.14, 1.38) | 0.446 | -1.80 (-3.82, 0.23) | 0.083 |
| Age (years) | - | - | - | - | -0.16 (-0.50, 0.17) | 0.340 | -0.11 (-0.40, 0.18) | 0.466 |
| Anxiety only | - | - | - | - | -4.98 (-7.63, -2.34) | <0.001 | -4.59 (-6.95, -2.24) | <0.001 |
| Depression and anxiety | - | - | - | - | 1.19 (-1.26, 3.65) | 0.341 | 2.48 (0.20, 4.76) | 0.033 |
| Other diagnosis | - | - | - | - | -2.57 (-5.29, 0.15) | 0.064 | -2.70 (-5.27, -0.14) | 0.039 |
| Time | - | - | - | - | -3.57 (-4.76, -2.37) | <0.001 | -3.15 (-4.18, -2.12) | <0.001 |
| **Random effects** | **Variance** | **SD** | **Variance** | **SD** | **Variance** | **SD** | **Variance** | **SD** |
| Centres | 3.56 | 1.89 | 2.81 | 1.68 | 3.09 | 1.76 | 2.04 | 1.43 |
| Participants within centres | 238.49 | 15.44 | 237.69 | 15.42 | 223.06 | 14.94 | 221.28 | 14.88 |
| Residual | 124.07 | 11.14 | 138.01 | 11.75 | 119.52 | 10.93 | 134.89 | 11.61 |
| For adjusted models, the coefficient for male and in reference to females. For diagnosis, the reference is depression only. | | | | | | | | |

| **Supplementary Table 17.** Multi-level linear mixed effects regression of pain limitations on suicidal ideations. | | | | | | | | |
| --- | --- | --- | --- | --- | --- | --- | --- | --- |
|  | **Unadjusted: Unimputed** | | **Unadjusted: Imputed** | | **Adjusted: Unimputed** | | **Adjusted: Imputed** | |
| **Variable** | **Estimate (95%CI)** | **p-value** | **Estimate (95%CI)** | **p-value** | **Estimate (95%CI)** | **p-value** | **Estimate (95%CI)** | **p-value** |
| Pain limitations (between) | 4.72 (3.60, 5.84) | <0.001 | 5.78 (4.75, 6.81) | <0.001 | 4.59 (3.48, 5.71) | <0.001 | 5.39 (4.38, 6.39) | <0.001 |
| Pain limitations (within) | 3.26 (2.04, 4.47) | <0.001 | 3.77 (2.73, 4.81) | <0.001 | 2.51 (1.28, 3.74) | <0.001 | 3.08 (2.04, 4.12) | <0.001 |
| Sex - Male | - | - | - | - | -0.68 (-2.95, 1.60) | 0.559 | -1.55 (-3.58, 0.48) | 0.135 |
| Age (years) | - | - | - | - | -0.11 (-0.44, 0.23) | 0.524 | -0.06 (-0.35, 0.23) | 0.672 |
| Anxiety only | - | - | - | - | -5.26 (-7.90, -2.61) | <0.001 | -4.69 (-7.05, -2.34) | <0.001 |
| Depression and anxiety | - | - | - | - | 1.20 (-1.26, 3.66) | 0.338 | 2.47 (0.20, 4.74) | 0.033 |
| Other diagnosis | - | - | - | - | -2.64 (-5.37, 0.09) | 0.058 | -2.73 (-5.29, -0.18) | 0.036 |
| Time | - | - | - | - | -3.42 (-4.63, -2.21) | <0.001 | -3.02 (-4.05, -1.99) | <0.001 |
| **Random effects** | **Variance** | **SD** | **Variance** | **SD** | **Variance** | **SD** | **Variance** | **SD** |
| Centres | 3.18 | 1.78 | 2.48 | 1.58 | 2.76 | 1.66 | 1.82 | 1.35 |
| Participants within centres | 241.41 | 15.54 | 238.65 | 15.45 | 224.36 | 14.98 | 222.05 | 14.90 |
| Residual | 123.53 | 11.11 | 136.80 | 11.70 | 119.99 | 10.95 | 134.02 | 11.58 |
| For adjusted models, the coefficient for male and in reference to females. For diagnosis, the reference is depression only. | | | | | | | | |

| **Supplementary Table 18.** Multi-level linear mixed effects regression of multiple pain predictors on suicidal ideations. | | | | | | | | | | | |
| --- | --- | --- | --- | --- | --- | --- | --- | --- | --- | --- | --- |
|  | | **Unadjusted: Unimputed** | | | | **Unadjusted: Imputed** | | **Adjusted: Unimputed** | | **Adjusted: Imputed** | |
| **Variable** | | **Estimate (95%CI)** | | **p-value** | | **Estimate (95%CI)** | **p-value** | **Estimate (95%CI)** | **p-value** | **Estimate (95%CI)** | **p-value** |
| Serious pain frequency (between) | | 1.80 (-0.05, 3.65) | | 0.056 | | 1.61 (-0.12,3.34) | 0.068 | 2.08 (0.24, 3.93) | 0.027 | 1.75 (0.07, 3.44) | 0.042 |
| Serious pain frequency (within) | | 0.31 (-1.29, 1.90) | | 0.707 | | 1.07 (-0.26,2.40) | 0.114 | 0.05 (-1.55, 1.66) | 0.948 | 0.91 (-0.41, 2.22) | 0.177 |
| Pain intensity (between) | | 3.94 (1.18, 6.71) | | 0.005 | | 3.92 (1.37, 6.47) | 0.003 | 3.46 (0.69, 6.24) | 0.014 | 3.47 (0.98, 5.96) | 0.006 |
| Pain intensity (within) | | 2.56 (0.36, 4.75) | | 0.023 | | 2.45 (0.59, 4.31) | 0.010 | 2.71 (0.51, 4.92) | 0.016 | 2.00 (0.16, 3.84) | 0.033 |
| Pain limitations (between) | | 1.75 (0.03, 3.48) | | 0.046 | | 2.86 (1.23, 4.48) | 0.001 | 1.70 (-0.02, 3.43) | 0.053 | 2.61 (1.02, 4.19) | 0.001 |
| Pain limitations (within) | | 2.06 (0.54, 3.58) | | 0.008 | | 2.36 (1.09, 3.62) | <0.001 | 1.36 (-0.16, 2.89) | 0.080 | 1.90 (0.64, 3.15) | 0.003 |
| Sex - Male | | - | | - | | - | - | -0.71 (-2.96, 1.55) | 0.539 | -1.55 (-3.56, 0.47) | 0.132 |
| Age (years) | | - | | - | | - | - | -0.12 (-0.45, 0.21) | 0.483 | -0.07 (-0.36, 0.22) | 0.629 |
| Anxiety only | | - | | - | | - | - | -5.09 (-7.73, -2.45) | <0.001 | -4.60 (-6.95, -2.25) | <0.001 |
| Depression and anxiety | | - | | - | | - | - | 1.19 (-1.26, 3.64) | 0.340 | 2.45 (0.18, 4.72) | 0.034 |
| Other diagnosis | | - | | - | | - | - | -2.70 (-5.41, 0.02) | 0.052 | -2.84 (-5.39, -0.28) | 0.029 |
| Time | | - | | - | | - | - | -3.41 (-4.62, -2.20) | <0.001 | -2.95 (-3.99, -1.91) | <0.001 |
| **Random effects** | | **Variance** | | **SD** | | **Variance** | **SD** | **Variance** | **SD** | **Variance** | **SD** |
| Centres | | 3.77 | | 1.94 | | 2.91 | 1.71 | 3.19 | 1.79 | 2.15 | 1.46 |
| Participants within centres | | 235.85 | | 15.36 | | 231.46 | 15.21 | 219.79 | 14.83 | 215.74 | 14.69 |
| Residual | | 123.46 | | 11.11 | | 137.52 | 11.73 | 119.54 | 10.93 | 134.83 | 11.61 |
| For adjusted models, the coefficient for male and in reference to females. For diagnosis, the reference is depression only. | | | | | | | | | | | |
| **Supplementary Table 19.** Multi-level linear mixed effects regression of serious pain frequency on social and occupational functioning. | | | | | | | | | | | |
|  | **Unadjusted: Unimputed** | | | | **Unadjusted: Imputed** | | | **Adjusted: Unimputed** | | **Adjusted: Imputed** | |
| **Variable** | **Estimate (95%CI)** | | **p-value** | | **Estimate (95%CI)** | | **p-value** | **Estimate (95%CI)** | **p-value** | **Estimate (95%CI)** | **p-value** |
| Serious pain frequency (between) | -1.46 (-2.10, -0.81) | | <0.001 | | -1.83 (-2.42, -1.25) | | <0.001 | -1.21 (-1.85, -0.58) | <0.001 | -1.60 (-2.17, -1.02) | <0.001 |
| Serious pain frequency (within) | -1.47 (-2.28, -0.66) | | <0.001 | | -1.91 (-2.59, -1.23) | | <0.001 | -0.75 (-1.50, -0.01) | 0.047 | -1.19 (-1.82, -0.55) | <0.001 |
| Sex - Male | - | | - | | - | | - | -0.36 (-1.51, 0.78) | 0.531 | -0.34 (-1.36, 0.68) | 0.512 |
| Age (years) | - | | - | | - | | - | -0.13 (-0.30, 0.04) | 0.125 | -0.24 (-0.38, -0.09) | 0.002 |
| Anxiety only | - | | - | | - | | - | 3.78 (2.39, 5.17) | <0.001 | 3.71 (2.43, 4.99) | <0.001 |
| Depression and anxiety | - | | - | | - | | - | 1.16 (-0.14, 2.47) | 0.080 | 0.59 (-0.65, 1.83) | 0.351 |
| Other diagnosis | - | | - | | - | | - | 2.61 (1.17, 4.05) | <0.001 | 1.96 (0.58, 3.34) | 0.005 |
| Time | - | | - | | - | | - | 4.24 (3.58, 4.90) | <0.001 | 4.17 (3.57, 4.76) | <0.001 |
| **Random effects** | **Variance** | | **SD** | | **Variance** | | **SD** | **Variance** | **SD** | **Variance** | **SD** |
| Centres | 1.81 | | 1.35 | | 0.67 | | 0.82 | 1.83 | 1.35 | 1.23 | 1.11 |
| Participants within centres | 50.23 | | 7.09 | | 48.73 | | 6.98 | 52.53 | 7.25 | 49.65 | 7.05 |
| Residual | 46.44 | | 6.81 | | 53.56 | | 7.32 | 37.07 | 6.09 | 44.55 | 6.67 |
| For adjusted models, the coefficient for male and in reference to females. For diagnosis, the reference is depression only. | | | | | | | | | | | |

| **Supplementary Table 20.** Multi-level linear mixed effects regression of pain intensity on social and occupational functioning. | | | | | | | | |
| --- | --- | --- | --- | --- | --- | --- | --- | --- |
|  | **Unadjusted: Unimputed** | | **Unadjusted: Imputed** | | **Adjusted: Unimputed** | | **Adjusted: Imputed** | |
| **Variable** | **Estimate (95%CI)** | **p-value** | **Estimate (95%CI)** | **p-value** | **Estimate (95%CI)** | **p-value** | **Estimate (95%CI)** | **p-value** |
| Pain intensity (between) | -2.34 (-3.21, -1.46) | <0.001 | -3.03 (-3.82, -2.24) | <0.001 | -1.87 (-2.75, -1.00) | <0.001 | -2.64 (-3.42, -1.87) | <0.001 |
| Pain intensity (within) | -2.13 (-3.17, -1.08) | <0.001 | -2.64 (-3.54, -1.74) | <0.001 | -1.41 (-2.38, -0.44) | 0.005 | -1.81 (-2.65, -0.97) | <0.001 |
| Sex - Male | - | - | - | - | -0.43 (-1.57, 0.71) | 0.461 | -0.46 (-1.48, 0.56) | 0.376 |
| Age (years) | - | - | - | - | -0.11 (-0.28, 0.06) | 0.198 | -0.21 (-0.36, -0.06) | 0.005 |
| Anxiety only | - | - | - | - | 3.62 (2.23, 5.01) | <0.001 | 3.53 (2.25, 4.81) | <0.001 |
| Depression and anxiety | - | - | - | - | 1.13 (-0.17, 2.43) | 0.089 | 0.58 (-0.66, 1.81) | 0.362 |
| Other diagnosis | - | - | - | - | 2.48 (1.05, 3.91) | 0.001 | 1.84 (0.46, 3.21) | 0.009 |
| Time | - | - | - | - | 4.25 (3.59, 4.90) | <0.001 | 4.20 (3.61, 4.78) | <0.001 |
| **Random effects** | **Variance** | **SD** | **Variance** | **SD** | **Variance** | **SD** | **Variance** | **SD** |
| Centres | 1.78 | 1.33 | 0.58 | 0.76 | 1.79 | 1.34 | 1.21 | 1.79 |
| Participants within centres | 50.12 | 7.08 | 48.28 | 6.95 | 52.46 | 7.24 | 49.26 | 52.46 |
| Residual | 46.11 | 6.79 | 53.17 | 7.29 | 36.86 | 6.07 | 44.25 | 36.86 |
| For adjusted models, the coefficient for male and in reference to females. For diagnosis, the reference is depression only. | | | | | | | | |

| **Supplementary Table 21.** Multi-level linear mixed effects regression of pain limitations on social and occupational functioning. | | | | | | | | |
| --- | --- | --- | --- | --- | --- | --- | --- | --- |
|  | **Unadjusted: Unimputed** | | **Unadjusted: Imputed** | | **Adjusted: Unimputed** | | **Adjusted: Imputed** | |
| **Variable** | **Estimate (95%CI)** | **p-value** | **Estimate (95%CI)** | **p-value** | **Estimate (95%CI)** | **p-value** | **Estimate (95%CI)** | **p-value** |
| Pain limitations (between) | -1.53 (-2.10, -0.96) | <0.001 | -1.98 (-2.50, -1.46) | <0.001 | -1.31 (-1.88, -0.75) | <0.001 | -1.77 (-2.27, -1.26) | <0.001 |
| Pain limitations (within) | -2.06 (-2.77, -1.35) | <0.001 | -2.15 (-2.76, -1.53) | <0.001 | -1.25 (-1.92, -0.59) | <0.001 | -1.49 (-2.06, -0.91) | <0.001 |
| Sex - Male | - | - | - | - | -0.51 (-1.65, 0.64) | 0.383 | -0.54 (-1.56, 0.48) | 0.298 |
| Age (years) | - | - | - | - | -0.13 (-0.29, 0.04) | 0.143 | -0.23 (-0.38, -0.08) | 0.002 |
| Anxiety only | - | - | - | - | 3.64 (2.25, 5.02) | <0.001 | 3.54 (2.27, 4.82) | <0.001 |
| Depression and anxiety | - | - | - | - | 1.11 (-0.19, 2.41) | 0.094 | 0.59 (-0.65, 1.82) | 0.351 |
| Other diagnosis | - | - | - | - | 2.47 (1.04, 3.90) | 0.001 | 1.84 (0.47, 3.21) | 0.009 |
| Time | - | - | - | - | 4.13 (3.47, 4.79) | <0.001 | 4.12 (3.53, 4.70) | <0.001 |
| **Random effects** | **Variance** | **SD** | **Variance** | **SD** | **Variance** | **SD** | **Variance** | **SD** |
| Centres | 1.87 | 1.37 | 0.74 | 0.86 | 1.84 | 1.36 | 1.31 | 1.14 |
| Participants within centres | 51.17 | 7.15 | 49.48 | 7.03 | 52.72 | 7.26 | 49.93 | 7.07 |
| Residual | 44.92 | 6.70 | 52.10 | 7.22 | 36.46 | 6.04 | 43.69 | 6.61 |
| For adjusted models, the coefficient for male and in reference to females. For diagnosis, the reference is depression only. | | | | | | | | |

| **Supplementary Table 22.** Multi-level linear mixed effects regression of multiple pain predictors on social and occupational functioning. | | | | | | | | | | | |
| --- | --- | --- | --- | --- | --- | --- | --- | --- | --- | --- | --- |
|  | | **Unadjusted: Unimputed** | | | | **Unadjusted: Imputed** | | **Adjusted: Unimputed** | | **Adjusted: Imputed** | |
| **Variable** | | **Estimate (95%CI)** | | **p-value** | | **Estimate (95%CI)** | **p-value** | **Estimate (95%CI)** | **p-value** | **Estimate (95%CI)** | **p-value** |
| Serious pain frequency (between) | | -0.19 (-1.15, 0.76) | | 0.691 | | -0.01 (-0.90, 0.88) | 0.986 | -0.20 (-1.14, 0.74) | 0.681 | -0.01 (-0.87, 0.86) | 0.990 |
| Serious pain frequency (within) | | -0.29 (-1.23, 0.66) | | 0.549 | | -0.68 (-1.47, 0.11) | 0.093 | 0.02 (-0.85, 0.89) | 0.958 | -0.29 (-1.03, 0.44) | 0.432 |
| Pain intensity (between) | | -1.32 (-2.74, 0.10) | | 0.069 | | -1.88 (-3.18, -0.57) | 0.005 | -0.78 (-2.19, 0.63) | 0.280 | -1.53 (-2.80, -0.26) | 0.018 |
| Pain intensity (within) | | -0.52 (-1.81, 0.78) | | 0.436 | | -1.06 (-2.16, 0.04) | 0.060 | -0.53 (-1.73, 0.67) | 0.386 | -0.76 (-1.78, 0.27) | 0.147 |
| Pain limitations (between) | | -0.78 (-1.67, 0.11) | | 0.086 | | -1.05 (-1.88, -0.21) | 0.014 | -0.82 (-1.70, 0.05) | 0.065 | -1.01 (-1.82, -0.20) | 0.014 |
| Pain limitations (within) | | -1.72 (-2.61, -0.82) | | <0.001 | | -1.47 (-2.22, -0.73) | <0.001 | -1.05 (-1.88, -0.21) | 0.014 | -1.08 (-1.78, -0.38) | 0.002 |
| Sex - Male | | - | | - | | - | - | -0.51 (-1.65, 0.64) | 0.385 | -0.57 (-1.58, 0.45) | 0.275 |
| Age (years) | | - | | - | | - | - | -0.12 (-0.29, 0.05) | 0.159 | -0.22 (-0.37, -0.07) | 0.003 |
| Anxiety only | | - | | - | | - | - | 3.59 (2.20, 4.98) | <0.001 | 3.47 (2.20, 4.75) | <0.001 |
| Depression and anxiety | | - | | - | | - | - | 1.10 (-0.20, 2.40) | 0.096 | 0.58 (-0.66, 1.81) | 0.359 |
| Other diagnosis | | - | | - | | - | - | 2.47 (1.04, 3.90) | 0.001 | 1.82 (0.45, 3.19) | 0.009 |
| Time | | - | | - | | - | - | 4.13 (3.47, 4.80) | <0.001 | 4.09 (3.50, 4.68) | <0.001 |
| **Random effects** | | **Variance** | | **SD** | | **Variance** | **SD** | **Variance** | **SD** | **Variance** | **SD** |
| Centres | | 1.81 | | 1.34 | | 0.69 | 0.83 | 1.79 | 1.34 | 1.26 | 1.12 |
| Participants within centres | | 50.62 | | 7.11 | | 48.23 | 6.95 | 52.63 | 7.25 | 49.20 | 7.01 |
| Residual | | 45.18 | | 6.72 | | 52.43 | 7.24 | 36.57 | 6.05 | 43.94 | 6.63 |
| For adjusted models, the coefficient for male and in reference to females. For diagnosis, the reference is depression only. | | | | | | | | | | | |
| **Supplementary Table 23.** Multi-level linear mixed effects regression of serious pain frequency on tobacco use risk scores. | | | | | | | | | | | |
|  | **Unadjusted: Unimputed** | | | | **Unadjusted: Imputed** | | | **Adjusted: Unimputed** | | **Adjusted: Imputed** | |
| **Variable** | **Estimate (95%CI)** | | **p-value** | | **Estimate (95%CI)** | | **p-value** | **Estimate (95%CI)** | **p-value** | **Estimate (95%CI)** | **p-value** |
| Serious pain frequency (between) | 1.03 (0.47, 1.59) | | <0.001 | | 1.23 (0.73,1.72) | | <0.001 | 1.08 (0.52, 1.64) | <0.001 | 1.23 (0.75, 1.71) | <0.001 |
| Serious pain frequency (within) | 0.13 (-0.23, 0.50) | | 0.482 | | 0.30 (-0.08, 0.68) | | 0.117 | 0.12 (-0.26, 0.50) | 0.530 | 0.29 (-0.09, 0.67) | 0.132 |
| Sex - Male | - | | - | | - | | - | 1.13 (0.11, 2.14) | 0.030 | 0.56 (-0.31, 1.44) | 0.206 |
| Age (years) | - | | - | | - | | - | 0.53 (0.38, 0.68) | <0.001 | 0.51 (0.39, 0.63) | <0.001 |
| Anxiety only | - | | - | | - | | - | -0.93 (-1.78, -0.07) | 0.033 | -1.15 (-1.97, -0.33) | 0.006 |
| Depression and anxiety | - | | - | | - | | - | -0.64 (-1.41, 0.12) | 0.100 | -0.57 (-1.37, 0.22) | 0.159 |
| Other diagnosis | - | | - | | - | | - | 0.04 (-0.85, 0.92) | 0.936 | 0.08 (-0.84, 1.00) | 0.857 |
| Time | - | | - | | - | | - | -0.02 (-0.34, 0.30) | 0.883 | -0.14 (-0.47, 0.20) | 0.427 |
| **Random effects** | **Variance** | | **SD** | | **Variance** | | **SD** | **Variance** | **SD** | **Variance** | **SD** |
| Centres | 2.78 | | 1.67 | | 2.76 | | 1.66 | 2.57 | 1.60 | 2.35 | 1.53 |
| Participants within centres | 59.59 | | 7.72 | | 52.38 | | 7.24 | 55.82 | 7.47 | 48.23 | 6.95 |
| Residual | 7.66 | | 2.77 | | 13.46 | | 3.67 | 7.69 | 2.77 | 13.64 | 3.69 |
| For adjusted models, the coefficient for male and in reference to females. For diagnosis, the reference is depression only. | | | | | | | | | | | |

| **Supplementary Table 24.** Multi-level linear mixed effects regression of pain intensity on tobacco use risk scores. | | | | | | | | |
| --- | --- | --- | --- | --- | --- | --- | --- | --- |
|  | **Unadjusted: Unimputed** | | **Unadjusted: Imputed** | | **Adjusted: Unimputed** | | **Adjusted: Imputed** | |
| **Variable** | **Estimate (95%CI)** | **p-value** | **Estimate (95%CI)** | **p-value** | **Estimate (95%CI)** | **p-value** | **Estimate (95%CI)** | **p-value** |
| Pain intensity (between) | 1.40 (0.63, 2.17) | <0.001 | 1.61 (0.94, 2.28) | <0.001 | 1.24 (0.47, 2.01) | 0.002 | 1.51 (0.85, 2.16) | <0.001 |
| Pain intensity (within) | 0.71 (0.23, 1.19) | 0.004 | 0.99 (0.49, 1.49) | <0.001 | 0.72 (0.23, 1.21) | 0.004 | 0.96 (0.45, 1.46) | <0.001 |
| Sex - Male | - | - | - | - | 1.15 (0.13,2.17) | 0.027 | 0.62 (-0.25, 1.49) | 0.165 |
| Age (years) | - | - | - | - | 0.52 (0.37, 0.67) | <0.001 | 0.50 (0.38, 0.62) | <0.001 |
| Anxiety only | - | - | - | - | -0.85 (-1.70, 0.00) | 0.050 | -1.07 (-1.89, -0.25) | 0.011 |
| Depression and anxiety | - | - | - | - | -0.63 (-1.39, 0.14) | 0.108 | -0.58 (-1.38, 0.21) | 0.149 |
| Other diagnosis | - | - | - | - | 0.12 (-0.76, 1.00) | 0.795 | 0.19 (-0.73, 1.11) | 0.685 |
| Time | - | - | - | - | -0.01 (-0.32, 0.31) | 0.975 | -0.11 (-0.44, 0.22) | 0.508 |
| **Random effects** | **Variance** | **SD** | **Variance** | **SD** | **Variance** | **SD** | **Variance** | **SD** |
| Centres | 2.77 | 1.66 | 2.76 | 1.66 | 2.60 | 1.61 | 2.39 | 2.60 |
| Participants within centres | 59.62 | 7.72 | 52.28 | 7.23 | 56.05 | 7.49 | 48.31 | 56.05 |
| Residual | 7.59 | 2.75 | 13.40 | 3.66 | 7.63 | 2.76 | 13.58 | 7.63 |
| For adjusted models, the coefficient for male and in reference to females. For diagnosis, the reference is depression only. | | | | | | | | |

| **Supplementary Table 25.** Multi-level linear mixed effects regression of pain limitations on tobacco use risk scores. | | | | | | | | |
| --- | --- | --- | --- | --- | --- | --- | --- | --- |
|  | **Unadjusted: Unimputed** | | **Unadjusted: Imputed** | | **Adjusted: Unimputed** | | **Adjusted: Imputed** | |
| **Variable** | **Estimate (95%CI)** | **p-value** | **Estimate (95%CI)** | **p-value** | **Estimate (95%CI)** | **p-value** | **Estimate (95%CI)** | **p-value** |
| Pain limitations (between) | 0.83 (0.33, 1.33) | 0.001 | 1.08 (0.64,1.52) | <0.001 | 0.74 (0.24, 1.24) | 0.004 | 1.06 (0.63, 1.48) | <0.001 |
| Pain limitations (within) | 0.06 (-0.27, 0.39) | 0.713 | 0.12 (-0.23, 0.46) | 0.507 | 0.03 (-0.31, 0.37) | 0.876 | 0.08 (-0.26, 0.43) | 0.633 |
| Sex - Male | - | - | - | - | 1.17 (0.15, 2.19) | 0.025 | 0.65 (-0.22, 1.53) | 0.143 |
| Age (years) | - | - | - | - | 0.53 (0.38, 0.68) | <0.001 | 0.50 (0.38, 0.63) | <0.001 |
| Anxiety only | - | - | - | - | -0.92 (-1.77, -0.06) | 0.036 | -1.13 (-1.95, -0.31) | 0.007 |
| Depression and anxiety | - | - | - | - | -0.64 (-1.41, 0.13) | 0.104 | -0.56 (-1.35, 0.24) | 0.168 |
| Other diagnosis | - | - | - | - | 0.07 (-0.81, 0.96) | 0.869 | 0.14 (-0.78, 1.06) | 0.767 |
| Time | - | - | - | - | -0.04 (-0.36, 0.29) | 0.824 | -0.16 (-0.49, 0.18) | 0.355 |
| **Random effects** | **Variance** | **SD** | **Variance** | **SD** | **Variance** | **SD** | **Variance** | **SD** |
| Centres | 2.82 | 1.68 | 2.80 | 1.67 | 2.65 | 1.63 | 2.42 | 1.56 |
| Participants within centres | 59.75 | 7.73 | 52.45 | 7.24 | 56.17 | 7.49 | 48.37 | 6.95 |
| Residual | 7.66 | 2.77 | 13.46 | 3.67 | 7.69 | 2.77 | 13.63 | 3.69 |
| For adjusted models, the coefficient for male and in reference to females. For diagnosis, the reference is depression only. | | | | | | | | |

| **Supplementary Table 26.** Multi-level linear mixed effects regression of multiple pain predictors on tobacco use risk scores. | | | | | | | | | | | |
| --- | --- | --- | --- | --- | --- | --- | --- | --- | --- | --- | --- |
|  | | **Unadjusted: Unimputed** | | | | **Unadjusted: Imputed** | | **Adjusted: Unimputed** | | **Adjusted: Imputed** | |
| **Variable** | | **Estimate (95%CI)** | | **p-value** | | **Estimate (95%CI)** | **p-value** | **Estimate (95%CI)** | **p-value** | **Estimate (95%CI)** | **p-value** |
| Serious pain frequency (between) | | 0.55 (-0.27, 1.37) | | 0.188 | | 0.62 (-0.12, 1.35) | 0.101 | 0.84 (0.02, 1.66) | 0.044 | 0.73 (0.01, 1.44) | 0.047 |
| Serious pain frequency (within) | | -0.04 (-0.46, 0.38) | | 0.841 | | 0.06 (-0.37, 0.50) | 0.771 | -0.04 (-0.47, 0.40) | 0.860 | 0.08 (-0.35, 0.52) | 0.712 |
| Pain intensity (between) | | 0.63 (-0.60, 1.86) | | 0.314 | | 0.51 (-0.58, 1.60) | 0.359 | 0.33 (-0.91, 1.56) | 0.601 | 0.25 (-0.81, 1.31) | 0.642 |
| Pain intensity (within) | | 0.93 (0.34,1.52) | | 0.002 | | 1.13 (0.52,1.74) | <0.001 | 0.96 (0.36, 1.56) | 0.002 | 1.09 (0.48, 1.70) | <0.001 |
| Pain limitations (between) | | 0.20 (-0.57, 0.97) | | 0.604 | | 0.44 (-0.25, 1.14) | 0.210 | 0.09 (-0.68, 0.86) | 0.828 | 0.48 (-0.19, 1.15) | 0.163 |
| Pain limitations (within) | | -0.27 (-0.68, 0.13) | | 0.187 | | -0.33 (-0.74, 0.09) | 0.122 | -0.32 (-0.73, 0.10) | 0.133 | -0.35 (-0.76, 0.07) | 0.102 |
| Sex - Male | | - | | - | | - | - | 1.17 (0.15, 2.19) | 0.025 | 0.63 (-0.24, 1.51) | 0.155 |
| Age (years) | | - | | - | | - | - | 0.53 (0.38, 0.68) | <0.001 | 0.51 (0.39, 0.63) | <0.001 |
| Anxiety only | | - | | - | | - | - | -0.90 (-1.75, -0.04) | 0.039 | -1.10 (-1.92, -0.28) | 0.009 |
| Depression and anxiety | | - | | - | | - | - | -0.65 (-1.42, 0.11) | 0.095 | -0.59 (-1.38, 0.20) | 0.144 |
| Other diagnosis | | - | | - | | - | - | 0.09 (-0.79, 0.97) | 0.838 | 0.16 (-0.76, 1.07) | 0.740 |
| Time | | - | | - | | - | - | -0.05 (-0.37, 0.27) | 0.773 | -0.14 (-0.47, 0.20) | 0.418 |
| **Random effects** | | **Variance** | | **SD** | | **Variance** | **SD** | **Variance** | **SD** | **Variance** | **SD** |
| Centres | | 2.73 | | 1.65 | | 2.66 | 1.63 | 2.54 | 1.59 | 2.27 | 1.51 |
| Participants within centres | | 59.56 | | 7.72 | | 52.03 | 7.21 | 55.84 | 7.47 | 47.97 | 6.93 |
| Residual | | 7.58 | | 2.75 | | 13.39 | 3.66 | 7.62 | 2.76 | 13.56 | 3.68 |
| For adjusted models, the coefficient for male and in reference to females. For diagnosis, the reference is depression only. | | | | | | | | | | | |
| **Supplementary Table 27.** Multi-level linear mixed effects regression of serious pain frequency on alcohol use risk scores. | | | | | | | | | | | |
|  | **Unadjusted: Unimputed** | | | | **Unadjusted: Imputed** | | | **Adjusted: Unimputed** | | **Adjusted: Imputed** | |
| **Variable** | **Estimate (95%CI)** | | **p-value** | | **Estimate (95%CI)** | | **p-value** | **Estimate (95%CI)** | **p-value** | **Estimate (95%CI)** | **p-value** |
| Serious pain frequency (between) | 0.21 (-0.31, 0.73) | | 0.433 | | 0.51 (0.06, 0.96) | | 0.025 | 0.29 (-0.21, 0.80) | 0.255 | 0.52 (0.10, 0.95) | 0.016 |
| Serious pain frequency (within) | -0.06 (-0.62, 0.49) | | 0.822 | | 0.04 (-0.40, 0.49) | | 0.852 | -0.10 (-0.65, 0.46) | 0.736 | -0.02 (-0.46, 0.43) | 0.939 |
| Sex - Male | - | | - | | - | | - | -0.29 (-1.20, 0.62) | 0.529 | -0.33 (-1.09, 0.44) | 0.402 |
| Age (years) | - | | - | | - | | - | 0.74 (0.61, 0.87) | <0.001 | 0.71 (0.60, 0.82) | <0.001 |
| Anxiety only | - | | - | | - | | - | -1.47 (-2.53, -0.40) | 0.007 | -1.29 (-2.20, -0.38) | 0.005 |
| Depression and anxiety | - | | - | | - | | - | -0.84 (-1.83, 0.15) | 0.096 | -0.47 (-1.35, 0.41) | 0.297 |
| Other diagnosis | - | | - | | - | | - | -0.32 (-1.41, 0.77) | 0.566 | -0.45 (-1.43, 0.54) | 0.372 |
| Time | - | | - | | - | | - | -0.89 (-1.38, -0.41) | <0.001 | -0.49 (-0.90, -0.09) | 0.017 |
| **Random effects** | **Variance** | | **SD** | | **Variance** | | **SD** | **Variance** | **SD** | **Variance** | **SD** |
| Centres | 0.44 | | 0.66 | | 1.01 | | 1.00 | 0.00 | 0.00 | 0.37 | 0.61 |
| Participants within centres | 41.27 | | 6.42 | | 36.25 | | 6.02 | 35.94 | 6.00 | 30.43 | 5.52 |
| Residual | 18.55 | | 4.31 | | 20.68 | | 4.55 | 18.44 | 4.29 | 20.67 | 4.55 |
| For adjusted models, the coefficient for male and in reference to females. For diagnosis, the reference is depression only. | | | | | | | | | | | |

| **Supplementary Table 28.** Multi-level linear mixed effects regression of pain intensity on alcohol use risk scores. | | | | | | | | |
| --- | --- | --- | --- | --- | --- | --- | --- | --- |
|  | **Unadjusted: Unimputed** | | **Unadjusted: Imputed** | | **Adjusted: Unimputed** | | **Adjusted: Imputed** | |
| **Variable** | **Estimate (95%CI)** | **p-value** | **Estimate (95%CI)** | **p-value** | **Estimate (95%CI)** | **p-value** | **Estimate (95%CI)** | **p-value** |
| Pain intensity (between) | 0.48 (-0.24, 1.19) | 0.191 | 0.80 (0.19, 1.40) | 0.010 | 0.20 (-0.50, 0.90) | 0.579 | 0.61 (0.03, 1.19) | 0.039 |
| Pain intensity (within) | 0.42 (-0.30, 1.13) | 0.256 | 0.78 (0.19, 1.37) | 0.009 | 0.42 (-0.31, 1.15) | 0.255 | 0.67 (0.09, 1.26) | 0.025 |
| Sex - Male | - | - | - | - | -0.30 (-1.21, 0.61) | 0.520 | -0.29 (-1.06, 0.47) | 0.454 |
| Age (years) | - | - | - | - | 0.74 (0.61, 0.87) | <0.001 | 0.71 (0.60, 0.82) | <0.001 |
| Anxiety only | - | - | - | - | -1.44 (-2.50, -0.38) | 0.008 | -1.24 (-2.15, -0.33) | 0.008 |
| Depression and anxiety | - | - | - | - | -0.83 (-1.82, 0.16) | 0.100 | -0.47 (-1.35, 0.41) | 0.294 |
| Other diagnosis | - | - | - | - | -0.27 (-1.36, 0.82) | 0.630 | -0.37 (-1.35, 0.62) | 0.463 |
| Time | - | - | - | - | -0.86 (-1.34, -0.38) | <0.001 | -0.45 (-0.86, -0.05) | 0.028 |
| **Random effects** | **Variance** | **SD** | **Variance** | **SD** | **Variance** | **SD** | **Variance** | **SD** |
| Centres | 0.42 | 0.65 | 0.97 | 0.98 | 0.00 | 0.00 | 0.36 | 0.60 |
| Participants within centres | 41.24 | 6.42 | 36.06 | 6.00 | 36.04 | 6.00 | 30.37 | 5.51 |
| Residual | 18.53 | 4.30 | 20.67 | 4.55 | 18.40 | 4.29 | 20.68 | 4.55 |
| For adjusted models, the coefficient for male and in reference to females. For diagnosis, the reference is depression only. | | | | | | | | |

| **Supplementary Table 29.** Multi-level linear mixed effects regression of pain limitations on alcohol use risk scores. | | | | | | | | |
| --- | --- | --- | --- | --- | --- | --- | --- | --- |
|  | **Unadjusted: Unimputed** | | **Unadjusted: Imputed** | | **Adjusted: Unimputed** | | **Adjusted: Imputed** | |
| **Variable** | **Estimate (95%CI)** | **p-value** | **Estimate (95%CI)** | **p-value** | **Estimate (95%CI)** | **p-value** | **Estimate (95%CI)** | **p-value** |
| Pain limitations (between) | 0.40 (-0.06, 0.87) | 0.085 | 0.65 (0.25, 1.05) | 0.001 | 0.28 (-0.17, 0.73) | 0.221 | 0.58 (0.21, 0.96) | 0.003 |
| Pain limitations (within) | 0.17 (-0.32, 0.66) | 0.490 | 0.33 (-0.07, 0.74) | 0.106 | 0.05 (-0.45, 0.55) | 0.854 | 0.26 (-0.14, 0.67) | 0.202 |
| Sex - Male | - | - | - | - | -0.26 (-1.17, 0.65) | 0.573 | -0.25 (-1.01, 0.52) | 0.528 |
| Age (years) | - | - | - | - | 0.74 (0.60, 0.87) | <0.001 | 0.71 (0.60, 0.82) | <0.001 |
| Anxiety only | - | - | - | - | -1.45 (-2.51, -0.38) | 0.008 | -1.24 (-2.15, -0.34) | 0.007 |
| Depression and anxiety | - | - | - | - | -0.83 (-1.82, 0.16) | 0.101 | -0.46 (-1.34, 0.41) | 0.301 |
| Other diagnosis | - | - | - | - | -0.29 (-1.38, 0.80) | 0.602 | -0.40 (-1.38, 0.59) | 0.429 |
| Time | - | - | - | - | -0.88 (-1.37, -0.39) | <0.001 | -0.46 (-0.87, -0.06) | 0.025 |
| **Random effects** | **Variance** | **SD** | **Variance** | **SD** | **Variance** | **SD** | **Variance** | **SD** |
| Centres | 0.41 | 0.64 | 0.96 | 0.98 | 0.00 | 0.00 | 0.36 | 0.60 |
| Participants within centres | 41.20 | 6.42 | 36.15 | 6.01 | 35.96 | 6.00 | 30.38 | 5.51 |
| Residual | 18.54 | 4.31 | 20.63 | 4.54 | 18.43 | 4.29 | 20.64 | 4.54 |
| For adjusted models, the coefficient for male and in reference to females. For diagnosis, the reference is depression only. | | | | | | | | |

| **Supplementary Table 30.** Multi-level linear mixed effects regression of multiple pain predictors on alcohol use risk scores. | | | | | | | | | | | |
| --- | --- | --- | --- | --- | --- | --- | --- | --- | --- | --- | --- |
|  | | **Unadjusted: Unimputed** | | | | **Unadjusted: Imputed** | | **Adjusted: Unimputed** | | **Adjusted: Imputed** | |
| **Variable** | | **Estimate (95%CI)** | | **p-value** | | **Estimate (95%CI)** | **p-value** | **Estimate (95%CI)** | **p-value** | **Estimate (95%CI)** | **p-value** |
| Serious pain frequency (between) | | -0.23 (-0.99, 0.54) | | 0.557 | | -0.03 (-0.70, 0.65) | 0.942 | 0.26 (-0.49, 1.00) | 0.499 | 0.21 (-0.43, 0.85) | 0.520 |
| Serious pain frequency (within) | | -0.32 (-0.97, 0.33) | | 0.335 | | -0.34 (-0.85, 0.18) | 0.200 | -0.25 (-0.91, 0.40) | 0.444 | -0.32 (-0.83, 0.19) | 0.222 |
| Pain intensity (between) | | 0.19 (-0.96, 1.33) | | 0.750 | | 0.15 (-0.85, 1.15) | 0.765 | -0.44 (-1.56, 0.69) | 0.447 | -0.25 (-1.20, 0.70) | 0.603 |
| Pain intensity (within) | | 0.50 (-0.40, 1.40) | | 0.275 | | 0.77 (0.05, 1.49) | 0.037 | 0.58 (-0.32, 1.49) | 0.208 | 0.65 (-0.07, 1.37) | 0.075 |
| Pain limitations (between) | | 0.45 (-0.26, 1.17) | | 0.216 | | 0.58 (-0.05, 1.22) | 0.073 | 0.33 (-0.37, 1.03) | 0.352 | 0.57 (-0.03, 1.17) | 0.064 |
| Pain limitations (within) | | 0.12 (-0.49, 0.74) | | 0.691 | | 0.19 (-0.30, 0.68) | 0.446 | -0.07 (-0.69, 0.55) | 0.825 | 0.15 (-0.34, 0.64) | 0.550 |
| Sex - Male | | - | | - | | - | - | -0.26 (-1.17, 0.65) | 0.578 | -0.26 (-1.03, 0.50) | 0.500 |
| Age (years) | | - | | - | | - | - | 0.75 (0.61, 0.88) | <0.001 | 0.71 (0.60, 0.82) | <0.001 |
| Anxiety only | | - | | - | | - | - | -1.48 (-2.54, -0.41) | 0.007 | -1.24 (-2.15, -0.34) | 0.007 |
| Depression and anxiety | | - | | - | | - | - | -0.85 (-1.84, 0.14) | 0.093 | -0.48 (-1.36, 0.39) | 0.280 |
| Other diagnosis | | - | | - | | - | - | -0.29 (-1.38, 0.80) | 0.603 | -0.40 (-1.38, 0.59) | 0.431 |
| Time | | - | | - | | - | - | -0.89 (-1.38, -0.40) | <0.001 | -0.47 (-0.88, -0.07) | 0.023 |
| **Random effects** | | **Variance** | | **SD** | | **Variance** | **SD** | **Variance** | **SD** | **Variance** | **SD** |
| Centres | | 0.41 | | 0.64 | | 0.95 | 0.98 | 0.00 | 0.00 | 0.35 | 0.59 |
| Participants within centres | | 41.28 | | 6.42 | | 36.06 | 6.01 | 36.01 | 6.00 | 30.29 | 5.50 |
| Residual | | 18.55 | | 4.31 | | 20.64 | 4.54 | 18.42 | 4.29 | 20.66 | 4.54 |
| For adjusted models, the coefficient for male and in reference to females. For diagnosis, the reference is depression only. | | | | | | | | | | | |
| **Supplementary Table 31.** Multi-level linear mixed effects regression of serious pain frequency on cannabis use risk scores. | | | | | | | | | | | |
|  | **Unadjusted: Unimputed** | | | | **Unadjusted: Imputed** | | | **Adjusted: Unimputed** | | **Adjusted: Imputed** | |
| **Variable** | **Estimate (95%CI)** | | **p-value** | | **Estimate (95%CI)** | | **p-value** | **Estimate (95%CI)** | **p-value** | **Estimate (95%CI)** | **p-value** |
| Serious pain frequency (between) | 0.62 (0.08, 1.17) | | 0.025 | | 1.29 (0.79, 1.78) | | <0.001 | 0.69 (0.14, 1.25) | 0.014 | 1.26 (0.77, 1.75) | <0.001 |
| Serious pain frequency (within) | -0.10 (-0.57, 0.37) | | 0.675 | | 0.31 (-0.14, 0.76) | | 0.177 | -0.15 (-0.64, 0.33) | 0.539 | 0.26 (-0.19, 0.71) | 0.259 |
| Sex - Male | - | | - | | - | | - | 0.37 (-0.64, 1.38) | 0.472 | 0.23 (-0.65, 1.12) | 0.609 |
| Age (years) | - | | - | | - | | - | 0.39 (0.24, 0.54) | <0.001 | 0.39 (0.26, 0.51) | <0.001 |
| Anxiety only | - | | - | | - | | - | -0.07 (-1.08, 0.95) | 0.898 | 0.08 (-0.87, 1.03) | 0.866 |
| Depression and anxiety | - | | - | | - | | - | 0.26 (-0.68, 1.19) | 0.591 | 0.83 (-0.09, 1.75) | 0.078 |
| Other diagnosis | - | | - | | - | | - | 1.15 (0.10, 2.20) | 0.032 | 1.12 (0.07, 2.17) | 0.036 |
| Time | - | | - | | - | | - | -0.61 (-1.03, -0.20) | 0.004 | -0.44 (-0.84, -0.03) | 0.033 |
| **Random effects** | **Variance** | | **SD** | | **Variance** | | **SD** | **Variance** | **SD** | **Variance** | **SD** |
| Centres | 1.24 | | 1.11 | | 1.57 | | 1.25 | 1.29 | 1.14 | 1.63 | 1.28 |
| Participants within centres | 51.90 | | 7.20 | | 47.80 | | 6.91 | 50.70 | 7.12 | 45.55 | 6.75 |
| Residual | 13.36 | | 3.65 | | 20.16 | | 4.49 | 13.46 | 3.67 | 20.25 | 4.50 |
| For adjusted models, the coefficient for male and in reference to females. For diagnosis, the reference is depression only. | | | | | | | | | | | |

| **Supplementary Table 32.** Multi-level linear mixed effects regression of pain intensity on cannabis use risk scores. | | | | | | | | |
| --- | --- | --- | --- | --- | --- | --- | --- | --- |
|  | **Unadjusted: Unimputed** | | **Unadjusted: Imputed** | | **Adjusted: Unimputed** | | **Adjusted: Imputed** | |
| **Variable** | **Estimate (95%CI)** | **p-value** | **Estimate (95%CI)** | **p-value** | **Estimate (95%CI)** | **p-value** | **Estimate (95%CI)** | **p-value** |
| Pain intensity (between) | 0.80 (0.04, 1.55) | 0.038 | 1.40 (0.73, 2.08) | <0.001 | 0.79 (0.02, 1.55) | 0.045 | 1.29 (0.62, 1.96) | <0.001 |
| Pain intensity (within) | 0.42 (-0.20, 1.05) | 0.186 | 0.89 (0.29, 1.48) | 0.003 | 0.43 (-0.21, 1.07) | 0.190 | 0.81 (0.21, 1.41) | 0.008 |
| Sex - Male | - | - | - | - | 0.37 (-0.64, 1.39) | 0.468 | 0.26 (-0.63, 1.15) | 0.562 |
| Age (years) | - | - | - | - | 0.39 (0.24, 0.54) | <0.001 | 0.37 (0.25, 0.50) | <0.001 |
| Anxiety only | - | - | - | - | -0.01 (-1.03, 1.01) | 0.981 | 0.14 (-0.81, 1.09) | 0.771 |
| Depression and anxiety | - | - | - | - | 0.26 (-0.67, 1.20) | 0.583 | 0.83 (-0.09, 1.75) | 0.078 |
| Other diagnosis | - | - | - | - | 1.21 (0.16, 2.27) | 0.024 | 1.23 (0.19, 2.28) | 0.021 |
| Time | - | - | - | - | -0.58 (-1.00, -0.17) | 0.006 | -0.42 (-0.82, -0.02) | 0.041 |
| **Random effects** | **Variance** | **SD** | **Variance** | **SD** | **Variance** | **SD** | **Variance** | **SD** |
| Centres | 1.25 | 1.12 | 1.65 | 1.28 | 1.30 | 1.14 | 1.71 | 1.31 |
| Participants within centres | 52.03 | 7.21 | 48.25 | 6.95 | 50.90 | 7.13 | 46.09 | 6.79 |
| Residual | 13.32 | 3.65 | 20.08 | 4.48 | 13.43 | 3.66 | 20.18 | 4.49 |
| For adjusted models, the coefficient for male and in reference to females. For diagnosis, the reference is depression only. | | | | | | | | |

| **Supplementary Table 33.** Multi-level linear mixed effects regression of pain limitations on cannabis use risk scores. | | | | | | | | |
| --- | --- | --- | --- | --- | --- | --- | --- | --- |
|  | **Unadjusted: Unimputed** | | **Unadjusted: Imputed** | | **Adjusted: Unimputed** | | **Adjusted: Imputed** | |
| **Variable** | **Estimate (95%CI)** | **p-value** | **Estimate (95%CI)** | **p-value** | **Estimate (95%CI)** | **p-value** | **Estimate (95%CI)** | **p-value** |
| Pain limitations (between) | 0.50 (0.02, 0.99) | 0.042 | 0.96 (0.52, 1.40) | <0.001 | 0.79 (0.02, 1.55) | 0.045 | 1.29 (0.62, 1.96) | <0.001 |
| Pain limitations (within) | 0.17 (-0.25, 0.60) | 0.422 | 0.57 (0.17, 0.98) | 0.006 | 0.43 (-0.21, 1.07) | 0.190 | 0.81 (0.21, 1.41) | 0.008 |
| Sex - Male | - | - | - | - | 0.37 (-0.64, 1.39) | 0.468 | 0.26 (-0.63, 1.15) | 0.562 |
| Age (years) | - | - | - | - | 0.39 (0.24, 0.54) | <0.001 | 0.37 (0.25, 0.50) | <0.001 |
| Anxiety only | - | - | - | - | -0.01 (-1.03, 1.01) | 0.981 | 0.14 (-0.81, 1.09) | 0.771 |
| Depression and anxiety | - | - | - | - | 0.26 (-0.67, 1.20) | 0.583 | 0.83 (-0.09, 1.75) | 0.078 |
| Other diagnosis | - | - | - | - | 1.21 (0.16, 2.27) | 0.024 | 1.23 (0.19, 2.28) | 0.021 |
| Time | - | - | - | - | -0.58 (-1.00, -0.17) | 0.006 | -0.42 (-0.82, -0.02) | 0.041 |
| **Random effects** | **Variance** | **SD** | **Variance** | **SD** | **Variance** | **SD** | **Variance** | **SD** |
| Centres | 1.29 | 1.13 | 1.70 | 1.31 | 1.30 | 1.14 | 1.71 | 1.31 |
| Participants within centres | 52.01 | 7.21 | 48.25 | 6.95 | 50.90 | 7.13 | 46.09 | 6.79 |
| Residual | 13.34 | 3.65 | 20.07 | 4.48 | 13.43 | 3.66 | 20.18 | 4.49 |
| For adjusted models, the coefficient for male and in reference to females. For diagnosis, the reference is depression only. | | | | | | | | |

| **Supplementary Table 34.** Multi-level linear mixed effects regression of multiple pain predictors on cannabis use risk scores. | | | | | | | | |
| --- | --- | --- | --- | --- | --- | --- | --- | --- |
|  | **Unadjusted: Unimputed** | | **Unadjusted: Imputed** | | **Adjusted: Unimputed** | | **Adjusted: Imputed** | |
| **Variable** | **Estimate (95%CI)** | **p-value** | **Estimate (95%CI)** | **p-value** | **Estimate (95%CI)** | **p-value** | **Estimate (95%CI)** | **p-value** |
| Serious pain frequency (between) | 0.37 (-0.44, 1.18) | 0.375 | 1.00 (0.25, 1.75) | 0.009 | 0.53 (-0.30, 1.35) | 0.210 | 1.07 (0.33, 1.81) | 0.004 |
| Serious pain frequency (within) | -0.33 (-0.88, 0.22) | 0.240 | -0.04 (-0.56, 0.47) | 0.869 | -0.36 (-0.93, 0.20) | 0.209 | -0.04 (-0.56, 0.47) | 0.866 |
| Pain intensity (between) | 0.27 (-0.95, 1.48) | 0.668 | 0.13 (-0.98, 1.23) | 0.821 | 0.13 (-1.11, 1.37) | 0.834 | -0.08 (-1.17, 1.01) | 0.886 |
| Pain intensity (within) | 0.49 (-0.28, 1.26) | 0.214 | 0.56 (-0.17, 1.28) | 0.132 | 0.57 (-0.22, 1.36) | 0.157 | 0.49 (-0.24, 1.22) | 0.185 |
| Pain limitations (between) | 0.16 (-0.60, 0.92) | 0.679 | 0.28 (-0.42, 0.98) | 0.436 | 0.13 (-0.64, 0.90) | 0.745 | 0.30 (-0.39, 0.99) | 0.398 |
| Pain limitations (within) | 0.12 (-0.41, 0.65) | 0.665 | 0.34 (-0.15, 0.84) | 0.174 | 0.04 (-0.51, 0.58) | 0.893 | 0.31 (-0.18, 0.81) | 0.217 |
| Sex - Male | - | - | - | - | 0.39 (-0.62, 1.41) | 0.446 | 0.27 (-0.62, 1.16) | 0.552 |
| Age (years) | - | - | - | - | 0.39 (0.24, 0.54) | <0.001 | 0.39 (0.26, 0.51) | <0.001 |
| Anxiety only | - | - | - | - | -0.03 (-1.05, 0.99) | 0.951 | 0.13 (-0.82, 1.08) | 0.788 |
| Depression and anxiety | - | - | - | - | 0.25 (-0.68, 1.19) | 0.597 | 0.81 (-0.11,1 .73) | 0.086 |
| Other diagnosis | - | - | - | - | 1.20 (0.14, 2.25) | 0.026 | 1.16 (0.11, 2.21) | 0.030 |
| Time | - | - | - | - | -0.60 (-1.02, -0.18) | 0.005 | -0.41 (-0.81, 0.00) | 0.050 |
| **Random effects** | **Variance** | **SD** | **Variance** | **SD** | **Variance** | **SD** | **Variance** | **SD** |
| Centres | 1.23 | 1.11 | 1.56 | 1.25 | 1.27 | 1.13 | 1.61 | 1.27 |
| Participants within centres | 52.08 | 7.22 | 47.95 | 6.92 | 50.90 | 7.13 | 45.69 | 6.76 |
| Residual | 13.32 | 3.65 | 20.06 | 4.48 | 13.42 | 3.66 | 20.17 | 4.49 |
| For adjusted models, the coefficient for male and in reference to females. For diagnosis, the reference is depression only. | | | | | | | | |
